# Bioorthogonal Fluorogenic Reporters for Noninvasive Imaging and Urinalysis of Immunotherapeutic Response in Renal Cell Carcinoma

**DOI:** 10.1002/advs.202524298

**Published:** 2026-04-24

**Authors:** Xingyue Yang, Baoshuai Liang, Liangmin Fu, Weiliang Deng, Yuyan Jiang, Weiping Xu, Jinwei Chen, Ya Zhou, Ke Wu, Jiaguo Huang

**Affiliations:** ^1^ State Key Laboratory of Anti‐Infective Drug Discovery and Development School of Pharmaceutical Sciences Sun Yat‐sen University Guangzhou China; ^2^ Department of Pharmacy The Affiliated Dazu's Hospital of Chongqing Medical University Chongqing China; ^3^ Department of Urology The Second Xiangya Hospital of Central South University Changsha China; ^4^ Department of Urology The First Affiliated Hospital of Sun Yat‐sen University Guangzhou China; ^5^ Department of Radiation Oncology Stanford University School of Medicine Stanford California USA

**Keywords:** bioorthogonal chemistry, immunotherapy, NIRF imaging, renal cell carcinoma, urinalysis

## Abstract

Real‐time monitoring of kidney‐infiltrating cytotoxic T lymphocytes (CTLs) is crucial for evaluating immunotherapy in renal cell carcinoma (RCC). However, existing imaging probes often exhibit “always‐on” signals and poor renal clearance, limiting their ability to detect renal immune responses. Herein, we report renal‐clearable bio‐orthogonal near‐infrared (NIR) fluorogenic probes (BGRs) that specifically detect granzyme B (GzmB), a biomarker of CTL activation, for dynamic evaluation of RCC immunotherapy. BGRs are built on a nitrile‐substituted hemicyanine scaffold with a biothiol‐responsive cysteine tail that is dually locked with a GzmB‐responsive peptide and conjugated to renal clearable (2‐hydroxypropyl)‐β‐cyclodextrin (HPβCD). Following injection in RCC mouse models under immunotherapy, non‐fluorescent BGR_M_ accumulates in the renal tumor, where dual cleavage by elevated glutathione (GSH) and GzmB releases HPβCD and exposes an aminothiol group, triggering nitrile‐aminothiol biorthogonal click reaction that activates NIR fluorescence and drives probe self‐assembly for enhanced imaging performance. BGRs not only differentiate immunotherapeutic responses in living mice but also enable sensitive optical urinalysis of GzmB in clinical specimens from RCC patients (n = 21), allowing precise stratification of immune activation before and after treatment. This work thus establishes a generalizable strategy for translational optical reporters that addresses the unmet clinical need for dynamic monitoring of immunotherapies in RCC and other urological cancers.

## Graphical abstract

This study reported renal‐clearable bio‐orthogonal near‐infrared fluorogenic probes (BGRs) that specifically imaging and urinalysis of granzyme B for dynamic evaluation of RCC immunotherapy. BGRs not only differentiate immunotherapeutic responses in orthotopic RCC mice, but also enable sensitive optical urinalysis of granzyme B in clinical specimens from RCC patients, allowing precise stratification of immune activation before and after treatment.

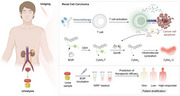

## 1 Introduction

Immunotherapies have ushered in a new era of cancer treatments by activating the host immune system to target and destroy tumors [1]. With innovative approaches such as cancer vaccines, immune checkpoint blockade, and chimeric antigen receptor (CAR) T‐cell therapy, immunotherapies have significantly improved survival across various cancer types [2, 3]. However, due to the complexity of immune evasion and the heterogeneous nature of immunosuppressive tumor microenvironment (TME), only a subset of patients benefits, leading to relatively low objective response rates (<30%) [4, 5]. Furthermore, immunotherapy can cause diverse immune‐related adverse events, including hepatitis, colitis, pulmonary toxicities, and in severe cases, death [6]. Therefore, reliable strategies to timely monitor patient responses and distinguish responders from non‐responders at an early stage are urgently needed, which can not only minimize unnecessary side effects but also enable the timely redirection of non‐responders to alternative treatments.

Despite the critical demand, dynamic monitoring of immune responses using current diagnostic approaches, such as biopsies and bioimaging, faces significant limitations [7]. Biopsy‐based methods such as immunohistochemistry (IHC) staining of biomarkers, tumor mutational burden (TMB) analysis, and immunophenotyping are inherently restricted by their static nature and sampling bias [6]. This makes them unable to accurately capture the heterogeneity of immune infiltrates within tumors. Their invasive nature also prevents repetitive use for longitudinal monitoring [8, 9, 10]. Although bioimaging offers unparalleled advantages for non‐invasive, real‐time, dynamic, and longitudinal evaluation of immuno‐oncology, conventional imaging modalities like computed tomography (CT) and magnetic resonance imaging (MRI) are typically limited to anatomical analyses such as tumor mass change and fail to indicate immune cell infiltration [11, 12]. While immuno‐positron emission tomography (immunoPET) using radiolabeled antibodies or peptides has been developed to track immune cells, it cannot discriminate between the active and indolent immune cells or indicate their functional states [13, 14, 15]. This fundamental limitation undermines its ability to accurately assess therapeutic efficacy [13, 14, 16]. Moreover, the short half‐life of PET radiotracers poses a practical challenge for monitoring slow immune responses, often requiring multiple administrations and repetitive imaging—a regimen that is both resource‐intensive and clinically inconvenient [13, 14, 17].

Direct imaging of cytotoxic T lymphocyte (CTL) activity in the TME presents a more effective and sensitive measure of immunotherapeutic responses than monitoring morphological or anatomical changes [15, 18, 19, 20, 21, 22]. Granzyme B (GzmB), a cytotoxic serine protease released by activated CTLs and natural killer (NK) cells upon target cell recognition, is a pivotal biomarker for this purpose [23]. Its secretion not only reflects the presence and localization of active CTLs but also directly indicates their tumor‐killing ability [24, 25]. While several peptidomimetic‐based PET tracers have been reported to monitor GzmB activity in syngeneic colon cancer models and in patients with lung adenocarcinoma [21, 22], these probes typically generate “always‐on” signals and predominantly go through renal clearance [26, 27, 28, 29, 30, 31, 32, 33, 34]. The inevitable nonspecific accumulation in the kidneys makes it extremely challenging to track GzmB and monitor immunotherapy in renal cell carcinoma (RCC), where immunotherapy has become a standard of care. Different from “always‐on” probes, activity‐based optical probes, which turn on their signals only upon target recognition, offer much higher sensitivity and specificity for detecting subtle changes of biomarkers at pathological sites [35]. Although activatable probes have been reported to image GzmB in other cancers (e.g., colorectal cancer, lymphoma, breast cancer) and in allograft rejection (e.g., skin and liver transplantation) [35, 36, 37, 38, 39, 40, 41], their application in monitoring RCC immunotherapy has remained unexplored due to insufficient kidney targeting, representing a critical research and clinical gap.

**FIGURE 1:**
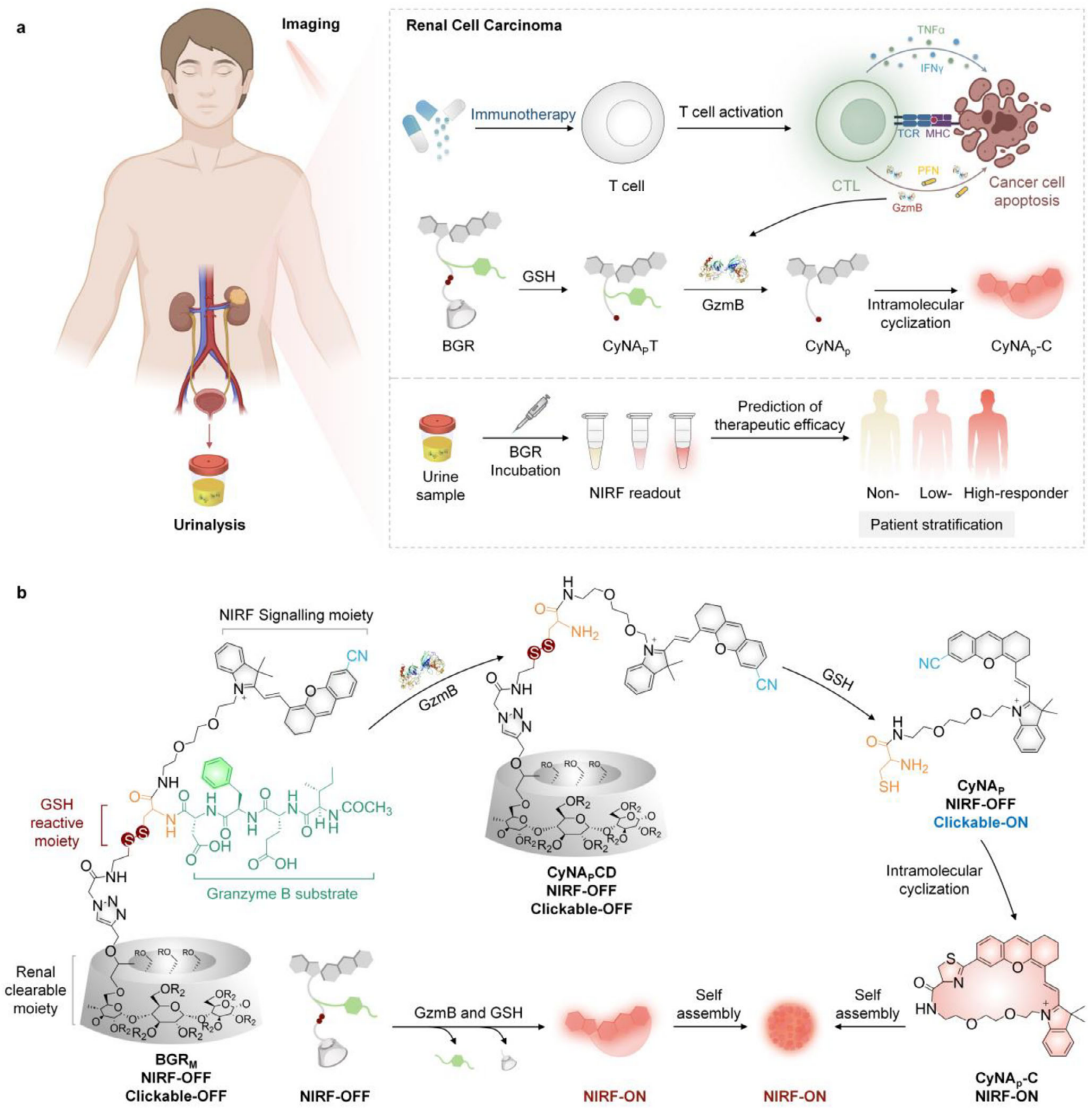
Design and mechanisms of the bioorthogonal NIR fluorogenic probes (BGRs) for molecular imaging and urinalysis of immunotherapeutic response in RCC. (a) Schematic diagram of immunotherapy in RCC and the detection methods used here, including molecular imaging and optical urinalysis (collection of urine followed by incubation with BGRs and NIRF readout). The image of a human was created with BioRender.com. (b) Molecular structures and sensing mechanism of BGR_M_ with GzmB/GSH‐triggered intramolecular cyclization and self‐assembly.

Herein, we report the development of **B**ioorthogonal **G**ranzyme **R**eporters (**BGRs**) with fluorogenic response and efficient renal clearance for non‐invasive near‐infrared fluorescence (NIRF) imaging and urinalysis of immunotherapeutic response in RCC (Figure 1a). BGRs comprise a NIRF signaling scaffold dually locked by a GzmB‐responsive peptide and a tumor‐associated biothiol (glutathione, GSH)‐cleavable unit, which further hooks a renal clearable moiety ((2‐hydroxypropyl)‐β‐cyclodextrin (HPβCD) (Figure 1b). Using two different peptide substrates, N‐acetyl‐Ile‐Glu‐Phe‐Asp (IEFD) and N‐acetyl‐Ile‐Glu‐Pro‐Asp (IEPD), we prepared two probes, BGR_M_ and BGR_H_, which are respectively tailored to detect mouse and human GzmB. In the intrinsic state, BGRs going through the kidneys are non‐fluorescent due to the inhibited intramolecular charge transfer (ICT) process (Figure 1b). During immunotherapy of RCC, BGRs in the kidneys are cleaved by the upregulated GzmB from activated CTLs, while elevated GSH from TME breaks the disulfide bond to detach the hydrophilic HPβCD. This dual action exposes residues that spontaneously undergo nitrile‐aminothiol (NAT) biorthogonal click reaction for intramolecular cyclization, resulting in NIRF ‘turn‐on’ and simultaneous self‐assembly to nanoaggregates for prolonged retention and enhanced in vivo imaging. In this study, we first construct BGR_M_ and validate its capability for real‐time intravital imaging of GzmB and evaluate diverse immunotherapies in a murine orthotopic RCC model. We further report GzmB as a reliable urinary biomarker of RCC immunotherapy by analyzing murine RCC models and RCC patient samples (n = 21). At last, we demonstrate the feasibility of BGR_H_ to detect urinary GzmB via point‐of‐care optical urinalysis of clinical specimens.

## 2 Results and Discussions

### 2.1 Synthesis and In Vitro Detection

**FIGURE 2:**
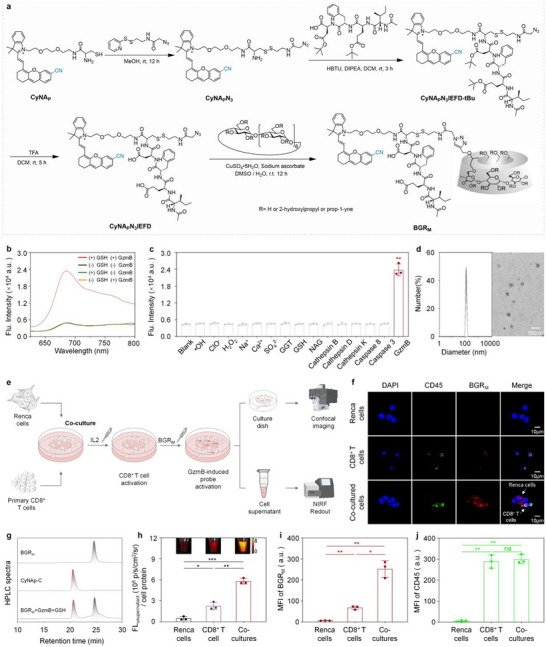
Synthesis and characterization of BGR_M_. (a) Synthesis routes of BGR_M_. (b) Fluorescence spectra of BGR_M_ (20 µm) in the absence and presence of individual biomarkers or combined biomarkers at 37 °C in PBS buffer (pH 7.4). Fluorescence excitation at 580 nm. (c) Fluorescence intensity of BGR_M_ (20 µm) after incubation with the indicated enzymes (0.5 U mL^−1^), metal ions (60 µM), ROS (60 µM) in PBS (10 mM, pH 7.4) at 37 °C. OH·, hydroxyl radical; ClO^−^, sodium hypochlorite; H_2_O_2_, hydrogen peroxide; Na^+^, sodium chloride; Ca^2+^, calcium chloride; SO_4_^2−^, sodium sulfate; GGT, gamma‐glutamyl transferase, NAG, N‐acetyl‐beta‐β‐glucosaminidase (n = 3, mean ± s.d.). GSH/GzmB co‐treated group versus blank group. (d) Representative TEM image and DLS results of BGR_M_ after incubation with GSH/GzmB in buffer solution (scale bar, 150 nm). (e) Schematic illustration of experiment implementation for BGR_M_ to image GzmB in living cells. (f) Representative confocal fluorescence images of Renca cells and CD8^+^ T cells with the incubation of BGR_M_ after the treatment with IL2. Blue fluorescence was from the cell nucleus stained with DAPI, green fluorescence indicated the signals from CD45 stained with CD8^+^ T cells, and red fluorescence indicated the signals from activated BGR_M_. (g) Evaluation of enzymatic cleavage of BGR_M_ through HPLC analysis. HPLC traces of the pure compound CyNA_P_ are also shown for comparison. (h) NIRF imaging of cell supernatant after incubation with BGR_M_ (n = 3, mean ± s.d.). (i,j) Corresponding mean fluorescence intensity of BGR_M_ cells in the panel f (n = 3, mean ± s.d.). Two‐tailed Student's t test, ^*^ *p* < 0.05, ^**^ *p* < 0.01, ^***^ *p* < 0.001, ns, no statistically significant differences.

BGRs were comprised of four components (Figure 1b): a NAT bioorthogonal fluorogenic chromophore (CyNA_P_) as NIRF signaling moiety, a GSH‐responsive disulfide bond, a GzmB‐reactive peptide moiety, and the alkyne‐functionalized HPβCD as renal clearable moiety. The synthetic routes were shown in Figure 2a and Figures S1 and S2. Briefly, alkyne‐functionalized peptides with different sequences, respectively responsive to mouse (N‐acetyl‐Ile‐Glu‐Phe‐Asp, IEFD) and human (N‐acetyl‐Ile‐Glu‐Pro‐Asp, IEPD) GzmB were synthesized via the standard fluorenylmethyloxy‐carbonyl (Fmoc) solid‐phase peptide synthesis (SPPS) method. Nitrile‐substituted hemicyanine CyNA_P_ was synthesized according to our previous studies [34]. It was then coupled with the dithiolated azide derivative to yield CyNA_P_N_3_, which was further conjugated with the protected IEFD or IEPD substrate, followed by deprotection to obtain CyNA_P_N_3_IEFD or CyNA_P_N_3_IEPD, respectively. The resulted compounds were conjugated with alkyne‐functionalized HPβCD to final yield BGR_M_ or BGR_H_, respectively (Figures S1 and S2). All the intermediates and probes were characterized by NMR spectra and mass spectrometry.

To investigate the responses of BGR_M_ toward GSH and mouse GzmB, the optical characteristics were examined in the absence and presence of both biomarkers. In the absence of GSH and mouse GzmB, BGR_M_ exhibited the absorption peak at ∼550 nm (Figure S3) and was nonfluorescent (Figure 2b). However, upon exposure to both GSH and mouse GzmB, BGR_M_ had a red‐shifted absorption maximum at ∼580 nm and a 5‐fold fluorescence enhancement at 675 nm (Figure 2b), indicating probe activation. In contrast, no fluorescence enhancement was observed for BGR_M_ when only one biomarker was present. Furthermore, no significant NIRF change was observed in the presence of other analytes, including reactive oxygen species, reductant, or enzymes, demonstrating the high specificity of BGR_M_ (Figure 2c). No significant fluorescence change of BGR_M_ was observed when stored in both PBS and FBS for 24 h (Figure S4), implying its high stability. The enzymatic cleavage and intramolecular cyclization on BGR_M_ were further studied via high‐performance liquid chromatography (HPLC) analysis (Figure 2g), showing a new HPLC peak at a retention time of about 20.6 min, which corresponded to the cyclized product CyNA_P_‐C. Moreover, in situ formation of nanoaggregates for CyNA_P_‐C was confirmed by transmission electron microscopy (TEM) and dynamic light scattering (DLS) assays (Figure 2d), showing nanoparticles with a spherical morphology and an average diameter of ∼100 nm. Such a spontaneous self‐assembly was ascribed to the increased rigidity and high hydrophobicity of CyNA_P_‐C (Log D = 3.33) compared to BGR_M_ (Log D = −15.87). Similarly, the response of BGR_H_ toward GSH and human GzmB was also investigated (Figure S5).

Considering the endogenous free cysteine in cells and plasma with a concentration between 20 to 100 µm [42], the specificity of intramolecular cyclization on the GzmB/GSH dual‐cleaved product CyNA_P_ was investigated in the presence of high concentration of free cysteine (Figure S6). As such, the mixture of CyNA_P_ with cystine at a 1:100 molar ratio was traced by HPLC. As shown in Figure S6, only one HPLC peak with a retention time of about 20.6 min corresponding to the intramolecular cyclized product (CyNA_P_‐C) was observed, rather than the intermolecular condensation product CyNA_P_‐C_F_, demonstrating that intramolecular condensation occurs preferentially over intermolecular condensation. This was ascribed to the first‐order reaction rate constants (∼8 × 10^−5^ s^−1^) of CyNA_P_ allows for promoting intramolecular cyclization preferentially according to our previous studies [34].

The abilities of BGR_M_ to detect GranB in cells were investigated. Mouse CD8^+^ T cells were first extracted from the mouse spleen according to previous studies [14]. We then cultured Renca tumor cells, or mouse CD8^+^ T cells or co‐cultured both together, and incubated with interleukin IL‐2 for reinvigoration of CD8^+^ T cells (Figure 2e). Next, we treated all three cultured cells with probe BGR_M_ and a surface marker for leukocytes (CD45) before confocal imaging. As shown in Figure 2f, NIRF signals of BGR_M_ were clearly observed in the cytoplasm of both CD8^+^ T cells and Renca cells, confirming that the intracellular activation of probes. However, Renca cells alone showed negligible fluorescence, confirming its specificity for cellular imaging. The activation of BGR_M_ in cultured cells was further confirmed by imaging of cell supernatant (Figure 2h), which showed a similar tendency with confocal cellular imaging results (Figure 2i,j). Such observations coincided with the fact that the leakage of GzmB from granules can occur during storage, activation, or degranulation.

### 2.2 In Vivo Biodistribution and Biocompatibility Studies

**FIGURE 3:**
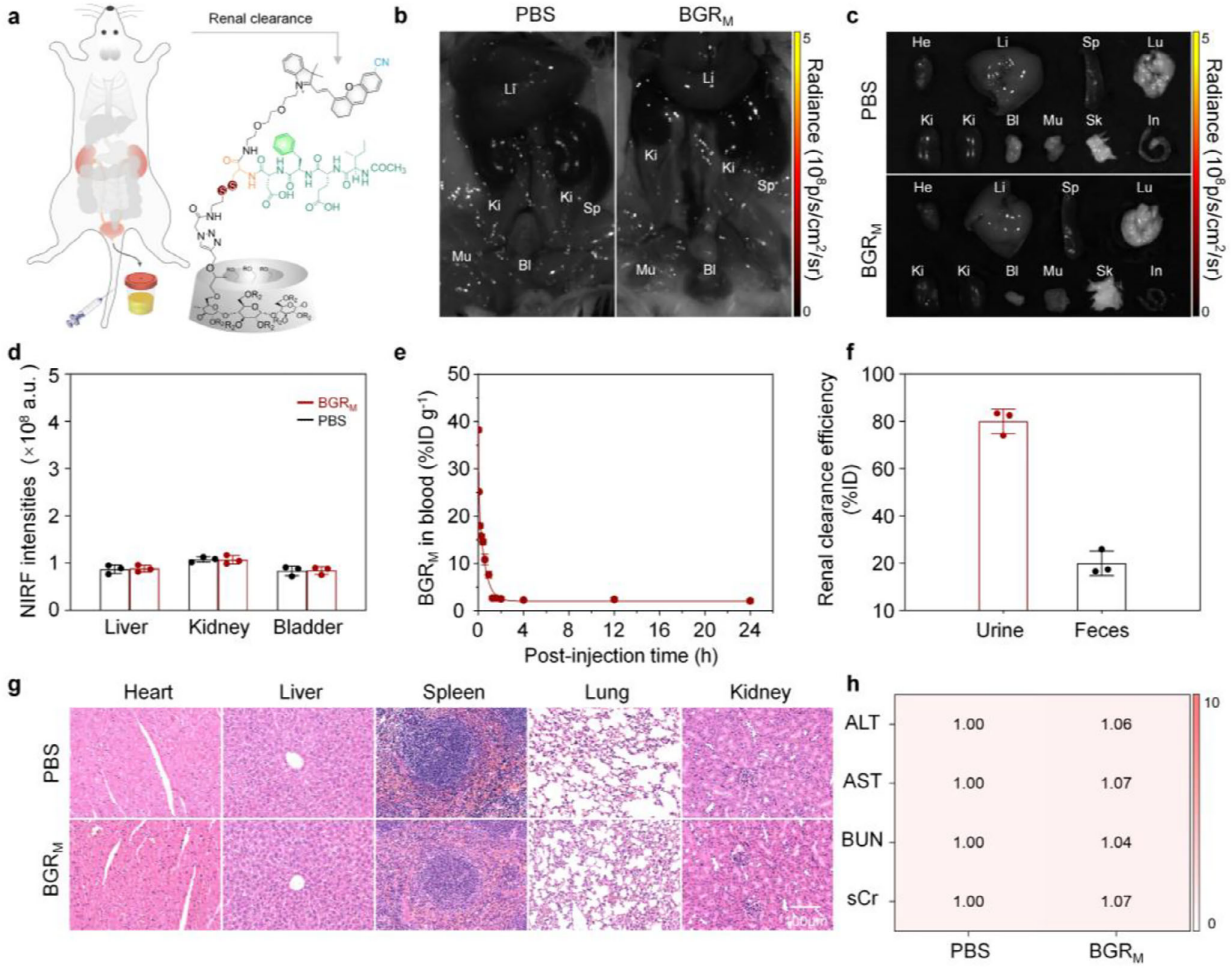
Biodistribution and biocompatibility. (a) Schematic illustration of the renal clearance pathway of BGR_M_ in living mice. (b,c) Representative NIRF images of the abdominal cavity and resected organs at 24 h post‐injection of PBS or BGR_M_ (10 µmol kg^−1^ body weight). He: heart; Li: liver; Sp: spleen; Lu: lung; Ki: kidney; Bl: bladder; Mu: muscle; Sk: skin; In: intestine. (d) *Ex vivo* NIRF quantification of liver, kidney, and bladder at t 24 h post‐injection of PBS or BGR_M_. The NIRF images were acquired at 675 nm upon excitation at 580 nm with the IVIS spectrum imaging system. (e) Blood concentration (% ID g^−1^) decay of BGR_M_ after injection into living mice (n = 3, mean ± s.d.). (f) The renal clearance efficiency of BGR_M_ after 24 h injection. (g) H&E staining of major organs, including heart, liver, spleen, lung, and kidney from Balb/c mice after 24 h injection of PBS or BGR_M_ (10 µmol kg^−1^ body weight). (Scale bar: 100 µm). The experiments were repeated independently three times with similar results. (h) Measurements of liver and kidney functions in Balb/c mice after 24 h injection of PBS or BGR_M_ (n = 3, mean ± s.d.).

The biodistribution and pharmacokinetics of BGR_M_ were studied in healthy living mice (Figure 3a). At 1 h post BGR_M_ injection, minimal NIRF signals were observed across organs, which were as low as those of PBS‐injected mice from quantitative analysis (Figure 3b–d). This was likely due to BGR_M_ was intrinsically non‐fluorescent. Pharmacokinetic studies of BGR_M_ after intravenous (iv) injection were carried out using HPLC. The concentration of BGR_M_ in the blood decreased to nearly 0% of the injected dose (ID) g^−1^ at 1 h post‐injection, and the elimination half‐life (*t*_1/2β_) was estimated to be ∼20 min based on a two‐compartment fitting of plasma clearance kinetics (Figure 3e). Moreover, the renal and fecal clearance efficiencies of BGR_M_ were respectively determined to be ∼80±2% and ∼18±1% ID at 24 h post‐injection (Figure 3f). The high renal clearance of BGR_M_ is attributed to its high hydrophilicity (Log D = −15.87) and its molecular weight being much lower than the glomerular filtration cutoff (<50 kDa). Notably, Hematoxylin and Eosin (H&E) staining of major organs revealed that BGR_M_ did not cause histological changes, affirming its excellent biosafety (Figure 3g). The blood biochemical tests showed no increment in the levels of alanine transaminase (ALT), aspartate transaminase (AST), serum creatinine (sCr), and blood urea nitrogen (BUN) between PBS and BGR_M_‐injected mice (Figure 3h and Figure S7), further confirming its high biocompatibility.

### 2.3 In Vivo NIRF Imaging of Immunotherapeutic Response in Orthotopic RCC Mice

**FIGURE 4:**
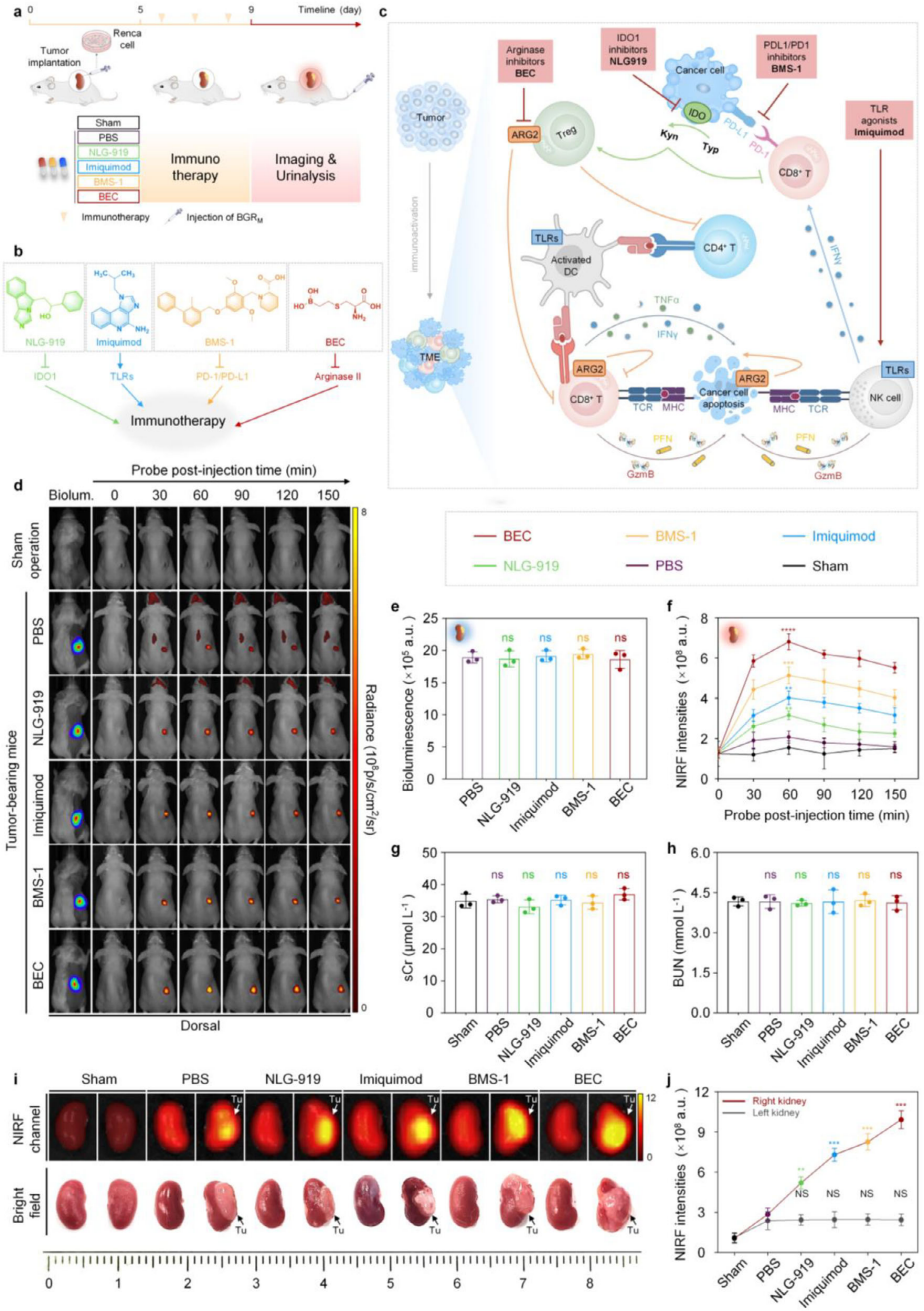
Real‐time NIRF imaging of response to immunotherapy in orthotopic RCC mice. (a) Schematic illustration of the timeline of immunotherapy and NIRF imaging/urinalysis to monitor treatment efficacy. Immunotherapeutics (20 mg kg^−1^ body weight) were intravenously administered into living mice once every day for three times. BGR_M_ (10 µmol kg^−1^ body weight) was injected into mice at 9 days post tumor implantation, and real‐time NIRF imaging was conducted. (b) Chemical structures of different immunotherapeutics for treatment. (c) Schematic diagram of the mechanisms of immunoactivation with different immunotherapeutics. (d) Representative bioluminescence and NIRF images of living mice with injection of BGR_M_ after administration of different immunotherapeutics. NIRF images were acquired at 675 nm with excitation at 580 nm by an IVIS spectrum imaging system. Bioluminescence images acquired under the bioluminescence mode of the IVIS spectrum imaging system with an acquisition time of 3 s. (e) Bioluminescence intensities of tumor‐bearing kidneys at 5 days post tumor implantation (n  =  3, mean ± s.d.). Two‐tailed Student's t‐test; PBS versus different immunotherapeutic groups. ns, no statistically significant differences. (f) The dynamic NIRF intensities of tumor‐bearing kidneys in living mice with injection of BGR_M_ after administration of different immunotherapeutics. (n  =  3, mean ± s.d.). Two‐tailed Student's t‐test; PBS versus different immunotherapeutic groups. (g,h) The levels of sCr and BUN from mice after various treatments (n  =  3, mean ± s.d.). Two‐tailed Student's t‐test; Sham‐operation group versus different treatment groups. ns, no statistically significant differences. (i) Bright field and NIRF images of resected kidneys (left and right kidney) from mice with injection of BGR_M_ after administration of different immunotherapeutics. (j) NIRF intensities of resected kidneys in panel i (n  =  3, mean ± s.d.). Two‐tailed Student's t‐test; PBS versus different immunotherapeutic groups. ^**^ *p* < 0.01, ^***^ *p* < 0.001, ^****^ *p* < 0.0001, ns, no statistically significant differences.

The capability of BGR_M_ for in vivo real‐time imaging of immunotherapeutic response was evaluated in an orthotopic RCC murine model (Figure 4a–c), which was established according to previous studies [43, 44, 45]. Briefly, RENCA^LUC^ cells were implanted into the kidney capsule, and tumor growth was monitored by bioluminescent imaging (Figure 4d). Histological studies confirmed the orthotopic tumor in different immunotherapeutic groups (Figure S8). Four immunotherapeutic agents, namely NLG919, imiquimod, BMS‐1, and S‐(2‐boronoethyl)‐L‐cysteine hydrochloride (BEC), were administered to living mice to activate immune responses (Figure 4b). As an inhibitor of indoleamine 2,3‐dioxygenase, NLG919 plays a critical role in modulating immunosuppressive effects within the tumor microenvironment (TME) by mediating the conversion of tryptophan to kynurenine (Figure 4c). Imiquimod, a specific agonist of Toll‐like receptor 7 (TLR‐7), can induce cytokine secretion, thereby promoting the activation of cytotoxic T lymphocytes (CTLs). BMS‐1 is a potent inhibitor that blocks the interaction between programmed cell death protein 1 (PD‐1) and programmed cell death ligand 1 (PD‐L1)—a pathway that otherwise impairs T cell function and suppresses immune responses. Given that arginase‐mediated depletion of L‐arginine strongly inhibits T cell immune responses, arginase inhibition using BEC is proposed as a strategy to enhance anti‐tumor immunity by facilitating T cell activation and proliferation. The control groups included tumor‐bearing mice treated with PBS or mice that underwent the same surgical procedure with only trauma but without tumor cell implantation (termed the sham‐operation group).

Following 3 cycles of immunotherapeutic treatment, whole‐body longitudinal NIRF imaging was conducted at different timepoints after intravenous injection of BGR_M_ (Figure 4a). As shown in Figure 4d, a negligible NIRF signal was observed in both kidneys after injection of BGR_M_ in mice subjected to sham‐operation. In PBS‐treated tumor‐bearing mice, the tumor‐bearing kidney was clearly delineated by NIRF imaging, while the contralateral kidney was invisible during the entire imaging course. This suggested the activation of BGR_M_ by basal levels of GzmB in the tumor‐bearing kidney. Moreover, the administration of immunotherapeutic drugs led to a gradual NIRF signal increase in tumor‐bearing kidneys (Figure 4d–f). Strong NIRF signals were observed in tumor‐bearing kidneys of immunotherapeutics‐treated mice, which were 1.5‐(NLG919), 1.9‐(imiquimod), 2.5‐(BMS‐1), and 3.3‐fold (BEC) higher than that of the PBS‐treated mice at 60 min post‐injection of BGR_M_. This validated that GzmB expression levels were significantly elevated upon immunotherapeutic treatment and activation of BGR_M_ by the elevated GzmB. To confirm the in situ activation of BGR_M_ in the kidneys, *ex vivo* fluorescence images of kidneys and other extra‐renal organs were recorded (Figure 4i and Figure S9). Consistent with the in vivo NIRF imaging data, fluorescence signals were seen mainly in the tumor area of resected kidneys (Figure 4i), which were 1.8‐(NLG919), 2.6‐(imiquimod), 2.9‐(BMS‐1), and 3.5‐fold (BEC) higher than that of the PBS‐treated mice (Figure 4j). Slight fluorescence signals were observed in the spleens, but no signal in other organs (Figures S9 and S10). To compare the detection ability of BGR_M_ with the clinical renal function methods, sCr and BUN in the blood of living mice were measured using the commercial assays. As shown in Figure 4g,h, the levels of sCr and BUN remained within normal ranges, suggesting renal function assays are insensitive to monitor tumor growth, not to mention the detection of immune activation.

### 2.4 BGR_M_ Activation Correlates with GzmB Levels and Differentiates Therapeutic Efficacy

**FIGURE 5:**
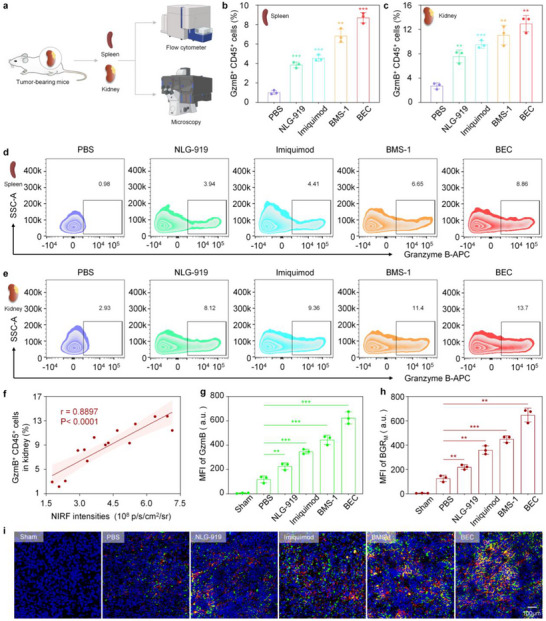
*Ex vivo* analysis of the immune response along with the treatments of immunotherapy. (a) Schematic diagram of flow cytometry and immunofluorescence staining assays for spleen and tumor‐bearing kidneys. (b,c) Quantification plots showing the GzmB levels of lymphocytes in the spleen and tumor‐bearing kidneys from mice after various treatments (n = 3, mean ± s.d.). (d,e) Representative flow cytometry plots of the GzmB levels of lymphocytes in the spleen and tumor‐bearing kidneys from mice after various treatments. (f) Correlation between the GzmB levels of lymphocytes and the in vivo NIRF signals of activated BGR_M_ via a simple linear regression model. The 95% confidence intervals were obtained by a two‐tailed Student's t‐test analysis. (g,h) Mean fluorescence intensity (MFI) of kidney sections from mice treated with different immunotherapeutics (n = 3, mean ± s.d.). (i) Representative confocal fluorescence images of regional tumor slices from immunotherapeutics‐treated mice. The blue signal is from 4′,6‐diamidino‐2‐phenylindole (DAPI); the green signal is from GzmB antibody; the red signal is from activated BGR_M_. Two‐tailed Student's t‐test; PBS versus different immunotherapeutic groups. ^**^ *p* < 0.01, ^***^ *p* < 0.001.

To validate whether the signal of BGR_M_ correlated with the expression level of GzmB, flow cytometry and immunofluorescence staining were conducted on spleen and tumor‐bearing kidneys (Figure 5a and Figures S11 and S12). As shown in Figure 5c–e, flow cytometry revealed that the GzmB expression levels of lymphocytes in tumor‐bearing kidneys from NLG919‐, Imiquimod‐, BMS‐1‐, and BEC‐treated mice were ∼2.8‐, 3.5‐, 4.1‐, and 4.8‐fold higher than that in the PBS‐treated mice, respectively. Those results were consistent with the trend of the in vivo NIRF imaging (Figure 4c–e), showing a high positive correlation between the in vivo NIRF signals of activated BGR_M_ and GzmB levels in the tumor‐bearing kidneys quantified by flow cytometry (Pearson's r = 0.8897, P<0.0001, Figure 5f). These results suggested that imaging of BGR_M_ quantitatively detected GzmB levels in vivo. Similar trends were observed for spleen tissues, showing the GzmB expression levels of lymphocytes in spleen from NLG919‐, Imiquimod‐, BMS‐1‐ and BEC‐treated mice were ∼3.7‐, ∼4.4‐, ∼6.6‐, and ∼8.4‐fold higher than that in the PBS‐treated mice, respectively (Figure 5b–d).

Moreover, immunofluorescence staining showed that the green signal from GzmB antibody staining increased in the order of Sham, PBS, NLG919, Imiquimod, BMS‐1, and BEC, which was consistent with the trend of the NIRF signals from activated BGR_M_ (Figure 5g–i). The NIRF signals of activated BGR_M_ were positively correlated with the signals of GzmB antibody staining (Pearson's r = 0.9750, P<0.0001, Figure S13), confirming the specificity of BGR_M_ for GzmB. These *ex vivo* analyses in line with the in vivo imaging results, show that the BEC‐treated group had the largest populations of GzmB‐positive leukocytes accompanied compared to other groups. To investigate the enhanced infiltration of T lymphocytes or NK cells in the tumors after different treatments, immunofluorescence staining was further conducted using a surface marker for leukocytes (CD45). As shown in Figure S14, the green signal from CD45 antibody staining increased in the order of Sham, PBS, NLG919, Imiquimod, BMS‐1, and BEC, which was consistent with the trend of the NIRF signals from activated BGR_M_, further confirming the activation of BGR_M_ correlated with the population of leukocytes. Note that these varied GzmB expression levels in the above immunotherapeutic treatment were probably due to their distinct immunoactivation mechanisms, which are not the main objective in this study. Therefore, these findings highlighted that BGR_M_ allows for real‐time in vivo imaging of immunotherapeutic response in orthotopic RCC mice.

### 2.5 BGR_M_‐Based Urinalysis for Monitoring Immunotherapy in Orthotopic RCC Mice

**FIGURE 6:**
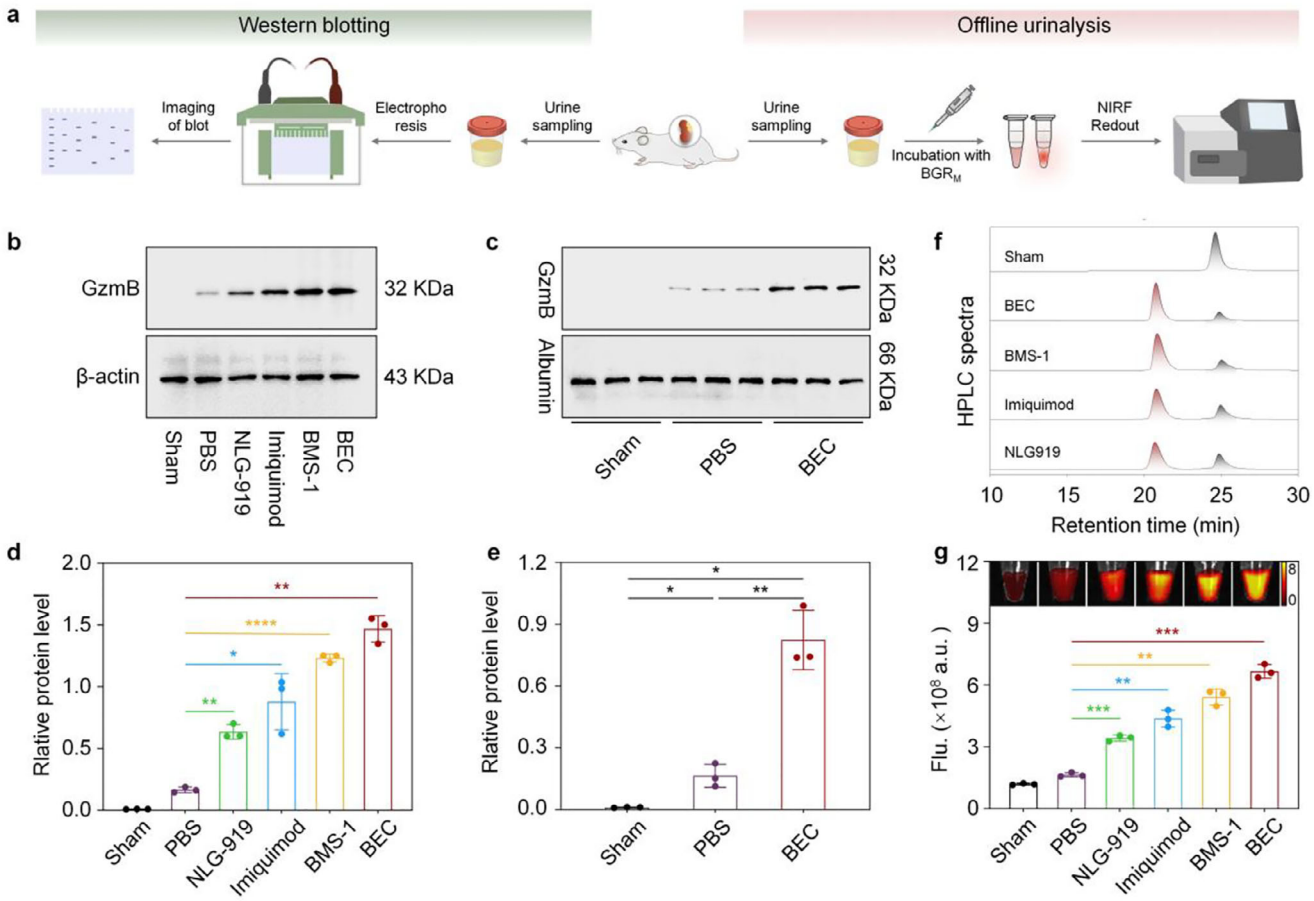
Monitoring of immunotherapy using BGR_M_‐based urinalysis. (a) Schematic illustration of the immunoblotting assay and BGR_M_‐based urinalysis for monitoring the level of urinary GzmB. (b, c) Immunoblotting of GzmB in tumor‐bearing kidneys and urine from mice treated with different immunotherapeutics. (d,e) The relative expression level of GzmB in tumor‐bearing kidneys and urine from mice treated with different immunotherapeutics (n = 3, mean ± s.d.). (f) HPLC analysis of the urine samples from living mice after incubation with BGR_M_. (g) Fluorescence enhancement of BGR_M_ after incubation with the urine samples collected from living mice treated with different immunotherapeutics (n = 3, mean ± s.d.). Two‐tailed Student's t‐test; ^*^ *p* < 0.05, ^**^ *p* < 0.01, ^***^ *p* < 0.001, ^****^ *p* < 0.0001.

Encouraged by its excellent in vivo imaging performance, we evaluated the translational potential of BGR_M_ for urinary detection of immunotherapy in orthotopic RCC mice (Figure 6a). To investigate if GzmB is a urinary biomarker of RCC immunotherapy, we performed immunoblotting analyses towards tumor‐bearing kidney tissues and urine samples. As shown in Figure 6b–d and Figures S15 and S16, immunoblotting analyses of tumor‐bearing kidney tissues revealed that GzmB was remarkably upregulated in immunotherapeutics‐treated mice, showing that NLG919‐, Imiquimod‐, BMS‐1‐, and BEC‐treated mice were ∼3.8‐, 5.3‐, 7.4‐, and 8.8‐fold higher than that in the PBS‐treated mice, respectively. Moreover, similar to that of in kidney tissues, the level of urinary GzmB was remarkably increased from BEC‐treated mice, showing ∼5.0‐fold and ∼74.6‐fold higher than the PBS‐treated group and the sham‐operation mice, respectively (Figure 6c–e). Therefore, the presence of urinary GzmB provides a biological rationale for urinalysis simply by pre‐incubating BGR_M_ with urine samples, followed by fluorescence readout (Figure 6a). Statistically significant NIRF enhancements were observed when incubating BGR_M_ with urine samples from NLG919‐, Imiquimod‐, BMS‐1‐, and BEC‐treated mice, which were ∼2.1‐, 2.7‐, 3.3‐, and 4.1‐fold higher than that in the PBS‐treated mice, respectively (Figure 6g). Correlation studies revealed that a positive correlation existed between urinary fluorescence changes and immunoblotting analyses (Pearson's r  =  0.9548, P<0.0001), in vivo NIRF imaging (Pearson's r  =  0.9804, P<0.0001), and *ex vivo* flow cytometry (Pearson's r  =  0.9443, P<0.0001) (Figure S17). To further confirm its activation in urine, the incubated urine mixture was traced using HPLC. As shown in Figure 6f, HPLC spectra identified two HPLC peaks at retention times of ∼25 min and ∼20.6 min corresponding to the prototype probe BGR_M_ and its activated product CyNA_P_‐C, respectively, signifying the conversion of BGR_M_ to CyNA_P_‐C by urinary GzmB. Therefore, BGR_M_‐based urinalysis can sensitively monitor immunoactivation in the course of immunotherapy.

### 2.6 BGR_H_‐Based Urinalysis for Monitoring Immunotherapy in RCC Patients

**FIGURE 7:**
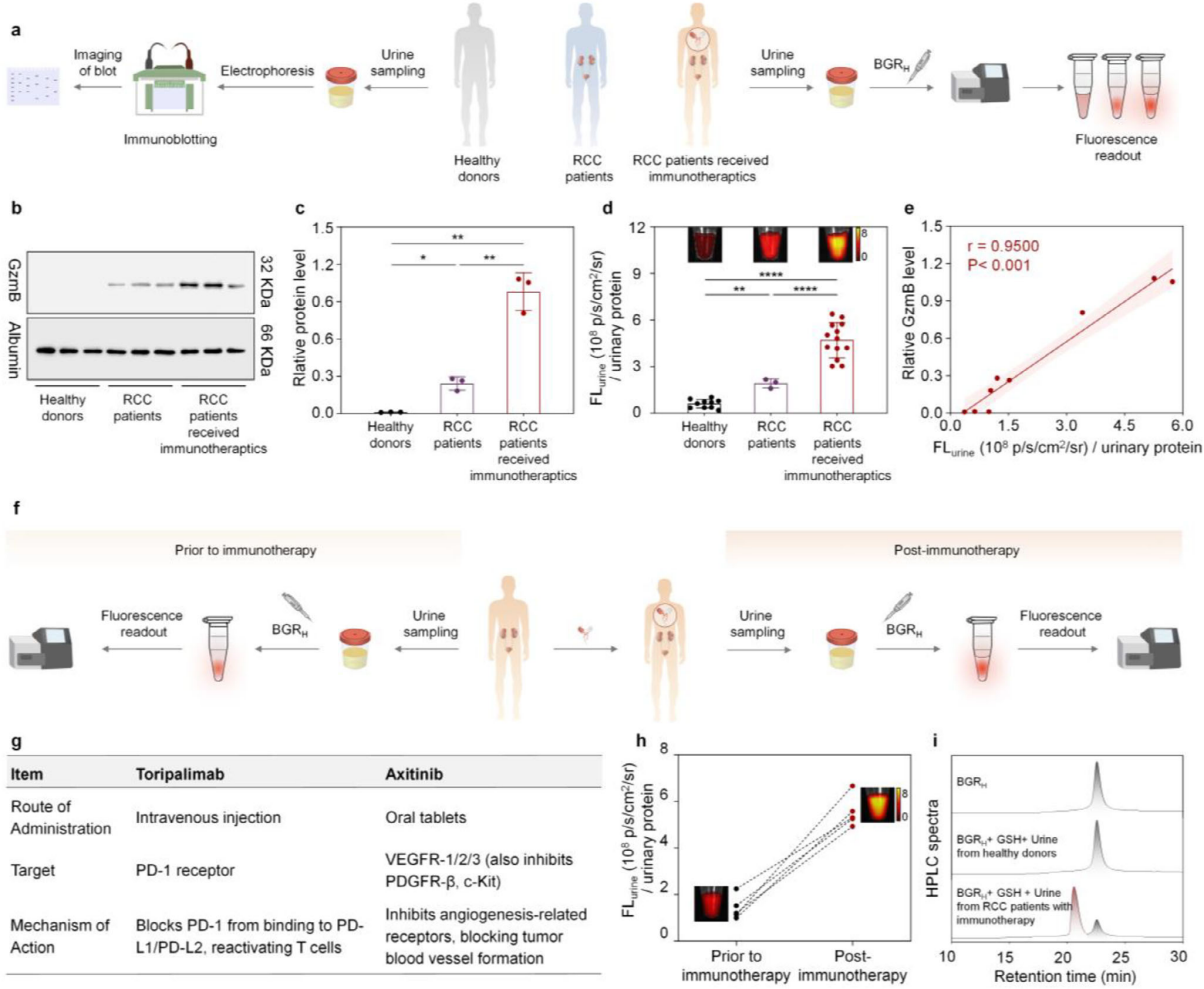
BGR_H_‐based urinalysis for monitoring immunotherapy in clinical samples. (a) Schematic illustration of the immunoblotting assay and BGR_H_‐based urinalysis for monitoring immunotherapy in a cohort of RCC patients and healthy adult volunteers. (b) Immunoblotting of GzmB in urine from RCC patients and healthy adult volunteers. (c) The relative expression level of GzmB in urine (n = 3, mean ± s.d.). (d) Fluorescence enhancement of BGR_H_ after incubation with the urine samples collected from healthy donors, RCC patients, in the absence and presence of immunotherapeutic treatment. (e) Correlation between the GzmB levels in urine by immunoblotting analysis and urinary fluorescence enhancement. The 95% confidence intervals were obtained by a two‐tailed Student's t‐test analysis. (f) Schematic illustration of monitoring immunotherapy using BGR_H_‐based urinalysis in individual patients with RCC before and after immunotherapy. (g) Table illustration of the immune checkpoint inhibitor and vascular endothelial growth factor receptor (VEGFR)‐targeting tyrosine kinase inhibitors for treatment of RCC patients. (h) Fluorescence enhancement of BGR_H_ after incubation with the urine samples collected from individual patients before and after treatment. (i) HPLC analysis of the urine samples after incubation with BGR_H_. HPLC traces of the pure compound BGR_H_ is also shown for comparison. Two‐tailed Student's t‐test; ^*^ *p* < 0.05, ^**^ *p* < 0.01, ^****^ *p* < 0.0001.

Based on the satisfactory preclinical performance of the murine GzmB‐specific probe BGR_M_, we then evaluated the translational potential of its human analog, BGR_H_ for urinary detection of immunotherapeutic response in a clinical cohort of RCC patients with biopsy‐proven and CT imaging‐proven (n = 21) and healthy adult volunteers (n = 10) (Figure 7a,f and g, and Tables S1–S2 and Figures S18 and S19). Immunoblotting analyses revealed that urinary GzmB was significantly upregulated in RCC patients (∼79.7‐fold higher than healthy donors) and remarkably increased to ∼4.0‐fold in patients undergoing immunotherapy (Figure 7b,c and Figure S20). These results affirmed GzmB as a sensitive and translational biomarker for urinary analysis of immunotherapy in RCC patients. Consistent with these findings, BGR_H_‐based urinalysis yielded approximately 3.2‐fold and 7.7‐fold NIRF enhancement in the urine from RCC patients without and with immunotherapy, respectively, relative to healthy donors (Figure 7d). Moreover, NIRF enhancement in treated patients is ∼2.4‐fold higher than in untreated patients, indicating that the GzmB expression is significantly elevated upon treatment and that BGR_H_‐based urinalysis can effectively distinguish immunoactivation. A strong positive correlation was observed between urinary fluorescence changes and immunoblotting results (Pearson's r  =  0.9500 P<0.001) (Figure 7e).

Longitudinal evaluation of immunotherapy efficacy is critical for guiding timely therapeutic interventions. We then used BGR_H_‐based urinalysis to measure urinary GzmB in individual patients with RCC before and after immunotherapy in a longitudinal follow‐up study (Figure 7f). In this cohort (n  =  5), all five patients showed a noticeable increase in urinary NIRF signals after treatment compared to baseline (Figure 7h), presumably attributed to the enhanced GzmB production in urine following immunoactivation. The activation of BGR_H_ by urinary GzmB was further confirmed via HPLC and mass spectrometry (Figure 7i; Figure S21), which revealed a new HPLC peak at a retention time of 20.6 min and a mass peak corresponding to the cyclized product CyNA_P_‐C. These results demonstrated that BGR_H_‐based urinalysis can closely track the level of immune response during immunotherapy in RCC patients.

## 3 Conclusion

Real‐time tracking of kidney‐infiltrating CTLs is essential for accurately evaluating the efficacy of immunotherapy in RCC, enabling patient stratification, reduced side effects, and timely treatment optimization. However, existing molecular probes are limited by their low kidney specificity or “always‐on” signals that poorly correlate with functional activities of immune cells. In this regard, we report the development of BGRs that can specifically detect GzmB secreted by activated CTLs through both kidney imaging and translational urinalysis for dynamic monitoring of immunotherapeutic responses in RCC.

BGRs were rationally designed with a small molecular weight (∼2.8 kDa) and high hydrophilicity (log D = −15.87 or −17.29) to minimize nonspecific protein binding and achieve excellent renal clearance efficiency (∼80% ID). When intravenously injected into a syngeneic murine RCC model with immune treatments, BGR_M_ preferentially accumulated in renal tumors, where it was cleaved by both elevated GzmB and GSH. This dual cleavage removed the highly renal‐clearable HPβCD and exposed an aminothiol site, which underwent rapid intramolecular cyclization via NAT biorthogonal click reaction, thereby triggering NIR fluorescence and spontaneous self‐assembly into nanoaggregates. This “dual cleavage‐click‐assembly” sensing mechanism enabled highly specific in vivo NIRF imaging with remarkable sensitivity comparable to flow cytometry and biopsy (Figure 5f–h) while allowing non‐invasive and dynamic tracking.

While optical imaging is generally constrained by shallow light penetration depth when it comes to clinical applications, BGRs overcome this limitation by enabling optical urinalysis of GzmB, a capability unattainable with “always‐on” signal probes (e.g., ^18^F‐FDG and ^68^Ga‐NOTA‐GZP). We observed significant upregulation of GzmB in urine samples from both RCC patients and mouse models (Figure 6c and Figure 7b), confirming its potential as a specific urinary biomarker of RCC immunotherapeutic responses. In murine RCC models, BGR_M_‐based urinalysis stratified immunoactivation under different immunotherapeutic treatments, showing a strong positive correlation with immunoblotting results (Figure S17). In human urine specimens, BGR_H_‐based urinalysis not only revealed subtle GzmB fluctuations between healthy volunteers and RCC patients prior to immunotherapeutic treatment (Figure 7d), but also enabled longitudinal monitoring of immune responses post‐treatment (Figure 7h). With high sensitivity and specificity for urinalysis, these probes address a long‐standing clinical need that remains unattainable using radiolabeled antibody or peptide.

In conclusion, this work presents a novel class of renal‐clearable bio‐orthogonal NIR fluorogenic probes for both real‐time imaging of kidney‐infiltrating CTLs and in vitro urinalysis of GzmB. These probes demonstrate strong translational potential and highlight a significant advance in addressing the critical gap in clinical monitoring of RCC immunotherapy, thereby supporting timely patient stratification and treatment planning. Furthermore, this study establishes a foundational design principle for transforming bio‐orthogonal probes into urinary reporters, which can be readily adapted for evaluating immunotherapeutic responses in other urological cancers, such as prostate and bladder cancer.

## 4 Experimental Section

### 4.1 TEM Assay

1 µM BGR_M_ was incubated with activated GzmB enzyme and GSH for 24 h in phosphate‐buffered solution (1×PBS, pH = 7.4). The carbon‐coated side of the grid was gently immersed in the dispersion. The photo was taken by a Hitachi TEM system. Magnification = 40 000, Accelerating Voltage = 100 000.

### 4.2 Optical Measurement

BGR_M_ (20 µM) was incubated with activated murine GzmB at 37 °C for 6 h and then incubated with GSH for 12 h. BGR_M_ (20 µM) in PBS was set as the control. After incubation, fluorescence spectra were measured. Noted that GzmB (1 µg) was activated by Cathepsin C (0.4 µg) in MES buffer (50 mM MES, 50 mM NaCl, pH 5.5) for 4 h. The response of BGR_H_ towards human GzmB was conducted according to the above steps.

### 4.3 In Vitro Stability and Selectivity Studies

Fluorescence intensity of BGR_M_ was measured after incubation in PBS and FBS for 0, 6, 12, and 24 h. BGR_M_ was incubated with the indicated enzymes (0.5 µg), metal ions (60 µM), or ROS (60 µM) in MES buffer (50 mM MES, 50 mM NaCl, pH 5.5) at 37 °C. •OH was generated by the Fenton reaction between H_2_O_2_ and Fe(ClO_4_)_2_; Hydrogen peroxide (H_2_O_2_); Gamma‐Glutamyl Transferase (GGT); NAG, N‐acetyl‐beta‐β‐glucosaminidase, Cathepsin B, Cathepsin D, Cathepsin K, Caspase 8, Caspase 3, Glutathione (GSH); Granzyme B (GzmB). Fluorescence enhancement was measured on a fluorescence spectrophotometer after incubation.

### 4.4 Isolation and Activation of Mouse CD8^+^ T Cells

Primary CD8^+^ T cells were isolated from the spleens of Balb/c mice using the EasySep Mouse CD8^+^ T Cell Isolation Kit via immunomagnetic negative selection according to the manufacturer's protocol. To induce effector differentiation, the isolated CD8^+^ T cells were cultured in RPMI 1640 medium supplemented with 10% FBS and stimulated with anti‐mouse CD3e (2 µg mL^−1^) and anti‐mouse CD28 (5 µg mL^−1^) for 48 h. For T cell reinvigoration and to ensure peak expression of active granzyme B, cells were further incubated with recombinant murine IL‐2 (80 U mL^−1^) for 3 h prior to experiments. The resulting effector CD8^+^ T cells were subsequently collected and utilized for in vitro co‐culture assays with Renca tumor cells.

### 4.5 Cell Culture

Renca cells were cultured in RPMI‐1640 medium supplemented with 10% FBS and 1% penicillin‐streptomycin. For mouse CD8^+^ T cells, the RPMI‐1640 medium was further supplemented with 50 µM β‐mercaptoethanol and 80 U/mL mouse recombinant IL‐2 (rIL‐2) to maintain cell viability and effector function. All cells were incubated at 37 °C with 5% CO_2_. For co‐culture imaging, Renca cells (1 × 10^5^ cells/dish) were seeded in glass‐bottom dishes and allowed to adhere overnight. Activated CD8^+^ T cells were then added to the Renca cells at an effector‐to‐target (E:T) ratio of 5:1. The co‐culture was incubated for 5 h prior to confocal fluorescence imaging.

### 4.6 Confocal Fluorescence Imaging

Cellular fluorescence images were acquired using an Olympus FV3000 confocal laser scanning microscope. Images were captured using a 40× objective with an optical zoom of 3.0 to provide a detailed visualization of cell‐cell interactions. For multi‐color imaging, DAPI (cell nuclei), Alexa Fluor 488 (CD45‐labeled CD8^+^ T cells), and the BGR_M_ probe were excited at 405, 488, and 561 nm, respectively. The pinhole was maintained at 1.0 Airy unit. All images were captured at a resolution of 1024 × 1024 pixels using Olympus CellSens software. Consistent acquisition parameters (laser power, HV gain, and offset) were used across all experimental groups for quantitative comparison.

### 4.7 In Vivo Biodistribution and Biocompatibility

All animal studies were conducted in accordance with the guidelines set by the Institutional Animal Care and Use Committee (IACUC), Sun Yat‐sen University (SYSU), with the approval number SYSU‐IACUC‐2025‐002114. To determine the clearance pathway and biocompatibility in living mice, Male Balb/c mice (6‐8 weeks old) were injected with BGR_M_ intravenously (10 µmol kg^−1^ body weight). All mice were fed with non‐fluorescent chow (Xietong Pharmaceutical Bioengineering CO., Ltd, XT19008) and kept in a SPF environment. Mice in both the experimental and control groups were randomly assigned. NIRF imaging of resected major organs (heart, liver, spleen, lung, kidneys, bladder, muscle, skin, and intestine) was performed and analyzed by IVIS Lumina XR Series III (Perkin Elmer Inc, USA) at 24 h post‐injection. The major organs were collected and placed into 4% paraformaldehyde (4% PFA) at 4 °C for histological examination.

### 4.8 Pharmacokinetic Studies

Healthy mice were anesthetized for the entire duration of the experiment. The end of the tail was cut for blood extraction. Blood was sampled in heparinized capillary tubes as a reference before injection. Mice were intravenously injected with BGR_M,_ and blood was sampled at 1, 4, 9, 16, 25, 35, 55, 75, 95, 120, 240, 720, 1440 min post‐injection. Collected blood samples were stored in an ice box to prevent clotting before centrifugation at 3500 r.p.m for 20 min. BGR_M_ was traced using HPLC quantification. Quantification results were presented as a bi‐exponential decay curve to estimate elimination (t_1/2β_) blood half‐life values.

### 4.9 Establishment of a Mouse Model of Orthotopic RCC

All experimental procedures involving tumor‐bearing models strictly comply with internationally recognized ethical guidelines. In compliance with research ethics requirements, the tumor size in laboratory mice must remain below 2000 mm^3^ during experimentation. Male Balb/c mice (6‐8 weeks old) were randomly selected and utilized for Renca‐Luc cells implantation. Renca‐Luc cells were maintained in culture, combined with Matrigel and PBS in equal volumes (1:1), and kept briefly on ice prior to use. Anesthesia in Balb/c mice was maintained via a precision isoflurane anesthesia machine (1.5‐2.5% in oxygen carrier gas). Apply pressure to the foot and assess reflex response to ensure sufficient anesthetization. Use a vet ointment to prevent dryness in the eyes. Anesthetized Balb/c mice were placed on the surgical table and shaved on the right side between the fore‐ and hind‐limb. A 1 cm skin incision was made longitudinally between the last rib and the hip joint using surgical scissors after alcohol and iodine application. After kidney exposure, ∼2 ( 10^4^ Renca‐Luc cells were transplanted beneath the renal capsule via needle insertion until a small white bubble forms. Slowly remove the needle from the capsule to prevent cell dissemination. The muscle layer and the skin were sutured with silk sutures. The mice were returned to the SPF environment for further rearing with non‐fluorescent chow.

### 4.10 Bioluminescence Imaging

To monitor tumor growth, in vivo bioluminescence imaging was performed at 5 days post tumor implantation by intraperitoneal (IP) injection of D‐luciferin salt solution (10 µL g^−1^). At 10 min post‐injection, bioluminescence imaging was conducted using the IVIS Spectrum. Regions of interest (ROI) were drawn around whole mice to calculate average radiance (p/s/cm^2^/sr) within the region.

### 4.11 Real‐Time NIRF Imaging of Immunotherapeutic Response in Orthotopic RCC Mice

The above tumor‐bearing Balb/c mice were randomly grouped and treated with different immunotherapeutic agents. At 6‐, 7‐, and 8‐days post tumor implantation, immunotherapeutics including NLG919 (20 mg kg^−1^ body weight), Imiquimod (20 mg kg^−1^ body weight), BMS‐1 (20 mg kg^−1^ body weight), and BEC (20 mg kg^−1^ body weight) were intravenously administered into living mice once a day for 3 days. The control groups, including the sham‐operation group and tumor‐bearing mice, were treated with PBS (0.2 mL) once a day for 3 days. At 9 days post‐tumor implantation, BGR_M_ was intravenously injected. Real‐time NIRF imaging was conducted at t = 0‐, 30‐, 60‐, 90‐, 120‐, or 150‐min post‐injection of BGR_M_ with an acquisition time of 3.0 s (excitation at 580 ± 10 nm and emission at 670 ± 10 nm). Besides, Blood and urine samples from each group were collected for biochemical analysis. The NIRF imaging of resected major organs (heart, liver, spleen, lung, kidneys, bladder, muscle, skin, and intestine) was performed and analyzed by IVIS Lumina XR Series III. The major organs were collected and frozen in liquid nitrogen for histological examination and immunofluorescence assays.

### 4.12 Patients

A cohort of 21 RCC patients was enrolled at the Department of Urology, the first affiliated hospital of Sun Yat‐sen University, Guangzhou, China. All the included patients signed an informed consent prior to inclusion in the study. The demographic characteristics of the cohorts are depicted in Table S1–S2. Representative computed tomography (CT) scans and hematoxylin–eosin (H&E)‐stained pathology images were obtained solely for research and illustrative purposes, with all patient identifiers removed to ensure anonymity. The study was approved by the Institutional Ethics Committee of The First Affiliated Hospital of Sun Yat‐sen University (Approval No. [2026]241).

### 4.13 Urinalysis

Urinalysis was performed in both mouse and human samples. For mice, urine was collected using metabolic cages, while human urine samples were obtained from healthy donors, RCC patients, and RCC patients receiving therapy (Axitinib plus Toripalimab). All collected urine samples were centrifuged at 4,000 r.p.m. for 10 min, and the supernatant was filtered through a 0.22 µm syringe filter. Subsequently, BGR_M_ (mice) or BGR_H_ (human) solutions (10 µM) with GSH in PBS (10 mM, pH 7.4) were incubated with urine aliquots (100 µL) at 37 °C for 3 h, followed by fluorescence imaging using an IVIS Spectrum system and quantitative analysis (Excitation at 550 ± 10 nm and Emission at 675 ± 10 nm; acquisition time, 3 s). Urinary proteins were also determined, and the NIRF signals in urine were normalized to the concentration of urinary proteins.

### 4.14 Western Blot Assay

Urine and kidney tissue samples from mice, as well as urine samples from RCC patients, were subjected to western blot (WB) analysis. Concentrated urine samples were diluted in sample buffer and boiled at 100 °C for 6 min, followed by sodium dodecyl sulfate–polyacrylamide gel electrophoresis (SDS‐PAGE) for 90 min and transfer onto polyvinylidene difluoride (PVDF) membranes for 2 h. The membranes were closed for 1 h at rt in 5% skimmed milk solution (Tris‐buffered saline, 0.1% TBS‐T, 5% skimmed powdered milk). and then incubated with anti‐ granzyme B (Cat No. 701395, dilution 2 µg mL^−1^) antibody, β‐actin (Cat No. 66009‐1‐Ig, dilution 1:20000), Albumin antibody (Cat No. 16475‐1‐AP, dilution 1:5000) at 4 °C overnight, followed by incubation of the secondary antibody for 120 min at room temperature. Immunoreactive bands were incubated with the Enhanced Chemiluminescent Protein Blotting Kit (Sigma, USA) and visualized using the ChemiDoc MP Imaging System. Density of the bands was measured using ImageJ software and normalized to healthy controls.

### 4.15 Flow Cytometric Assay

For the analysis of GzmB level in leucocytes in mice, the spleen and kidney were harvested from mice at different times and prepared as single‐cell suspensions. Briefly, the kidney was minced, gently ground in ice‐cold 1× PBS, and filtered through a 70 µm strainer. Red blood cells in the collected cell suspension were removed using eBioscience 1× RBC Lysis Buffer (Invitrogen). The immune cells in the kidney were then isolated by density gradient centrifugation of a single‐cell suspension with 40% and 70% Percoll (850 g, 20 min), followed by washing with ice‐cold 1× PBS. Spleen was gently ground in ice‐cold 1× PBS with a syringe plunger and filtered through a 70 µm cell strainer to afford a single‐cell suspension. Red blood cells in a single‐cell suspension of spleen were removed with eBioscience 1× RBC Lysis Buffer (Invitrogen). Immune cells in blood were isolated by density gradient centrifugation with Histopaque 1077 (400 g, 30 min). After single‐cell suspension, the collected cells from spleen and kidney were blocked with anti‐mouse CD16/32 and stained with APC/Fire 750 anti‐mouse CD45, LIVE/DEAD Fixable Dead Cell Stain Kits, and APC anti‐human/mouse Granzyme B according to the vendors’ protocols. The stained cells were analyzed using flow cytometry.

### 4.16 Histology

The collected tissues were fixed with 4% paraformaldehyde (PFA) or rapidly frozen at low temperatures. After routine dehydration, paraffin‐embedded tissues were embedded in paraffin and sectioned (10 µm of thickness) for H&E staining (hematoxylin and eosin staining) according to the instructions. The stained sections were observed under the EVOS M7000 microscope. Frozen sections were fixed on 100% acetone under −20 °C for 20 min and blocked with 10% goat serum for 90 min at room temperature. Followed with anti‐Granzyme B (dilution 2 µg mL^−1^) as primary antibody incubated overnight at 4 °C, goat anti‐rabbit IgG − Alexa Fluor 488 (dilution 1:200) as second antibody incubated for 1 h at room temperature, followed by DAPI staining for 5 min and sealing with sealing agent. Immunofluorescence experiments were washed with PBS 3 times for 5 min each step. Fluorescence signal distribution and intensity were observed using an FV3000 laser confocal microscope (Olympus) after staining.

### 4.17 Statistical Analysis

The in vitro, in vivo, and ex vivo fluorescence signals were quantified with region of interest analysis (ROI) and analyzed by Living Image software (version 4.4). All the results were mean ± standard deviation (SD) unless stated otherwise. All investigators were blinded to group allocation during experiments. Statistical differences between two groups were tested with a two‐tailed Student's t test, and more than three groups were determined by one‐way analysis of variance followed by Tukey's post hoc test. All statistical calculations were performed using GraphPad Prism 8.0 and OriginPro 2024, including assumptions of tests used. For all tests, P values less than 0.05 were considered statistically significant (^*^ *p* < 0.05, ^**^ *p* < 0.01, ^***^ *p* < 0.001, and ^****^ *p* < 0.0001).

## Conflicts of Interest

The authors declare no conflicts of interest.

## Supporting information




**Supporting File**: advs75429‐sup‐0001‐SuppMat.docx.

## Data Availability

The data that support the findings of this study are available from the corresponding author upon reasonable request.
