# Supplementary material for: Bioorthogonal Fluorogenic Reporters for Noninvasive Imaging and Urinalysis of Immunotherapeutic Response in Renal Cell Carcinoma
Source: Adv Sci (Weinh). 2026 Apr 24;13(40):e24298. doi: 10.1002/advs.202524298 (PMC13335466; doi:10.1002/advs.202524298)
Supplement: Supplementary file 1 — Supporting File: advs75429‐sup‐0001‐SuppMat.docx. [file ADVS-13-e24298-s001.docx]

Supporting Information

**Bioorthogonal Fluorogenic Reporters for Noninvasive Imaging and Urinalysis of Immunotherapeutic Response in Renal Cell Carcinoma**

*Xingyue Yang*†*, Baoshuai Liang*†*, Liangmin Fu*†*,Weiliang Deng, Yuyan Jiang, Weiping Xu, Jinwei Chen, Ya Zhou*, Ke Wu*, Jiaguo Huang**

X. Yang, B. Liang, W. Deng, W. Xu, J. Huang
State Key Laboratory of Anti-Infective Drug Discovery and Development, School of Pharmaceutical Sciences, Sun Yat-sen University, Guangzhou, China
E-mail: [huangjg36@mail.sysu.edu.cn](mailto:huangjg36@mail.sysu.edu.cn)

Y. Zhou, K. Wu

Department of Pharmacy, The Affiliated Dazu's Hospital of Chongqing Medical University, Chongqing, China

E-mail: [wuke@cqmu.edu.cn](mailto:wuke@cqmu.edu.cn); [zhouya@hospital.cqmu.edu.cn](mailto:zhouya@hospital.cqmu.edu.cn)

L. Fu
Department of Urology, The Second Xiangya Hospital of Central South University, Changsha, China

L. Fu, J. Chen
Department of Urology, The First Affiliated Hospital of Sun Yat-sen University. Guangzhou, China

Y, Jiang
Department of Radiation Oncology, Stanford University School of Medicine, Stanford, CA, USA

† These authors contributed equally to this work.

**1. Materials and Methods**

**Materials characterization.** Proton-nuclear magnetic resonance (^1^H NMR) was conducted with a Bruker 400 MHz NMR instrument. Data were reported as follows: chemical shift, integration, multiplicity (s = singlet, d = doublet, t = triplet, q = quartet, m = multiplet, br = broad), coupling constants (Hz), and assignment. Preparative HPLC was performed on a Shimadzu LC-16P system equipped with an Ascentis®C18 HPLC Column (250 × 21.2 mm, 5 μm) using gradient elution. The analytical HPLC was performed on a dual pump Shimadzu LC-16 system equipped with an Ascentis® C18 HPLC column (4.6 × 250 mm, 5 μm) using methanol/water (containing 0.1% Formic acid) as the mobile phase at a flow rate of 1 mL/min. A silicone oil bath was used for experiments requiring heating and reaction products were purified by column chromatography on silica gel (Qingdao Haiyang Chemical, 200-300 mesh). UV-vis spectra were recorded on a Shimadzu UV-2600 spectrophotometer using quartz cuvettes (1 cm path length). Fluorescence spectra were measured on HORIBA Fluoromax-4 fluorescence spectrophotometer using the same cuvettes. Confocal fluorescence microscopy images were acquired on a FV3000 Laser scanning ultrahigh resolution microscope (Olympus, Japan). Fluorescence imaging was performed on the IVIS Lumina XR Series III (PerkinElmer, Inc, USA) for mice and urine samples. Intelligent imaging system of EVOS M7000 (Thermo Fisher Scientific) was used for histopathological examination. Multimode microplate reader (Synergy H1 of Bio-Tek) was used for measurement of absorbance and fluorescence intensity. ChemiDoc MP Imaging System of Bio-Rad was used for protein detection.

**Chemicals and Biological reagent.** All chemicals were from commercial companies (Sigma-Aldrich, GL Biochem, Bide Pharmatech, Aladdin Bio-Chem Technology or Macklin Biochemical) unless otherwise stated. Granzyme B Protein, Human (Cat No. HY-P703940), Granzyme B Protein, Mouse (Cat No. HY-P701245), Cathepsin C/DPPI Protein, Mouse (Inactive, His-Myc) (Cat No. P97821), BEC (CAS: 222638-67-7), BMS-1 (CAS: 1675201-83-8), Recombinant murine IL-2 (Cat No. HY-P7077, 80 U mL^-1^) were from MedChemExpress (MCE). NLG919 (CAS: 1402836-58-1) was from DC chemicals. Imiquimod (CAS: 99011-02-6) was from MedKoo Biosciences. Cathepsin B Protein, Mouse (Cat No. AB219240), Cathepsin D Protein, Mouse (Cat No. AB219240), Secondary antibody goat anti-rabbit IgG H&L (Alexa Fluor® 488) (Cat No. ab150077, dilution 1:200) were from Abcam. Cathepsin K Protein, Mouse (Cat No. TMPH-02565) was from Targetmol. Caspase 8 Protein, Mouse (Cat No. 1185-25) was from Biovision. Caspase 3 Protein, Mouse (Cat No. CSB-EP004548MO) was from CUSABIO. β-actin antibody (Cat No. 66009-1-Ig, dilution 1:20000), Albumin antibody (Cat No. 16475-1-AP, dilution 1:5000), CD45 antibody (Cat No. 20103-1-AP, dilution 1:200) were from Proteintech. Granzyme B Antibody (23H8L20) (Cat No. 701395, 2 μg mL^-1^), β-mercaptoethanol (Cat No. 31350010) and LIVE/DEAD™ Fixable Dead Cell Stain Kits (Cat No. L34969) were from Thermo Fisher scientific. Goat Anti-Mouse IgG Secondary Antibody HRP Conjugated (Cat No. L3032) and Goat Anti-Rabbit IgG Secondary Antibody HRP Conjugated (Cat No. L3012) were from Signalway Antibody (SAB). CD16/32 antibody (Cat No. 156603), APC/Fire™ 750 CD45 antibody (Cat No. 147713), APC Granzyme B antibody (Cat No. 372203) were from Biolegend. Dialysis Membranes (Cat No. SP131048-1m) was from Shanghai Yuanye Biotechnology. BCA protein concentration determination kit (Cat No. P0011) and PMSF (100mM) (Cat No. ST506) were from Beyotime. EasySep™ Mouse CD8^+^ T Cell Isolation Kit (Cat No. 19853) was from STEMCELL Technologies. Anti-mouse CD3e (Cat No. 16-0031-85, 2μg mL^-1^) and anti-mouse CD28 (Cat No. 16-0281-82, 5μg mL^-1^) were from Invitrogen.

**Synthesis of compound 1.** To a solution of 1,2-bis(pyridin-2-yl)disulfide (33 g, 150 mmol) in methanol (250 mL) was slowly added 2-aminoethanethiol hydrochloride (5.68 g, 50 mmol) pre-dissolved in methanol (100 mL) dropwise at 0 °C and stirred for 12 h at room temperature. After completion of the reaction, the solvent was removed under reduced pressure. The resulted residue was precipitated in ethyl acetate. The mixture was filtered under reduced pressure and the resulted crude product was re-dissolved with methanol and re-precipitated in ethyl acetate. The mixture was filtered and washed with ethyl acetate and dried to give a white solid (9.83 g, 89% yield). ^1^H NMR (400 MHz, Deuterium Oxide) δ 8.50 (d, *J* = 5.0 Hz, 1H), 7.88 (t, *J* = 7.9 Hz, 1H), 7.79 (d, *J* = 8.1 Hz, 1H), 7.38 (t, *J* = 6.1 Hz, 1H), 3.41 (t, *J* = 6.3 Hz, 2H), 3.17 (t, *J* = 6.3 Hz, 2H). ESI-MS (m/z): calcd for C_7_H_10_N_2_S_2_ [M+H]^+^: 187.03; found: 187.03.

**Synthesis of** **compound 2.** To a solution of ethyl 2-azidoacetate (3.87 g, 30 mmol) in 150 mL methanol /water=1:1) was added LiOH (2.16 g, 90 mmol) and stirred for 1 h at room temperature. After completion of the reaction, the mixture was poured into ethyl acetate (150 mL) and 1 M hydrogen chloride (150 mL) for stratification. The organic phase was collected and the aqueous phase was extracted 5 times with ethyl acetate. All organic phases were combined and dried with anhydrous Na_2_SO_4_, filtered and the filtrate was concentrated under reduced pressure to obtain a colorless oily liquid. The crude product was directly used to the next step without need further purification.

**Synthesis of compound 3.** To a solution of compound **2** (2.0 g, 20 mmol) in dichloromethane (100 mL) was added N-Hydroxy succinimide (2.76 g, 24 mmol) and dicyclohexylcarbodiimide (4.95 g, 24 mmol), and stirred at room temperature. After 4 h, the precipitate was filtered off and the filtrate was evaporated to dryness. The resulted residue was redissolved with ethyl acetate and the filtrate was washed twice with saturated NaHCO_3_ solution, once with brine and dried with anhydrous Na_2_SO_4_. The obtained filtrate was concentrated under reduced pressure to give a crude product, which was recrystallized with ethyl acetate / petroleum ether to afford a white solid (1.8 g, 45% yield). ^1^H NMR (400 MHz, Chloroform-*d*) δ 4.24 (s, 2H), 2.88 (s, 4H).

**Synthesis of compound 4.** To a solution of compound **1** (1.23 g, 5.56 mmol) and compound **3** (1.0 g, 5.05 mmol) in N,N-dimethylformamidex (50 mL) was added tethylamine (2.11 mL, 15.2 mmol), and stirred at room temperature. After 18 h, the solvent was removed under reduced pressure. The residue was extracted 3 times with dichloromethane and the resulted organic phases were washed 3 times with brine and dried with anhydrous Na_2_SO_4_. The filtrate was concentrated under reduced pressure and purified by silica gel column chromatography (petroleum ether / ethylacetate = 1:1, v/v) to afford a colorless viscous liquid (630 mg, 46% yield). ^1^H NMR (400 MHz, Chloroform-*d*) δ 8.57 (d, *J* = 4.9 Hz, 1H), 8.09 (s, 1H), 7.61 (td, *J* = 7.7, 1.8 Hz, 1H), 7.48 (d, *J* = 8.1 Hz, 1H), 7.20-7.12 (m, 1H), 4.01 (s, 2H), 3.60 (q, *J* = 6.0 Hz, 2H), 2.91 (t, *J* = 5.9 Hz, 2H). ESI-MS (m/z): calcd for C_9_H_11_N_5_OS_2_ [M+Na]^+^: 292.04; found: 292.23.

Compound **CyNA_P_** was synthesized according to our previous study.^1^

**Synthesis of compound CyNA_P_N_3_.** The compound **CyNA_P_** (44 mg, 0.059 mmol) and **4** (19 mg, 0.071 mmol) were dissolved in methanol (15 mL) and stirred at room temperature. After 12 h, the solvent was removed under reduced pressure. The residue was purified using HPLC to obtain a purple solid (36 mg, 68% yield). ^1^H NMR (500 MHz, Methanol-*d*_4_) δ 8.76 (d, *J* = 15.4 Hz, 1H), 7.77 (s, 1H), 7.73 (t, *J* = 8.2 Hz, 2H), 7.61-7.54 (m, 4H), 7.20 (s, 1H), 6.92 (d, *J* = 15.6 Hz, 1H), 4.73 (t, *J* = 5.0 Hz, 2H), 4.01 (t, *J* = 4.9 Hz, 2H), 3.90 (s, 2H), 3.61-3.58 (m, 2H), 3.52-3.49 (m, 2H), 3.42 (t, *J* = 5.4 Hz, 2H), 2.99 (dd, *J* = 14.4, 7.9 Hz, 1H), 2.89 (t, *J* = 6.6 Hz, 2H), 2.80 (t, *J* = 6.2 Hz, 2H), 2.73 (t, *J* = 6.1 Hz, 2H), 1.95 (q, *J* = 6.2 Hz, 2H), 1.87 (s, 6H). ESI-MS (m/z): calcd for C_39_H_47_N_8_O_5_S_2_^+^ [M]^+^: 771.31; found: 771.50.

**Synthesis of compound IEFD.** Compound **IEFD** was prepared using solid phase peptide synthesis to give a white powder. ^1^H NMR (500 MHz, Methanol-*d*_4_) δ 8.11 (dd, *J* = 49.1, 7.7 Hz, 1H), 7.24 (d, *J* = 4.3 Hz, 4H), 7.17 (h, *J* = 4.2 Hz, 1H), 4.74 (t, *J* = 6.3 Hz, 1H), 4.68-4.61 (m, 1H), 4.34-4.25 (m, 1H), 4.13 (d, *J* = 7.3 Hz, 1H), 3.20 (dd, *J* = 14.0, 5.1 Hz, 1H), 2.94 (dd, *J* = 14.0, 9.2 Hz, 1H), 2.79 (dd, *J* = 16.4, 5.8 Hz, 1H), 2.70 (dd, *J* = 16.3, 6.9 Hz, 1H), 2.27-2.18 (m, 1H), 2.18-2.10 (m, 1H), 2.00 (s, 3H), 1.98-1.91 (m, 1H), 1.84-1.75 (m, 2H), 1.44 (s, 18H), 1.22-1.13 (m, 1H), 0.90 (t, *J* = 7.4 Hz, 3H), 0.85 (d, *J* = 6.8 Hz, 3H). ESI-MS (m/z): calcd for C_34_H_52_N_4_O_10_ [M+Na]^+^: 699.37; found: 699.53.

**Synthesis of compound CyNA_P_N_3_IEFD-tBu.** To a solution of compound **CyNA_P_N_3_** (39 mg, 0.043 mmol) and compound **IEFD** (29 mg, 0.043 mmol) in dichloromethane (25 mL) was added O-Benzotriazole-N,N,N',N'-tetramethyl-uronium-hexaf (18 mg, 0.047 mmol) and N,N-diisopropylethylamine (15 µL, 0.086 mmol), and stirred at room temperature. After 3 h, the solvent was removed under reduced pressure. The residue was purified using HPLC to obtain a purple solid (54 mg, 81% yield). ^1^H NMR (400 MHz, Methanol-*d*_4_) δ 8.75 (d, *J* = 15.5 Hz, 1H), 7.76 (s, 1H), 7.71 (t, *J* = 6.9 Hz, 2H), 7.60-7.52 (m, 4H), 7.25-7.23 (m, 4H), 7.18 (d, *J* = 6.6 Hz, 2H), 6.93 (d, *J* = 15.5 Hz, 1H), 4.71 (t, *J* = 5.1 Hz, 2H), 4.63 (dd, *J* = 8.4, 5.5 Hz, 2H), 4.56 (d, *J* = 4.6 Hz, 1H), 4.48 (dd, *J* = 10.3, 4.8 Hz, 1H), 4.14 (dd, *J* = 8.5, 5.2 Hz, 1H), 4.06 (d, *J* = 6.2 Hz, 1H), 4.00 (t, *J* = 5.0 Hz, 2H), 3.87 (s, 2H), 3.58-3.55 (m, 2H), 3.50-3.49 (m, 2H), 3.42 (t, *J* = 6.0 Hz, 2H), 3.17 (dd, *J* = 10.6, 4.7 Hz, 2H), 3.00 (dd, *J* = 14.2, 10.3 Hz, 2H), 2.83-2.78 (m, 4H), 2.74-2.71 (m, 2H), 2.07 (s, 2H), 1.94 (t, *J* = 6.0 Hz, 2H), 1.86 (s, 6H), 1.79 (t, *J* = 6.8 Hz, 2H), 1.43 (s, 18H), 0.94-0.90 (m, 6H). ESI-MS (m/z): calcd for C_73_H_97_N_12_O_14_S_2_^+^ [M]^+^: 1429.67; found: 1429.92.

**Synthesis of compound CyNA_P_N_3_IEFD.** A solution of compound **CyNA_P_N_3_IEFD-tBu** (47 mg, 0.03 mmol) in 10 mL dichloromethane / trifluoroacetic acid (4:1, v/v) was stirred at room temperature. After 5 h, the solution was concentrated under reduced pressure. The residue was purified using HPLC to obtain a purple solid (21 mg, 49% yield). ^1^H NMR (400 MHz, Methanol-*d*_4_) δ 8.75 (d, *J* = 15.5 Hz, 1H), 7.75 (s, 1H), 7.71 (t, *J* = 6.7 Hz, 2H), 7.61-7.52 (m, 4H), 7.26-7.21 (m, 4H), 7.17 (s, 2H), 6.92 (d, *J* = 15.5 Hz, 1H), 4.49-4.47 (m, 1H), 4.20-4.15 (m, 1H), 4.07 (d, *J* = 6.5 Hz, 1H), 4.01 (t, *J* = 4.7 Hz, 2H), 3.88 (s, 2H), 3.59-3.55 (m, 2H), 3.52-3.47 (m, 4H), 3.42 (t, *J* = 5.8 Hz, 2H), 3.23-3.14 (m, 3H), 3.00-2.91 (m, 2H), 2.83-2.78 (m, 4H), 2.72 (t, *J* = 6.2 Hz, 2H), 2.66 (s, 3H), 2.06 (s, 2H), 2.03 (d, *J* = 8.5 Hz, 1H), 1.94 (q, *J* = 5.8 Hz, 2H), 1.86 (s, 6H), 0.93-0.88 (m, 6H). HRMS (m/z): calcd for C_65_H_81_N_12_O_14_S_2_^+^ [M]^+^: 1317.5431; found: 1317.5438.

Compound **Propynyl-HPβCD** was synthesized according to our previous study.^2^

**Synthesis of probe BGR_M_.** To a solution of compound **CyNA_P_N_3_IEFD** (20 mg, 0.014 mmol) and **Propynyl-HPβCD** (34 mg, 0.021 mmol) in DMSO (3 mL) was added CuSO_4_**·**5H_2_O (1 mg, 0.004 mmol) and sodium ascorbate (1 mg, 0.004 mmol). The reaction mixture was stirred under argon atmosphere at room temperature. After 12 h, the mixture was diluted with water. Purified by dialysis (membrane with MW cutoff: 1500) and lyophilized to give a purple powder (33 mg, 79% yield). ^1^H NMR (500 MHz, Methanol-*d*_4_) δ 8.74 (d, *J* = 15.5 Hz, 1H), 8.10 (s, 1H), 7.76-7.69 (m, 3H), 7.59-7.52 (m, 4H), 7.27-7.21 (m, 6H), 6.98 (d, *J* = 7.1 Hz, 1H), 5.48-5.45 (m, 6H), 4.25-4.19 (m, 12H), 3.85-3.80 (m, 82H), 3.55-3.53 (m, 24H), 2.51 (t, *J* = 6.5 Hz, 2H), 2.46 (t, *J* = 11.8 Hz, 2H), 2.19 (t, *J* = 8.0 Hz, 2H), 2.04-2.00 (m, 3H), 1.94-1.91 (m, 2H), 1.85 (s, 6H), 1.17-1.15 (m, 24H), 0.90-0.88 (m, 6H). MALDI-TOF MS: found: 2500-3200.

**Synthesis of compound IEPD.** Compound **IEPD** was prepared using solid phase peptide synthesis to give a white powder. ^1^H NMR (400 MHz, Methanol-*d*_4_) δ 8.27-7.99 (m, 2H), 4.77-4.69 (m, 2H), 4.46 (dd, *J* = 8.6, 3.5 Hz, 1H), 4.29-4.11 (m, 1H), 3.96-3.64 (m, 2H), 2.76 (d, *J* = 6.0 Hz, 2H), 2.39 (t, *J* = 7.2 Hz, 2H), 2.23-2.09 (m, 2H), 2.09-2.00 (m, 2H), 1.99 (s, 3H), 1.89-1.77 (m, 2H), 1.54-1.37 (m, 18H), 1.18 (dt, *J* = 13.5, 7.5 Hz, 1H), 0.98-0.81 (m, 6H). ESI-MS (m/z): calcd for C_30_H_50_N_4_O_10_ [M+Na]^+^: 649.35; found: 649.58.

**Synthesis of compound CyNA_P_N_3_IEPD-tBu.** Compound **CyNA_P_N_3_IEPD-tBu** was synthesized by similar procedures as **CyNA_P_N_3_IEFD-tBu**, using **CyNA_P_N_3_** (39 mg, 0.043 mmol), **IEPD** (27 mg, 0.043 mmol), O-Benzotriazole-N,N,N',N'-tetramethyl-uronium-hexaf (18 mg, 0.047 mmol) and N,N-Diisopropylethylamine (15 µL, 0.086 mmol), was purified using HPLC to obtain a purple solid (41 mg, 63% yield). ^1^H NMR (500 MHz, Methanol-*d*_4_) δ 8.75 (d, *J* = 15.4 Hz, 1H), 7.77 (s, 1H), 7.72 (t, *J* = 8.1 Hz, 2H), 7.60-7.53 (m, 4H), 7.18 (s, 1H), 6.92 (d, *J* = 15.5 Hz, 1H), 4.72 (t, *J* = 5.5 Hz, 2H), 4.66 (dd, *J* = 9.6, 4.7 Hz, 1H), 4.61 (t, *J* = 5.7 Hz, 1H), 4.58-4.55 (m, 1H), 4.46 (dd, *J* = 8.3, 3.9 Hz, 1H), 4.33 (dd, *J* = 8.4, 5.3 Hz, 1H), 4.20 (t, *J* = 4.7 Hz, 2H), 4.00 (t, *J* = 5.0 Hz, 2H), 3.90 (s, 2H), 3.85-3.81 (m, 2H), 3.59-3.56 (m, 2H), 3.51-3.48 (m, 4H), 3.39 (t, *J* = 5.7 Hz, 2H), 3.23-3.21 (m, 1H), 3.15 (dd, *J* = 13.9, 4.8 Hz, 1H), 2.93 (dd, *J* = 14.0, 9.0 Hz, 1H), 2.82-2.78 (m, 4H), 2.76-2.71 (m, 4H), 2.38 (q, *J* = 6.4 Hz, 4H), 2.06 (q, *J* = 7.6 Hz, 2H), 1.99 (s, 6H), 1.96-1.94 (m, 2H), 1.86 (s, 6H), 1.44-1.42 (m, 18H), 1.17 (q, *J* = 7.3 Hz, 2H), 0.91-0.89 (m, 6H). ESI-MS (m/z): calcd for C_69_H_95_N_12_O_14_S_2_^+^ [M]^+^: 1379.65; found: 1379.85.

**Synthesis of compound CyNA_P_N_3_IEPD.** Compound **CyNA_P_N_3_IEPD** was synthesized by similar procedures as **CyNA_P_N_3_IEFD**, using **CyNA_P_N_3_IEPD-tBu** (45 mg, 0.03 mmol) and 10 mL dicyclohexylcarbodiimide / trifluoroacetic acid (4:1, v/v), was purified using HPLC to obtain a purple solid (23 mg, 55% yield). ^1^H NMR (500 MHz, Methanol-*d*_4_) δ 8.76 (d, *J* = 15.4 Hz, 1H), 7.77 (s, 1H), 7.72 (t, *J* = 6.8 Hz, 2H), 7.60-7.53 (m, 4H), 7.18 (s, 1H), 6.93 (d, *J* = 15.5 Hz, 1H), 4.72 (t, *J* = 5.0 Hz, 2H), 4.67 (dd, *J* = 8.5, 5.5 Hz, 1H), 4.57-4.52 (m, 2H), 4.31 (dd, *J* = 8.4, 5.3 Hz, 1H), 4.20 (d, *J* = 7.9 Hz, 1H), 4.01 (t, *J* = 5.0 Hz, 2H), 3.89 (s, 2H), 3.86 (t, *J* = 6.3 Hz, 1H), 3.79 (q, *J* = 5.4, 4.4 Hz, 1H), 3.58-3.55 (m, 2H), 3.51-3.47 (m, 4H), 3.40 (t, *J* = 6.0 Hz, 2H), 3.18 (dd, *J* = 13.6, 4.5 Hz, 2H), 2.94 (dd, *J* = 14.0, 9.7 Hz, 1H), 2.79 (t, *J* = 6.8 Hz, 4H), 2.76 (d, *J* = 6.3 Hz, 1H), 2.73 (t, *J* = 5.8 Hz, 2H), 2.44 (t, *J* = 7.1 Hz, 2H), 2.23-2.14 (m, 2H), 2.07-2.02 (m, 1H), 1.98 (s, 3H), 1.94 (p, *J* = 6.0 Hz, 4H), 1.86 (s, 6H), 1.81-1.77 (m, 1H), 1.50 (ddd, *J* = 11.3, 7.3, 3.3 Hz, 1H), 1.19-1.14 (m, 1H), 0.90-0.86 (m, 6H). HRMS (m/z): calcd for C_61_H_79_N_12_O_14_S_2_^+^ [M]^+^: 1267.5275; found: 1267.5276.

**Synthesis of probe BGR_H_.** Probe **BGR_H_** was synthesized by similar procedures as **BGR_M_**, using **CyNA_P_N_3_IEPD** (20 mg, 0.014 mmol), **Propynyl-HPβCD** (34 mg, 0.021 mmol), CuSO_4_**·**5H_2_O (1 mg, 0.004 mmol) and sodium ascorbate (1 mg, 0.004 mmol), was purified by dialysis and lyophilized to give a purple powder (33 mg, 69% yield). ^1^H NMR (500 MHz, Methanol-*d*_4_) δ 8.75 (d, *J* = 15.1 Hz, 1H), 8.09 (s, 1H), 7.78-7.70 (m, 3H), 7.61-7.52 (m, 4H), 7.18 (s, 1H), 6.94 (d, *J* = 15.6 Hz, 1H), 5.14-5.10 (m, 8H), 4.65-4.56 (m, 12H), 3.83-3.74 (m, 86H), 3.16 (t, *J* = 1.6 Hz, 2H), 2.79 (t, *J* = 6.0 Hz, 2H), 2.72 (t, *J* = 6.5 Hz, 2H), 2.35-2.23 (m, 4H), 2.01-1.98 (m, 6H), 1.86 (s, 6H), 1.73-1.69 (m, 2H), 1.60 (t, *J* = 7.1 Hz, 4H), 1.49-1.43 (m, 1H), 1.15-1.13 (m, 30H), 0.90-0.88 (m, 8H). MALDI-TOF MS: found: 2550-2900.

**2. Supplementary figures and Tables**

**Figure S1 The synthetic route of BGR_M_.**

**Figure S2 The synthetic route of BGR_H_.**


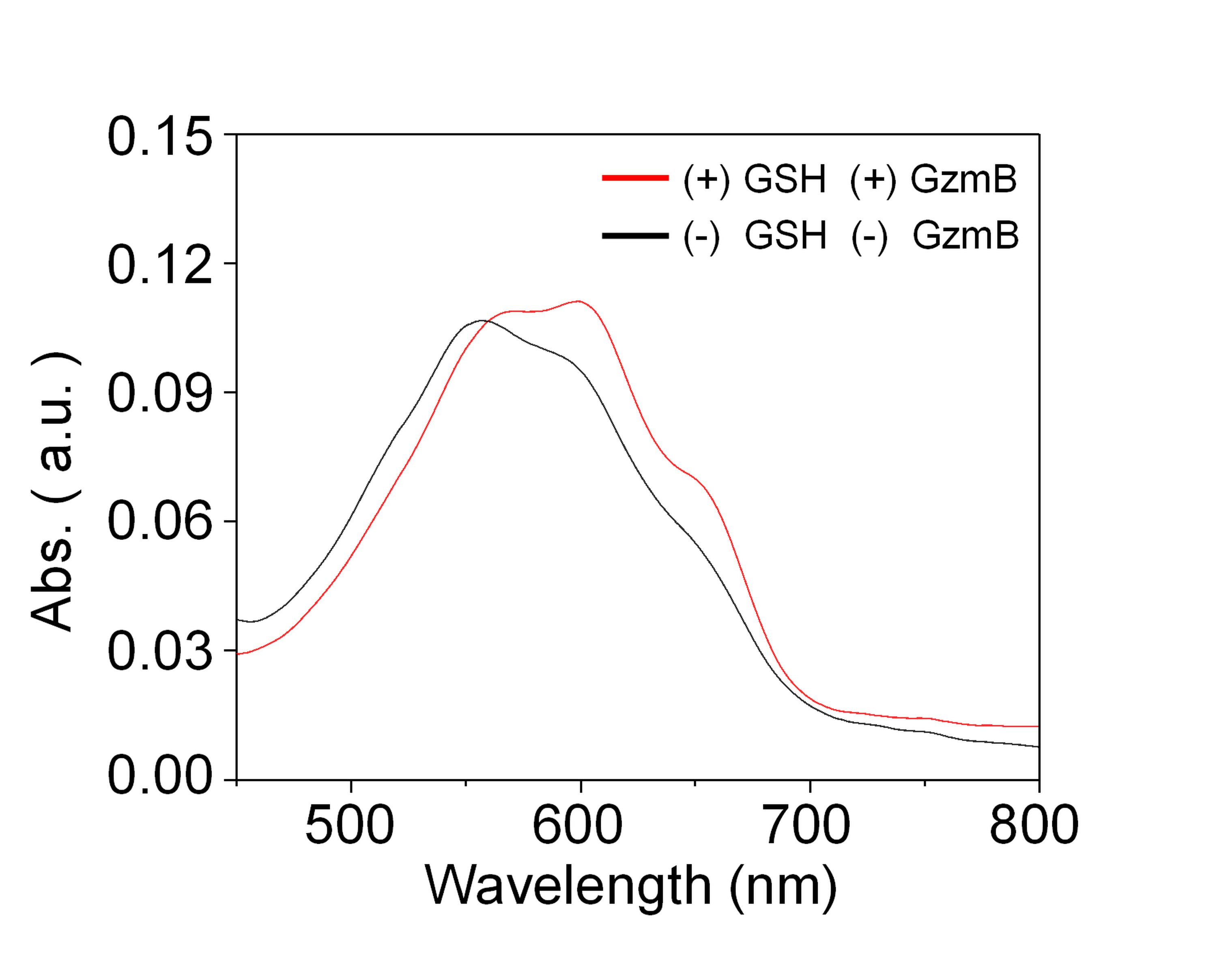


**Figure S3 Absorption spectra for BGR_M_**. Absorption spectra of BGR_M_ in the absence and presence of mouse GzmB and GSH at 37 °C in PBS buffer (pH 7.4). The experiments were repeated independently three times with similar results.


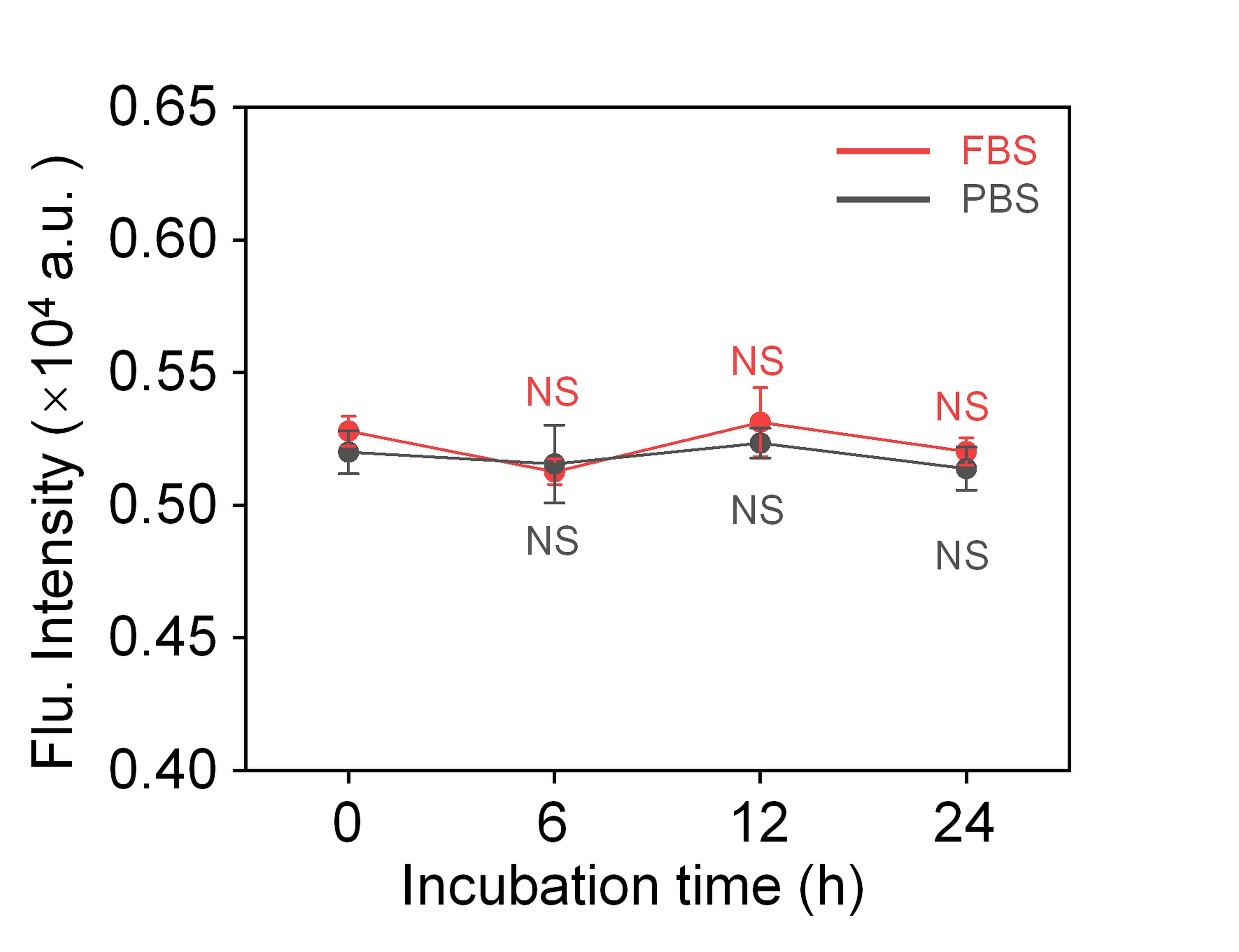


**Figure S4 Stability of BGR_M_.** Fluorescence intensity of mouse BGR_M_ was measured after incubation in PBS and FBS for 0, 6, 12, and 24 h.


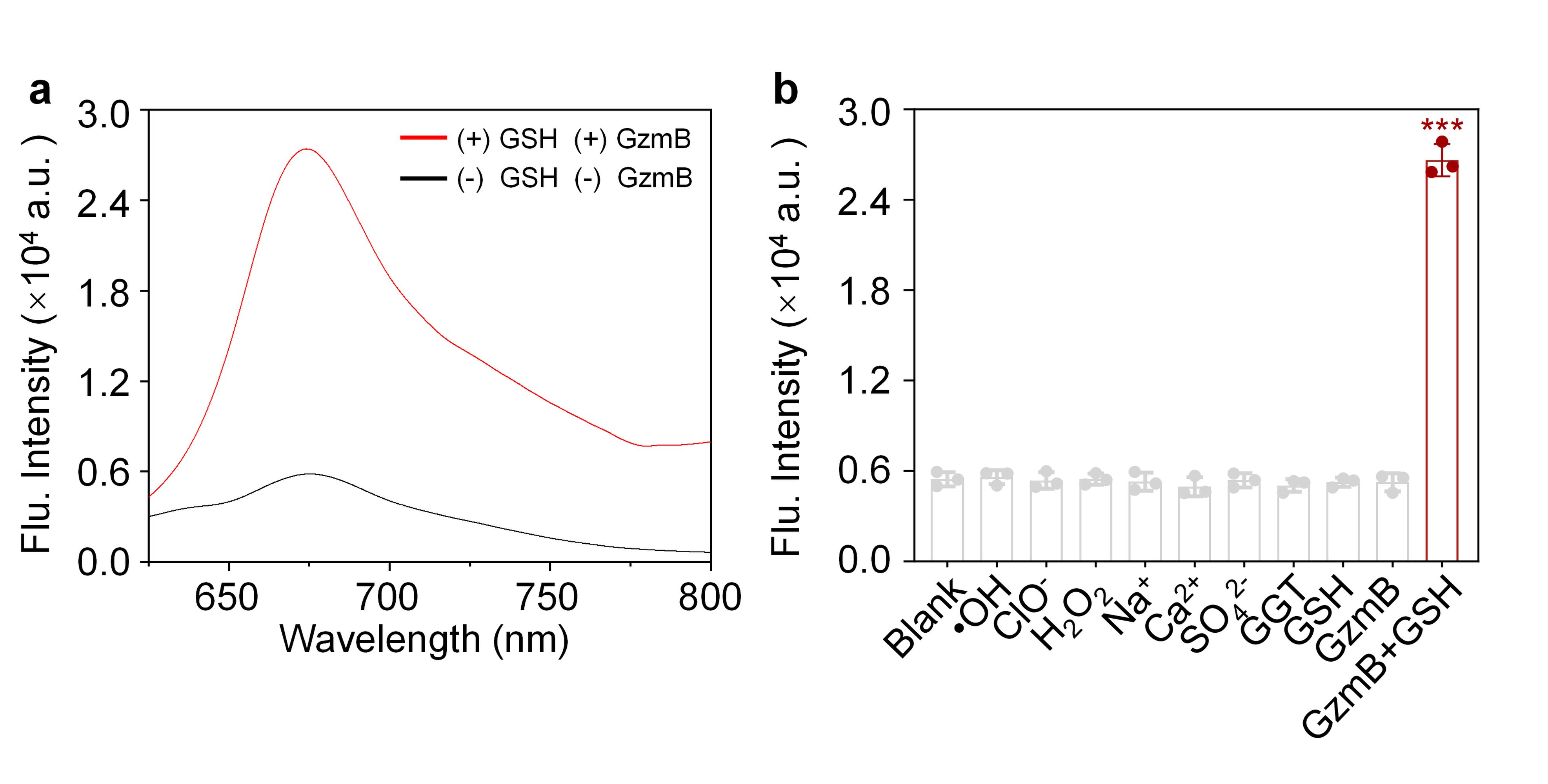


**Figure S5** (a) Fluorescence spectrum of BGR_H_ in the absence and presence of human GzmB and GSH at 37 °C in PBS buffer (pH 7.4). Fluorescence excitation at 580 nm. (b) Fluorescence intensity of **BGR_H_** (20 µM) after incubation with the indicated enzymes, metal ions (60 µM), ROS (60 µM) in PBS (10 mM, pH 7.4) at 37 °C. OH·, hydroxyl radical; ClO^-^, sodium hypochlorite; H_2_O_2_, hydrogen peroxide; Na^+^, sodium chloride; Ca^2+^, calcium chloride; SO_4_^2-^, sodium sulfate; GGT, gamma-glutamyl transferase; GSH, glutathione; GzmB, granzyme B (n = 3, mean ± s.d.). Two-tailed Student’s t test; GSH/GzmB treated group versus blank group, ****P* < 0.001.


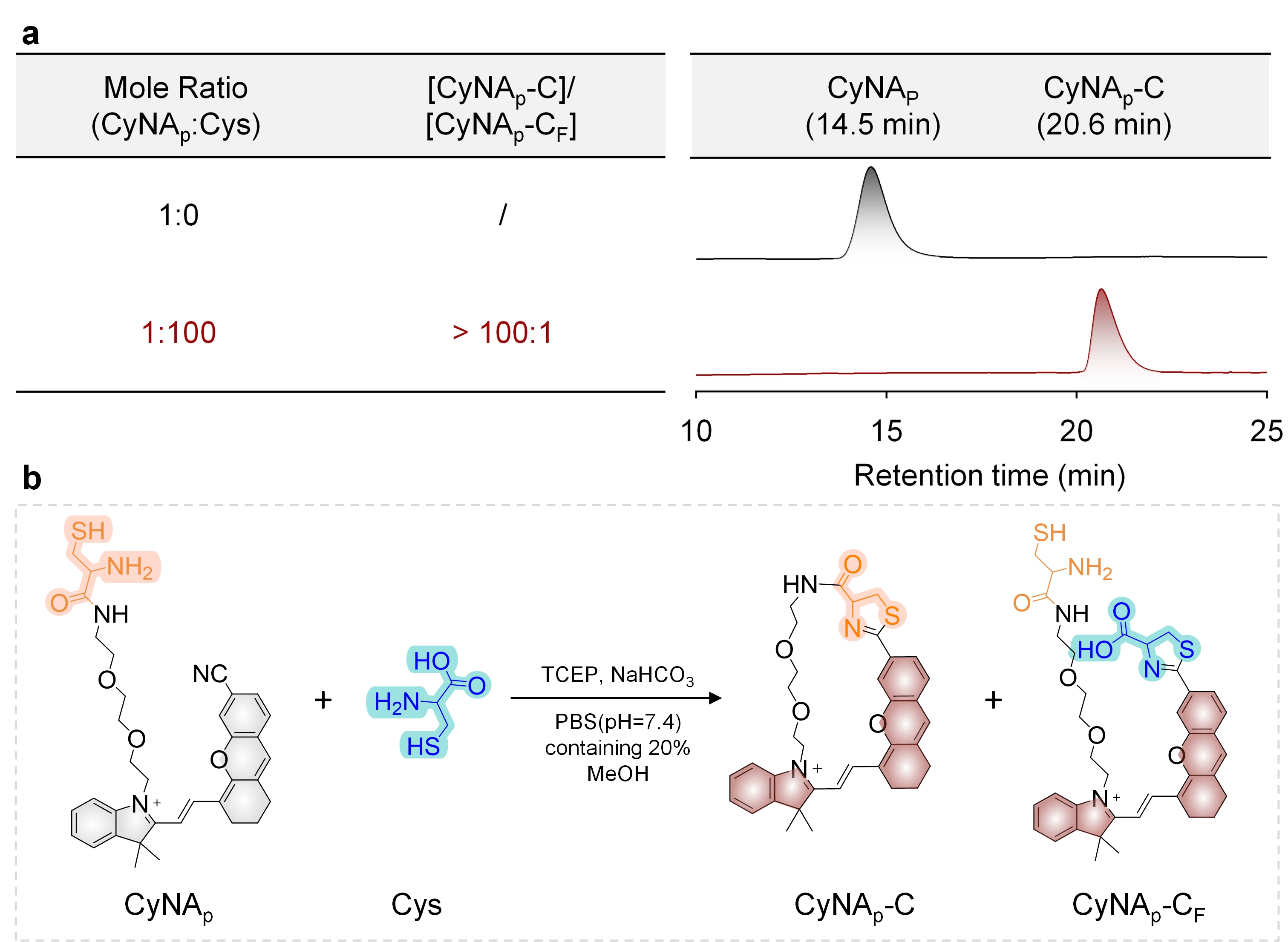


**Figure S6** (a) A competitive HPLC assay of CyNA_P_ incubated with free cysteine at 1:100 molar ratio. (b) Chemical reactions for intramolecular and intermolecular condensation.


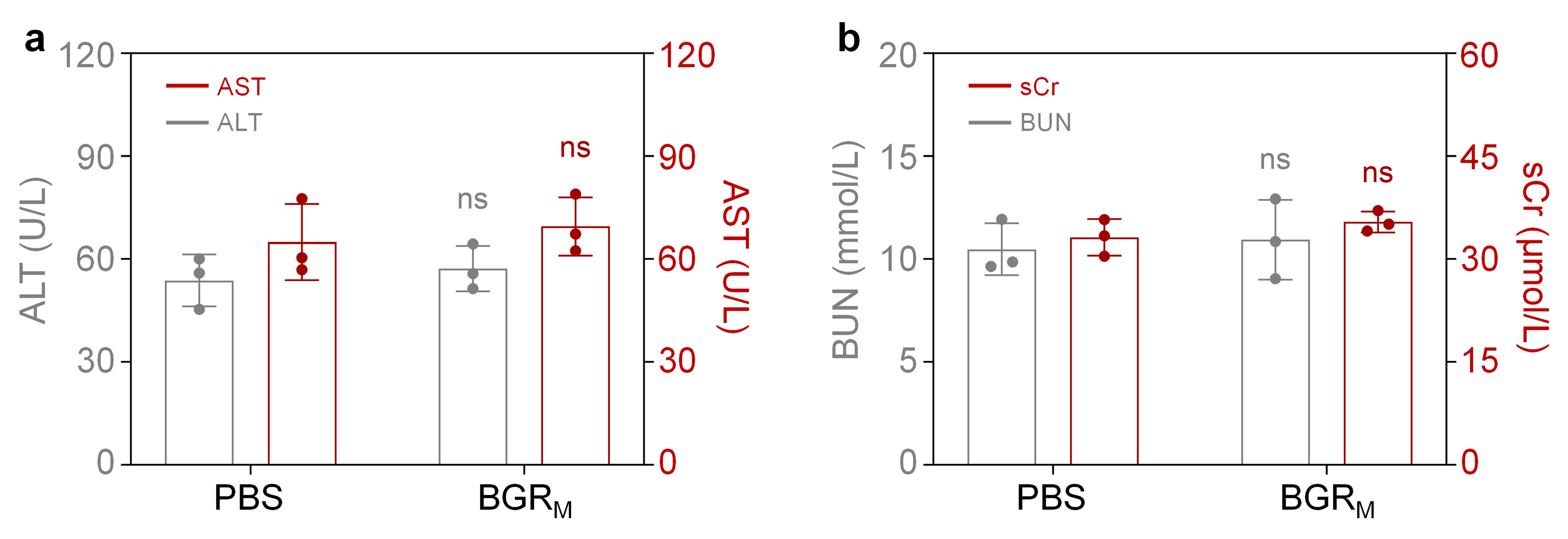


**Figure S7** Measurements of levels of ALT/AST (a) and BUN/sCr (b) from mice after 24 h injection of PBS or BGR_M_ (n=3, mean ± s.d.). Two-tailed Student’s *t*-test; PBS group versus BGR_M_-treated group. ns: no statistically significant differences.


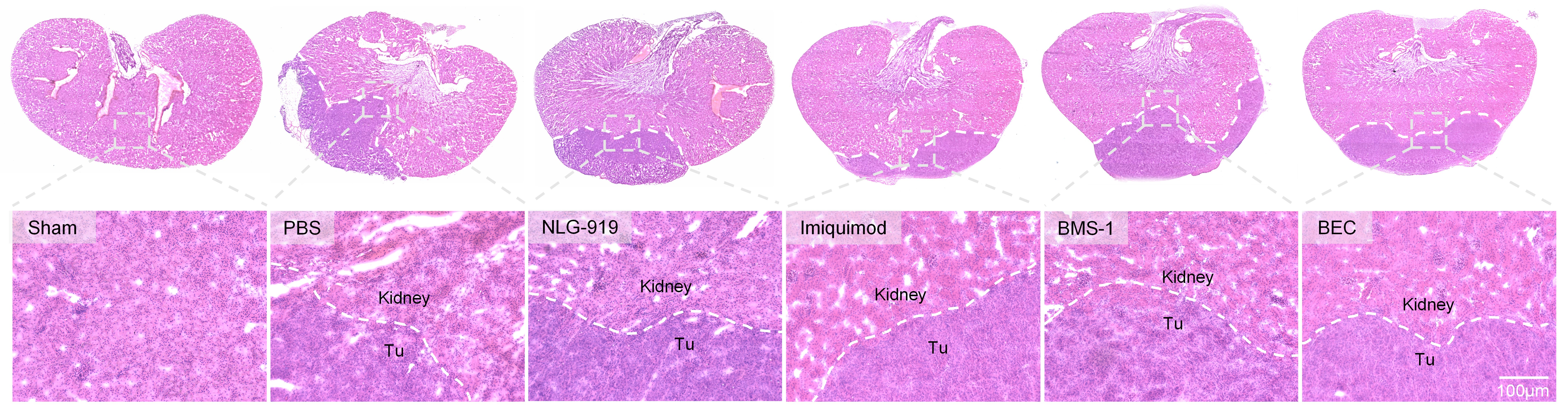


**Figure S8** Representative photomicrographs of H&E staining of kidney sections from mice in different groups.


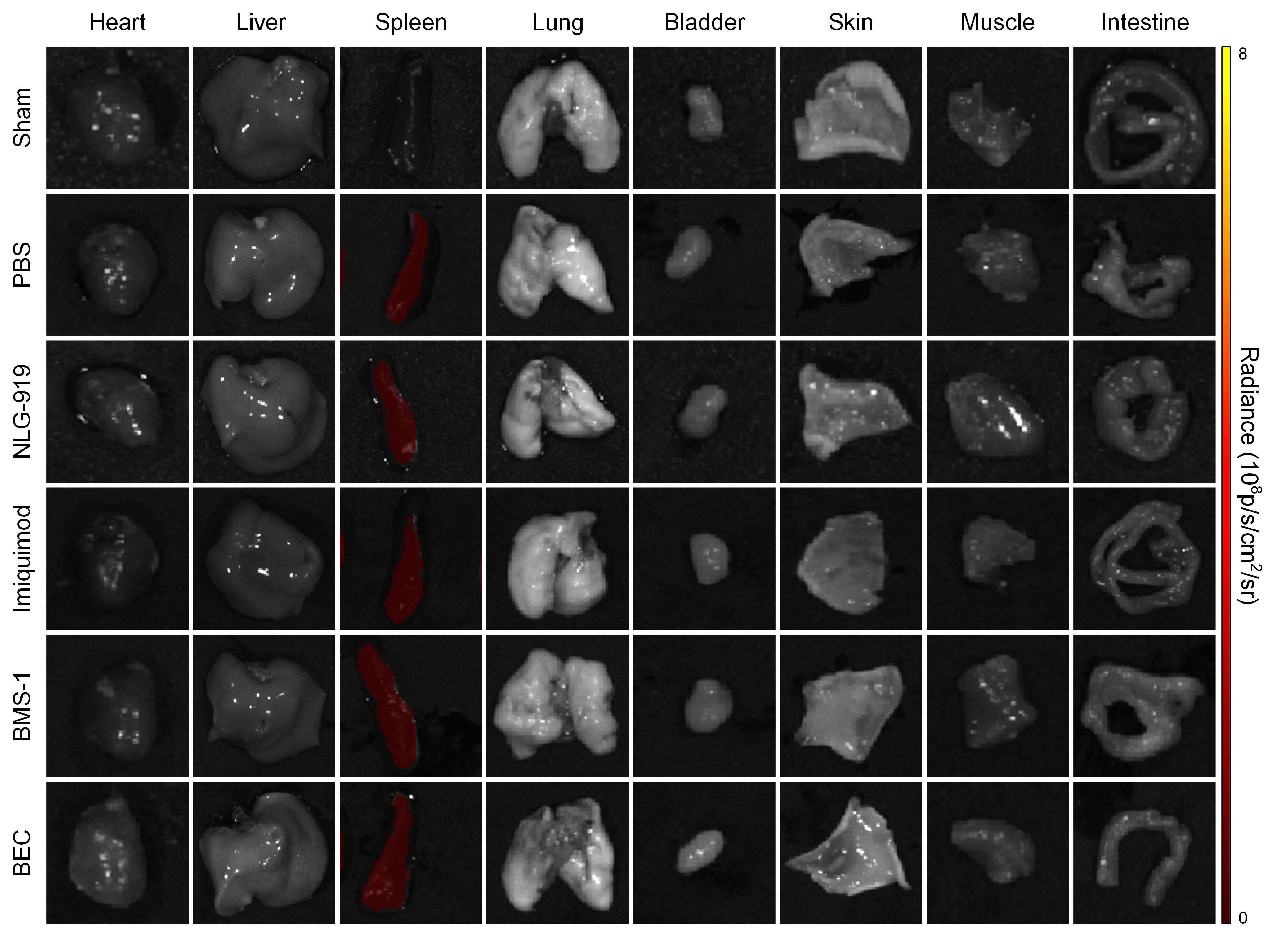


**Figure S9 *Ex vivo* NIRF imaging of resected organs from different group mice.** *Ex vivo* NIRF images of major organs including heart, liver, lung, spleen, bladder, skin, muscle, intestine at 1 h post-injection timepoint of BGR_M_.


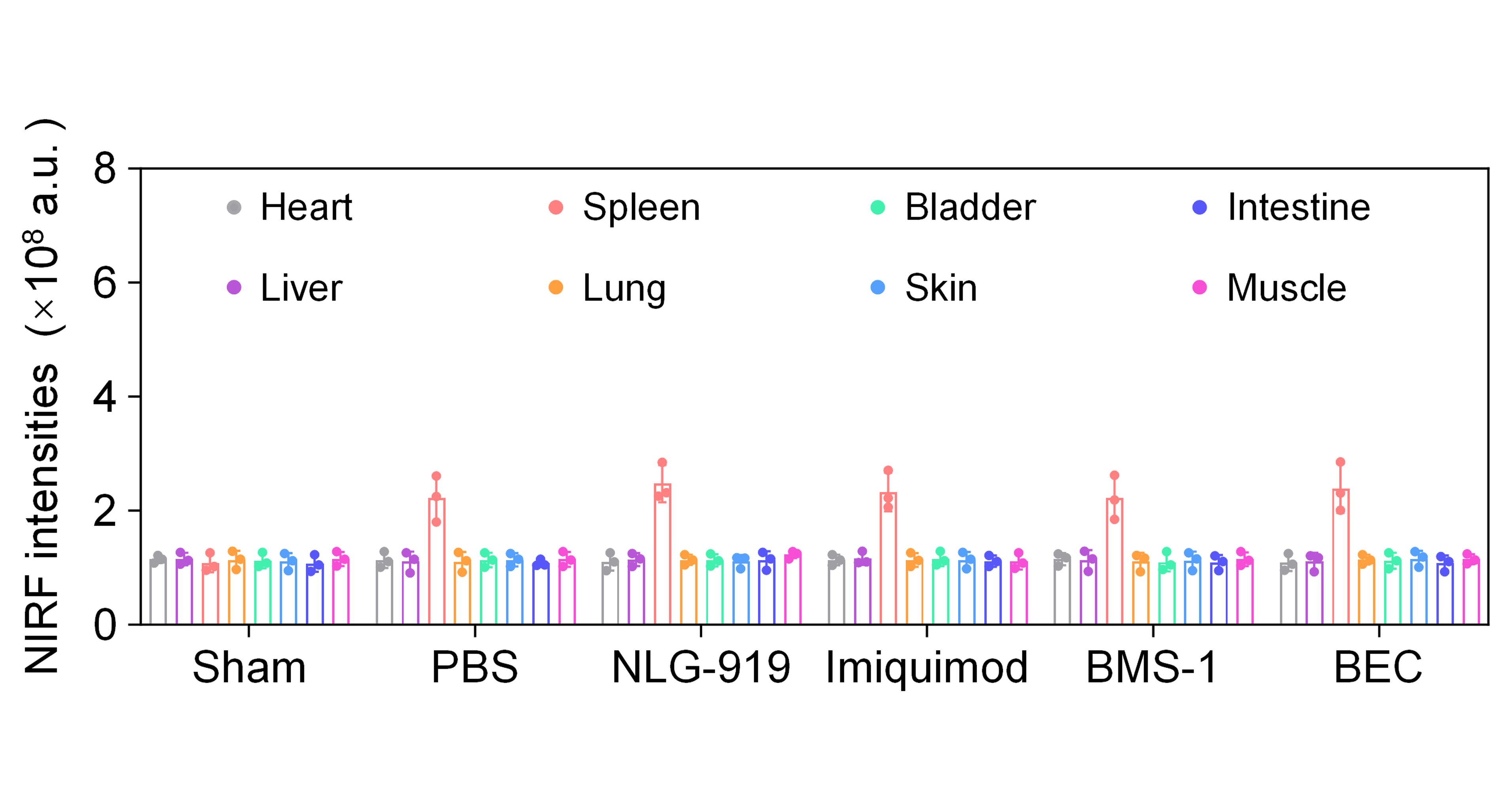


**Figure S10 *E*x vivo NIRF quantification of major organs from different groups mice at t = 1 h postinjection of BGR_M_.** The NIRF images acquired at 675 nm upon excitation at 580 nm with the IVIS spectrum imaging system. Data are the mean ± SD. n = 3 independent mice.


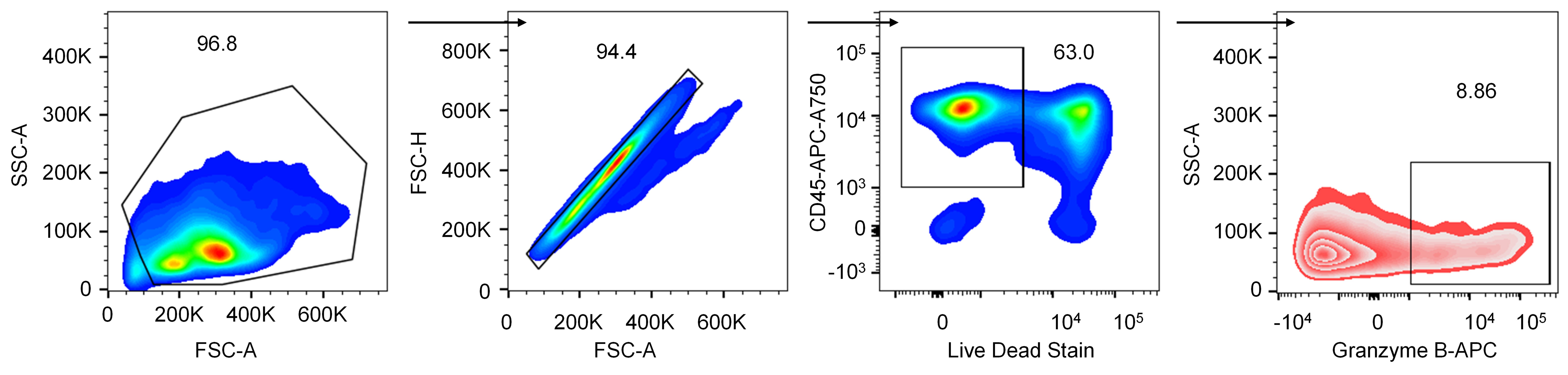


**Figure S11** Gating strategy for flow cytometry analysis of GzmB^+^ leukocytes.


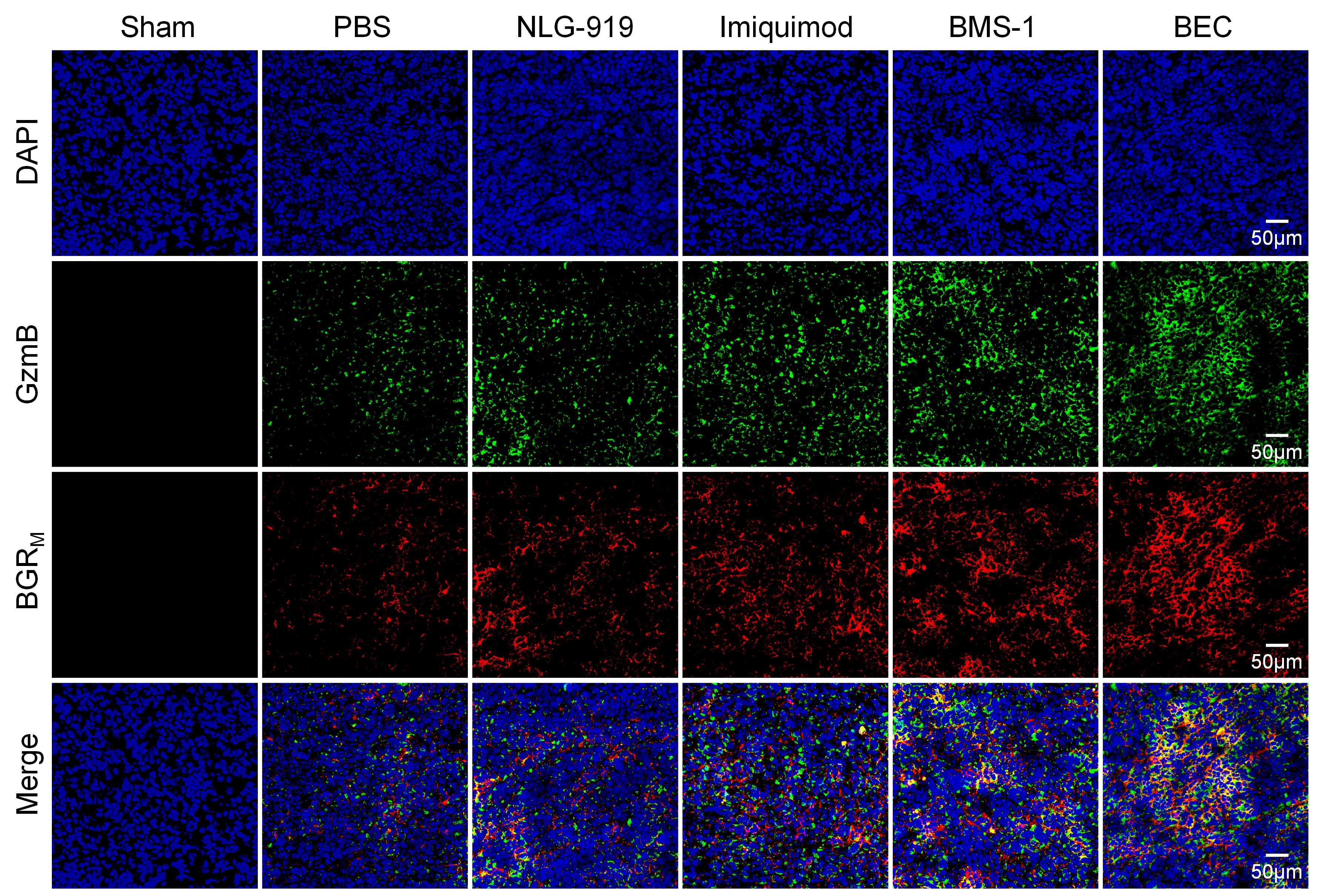


**Figure S12** Representative immunofluorescence staining images of tumor-bearing kidneys of immunotherapeutics-treated mice. Scale bar = 100 μm.


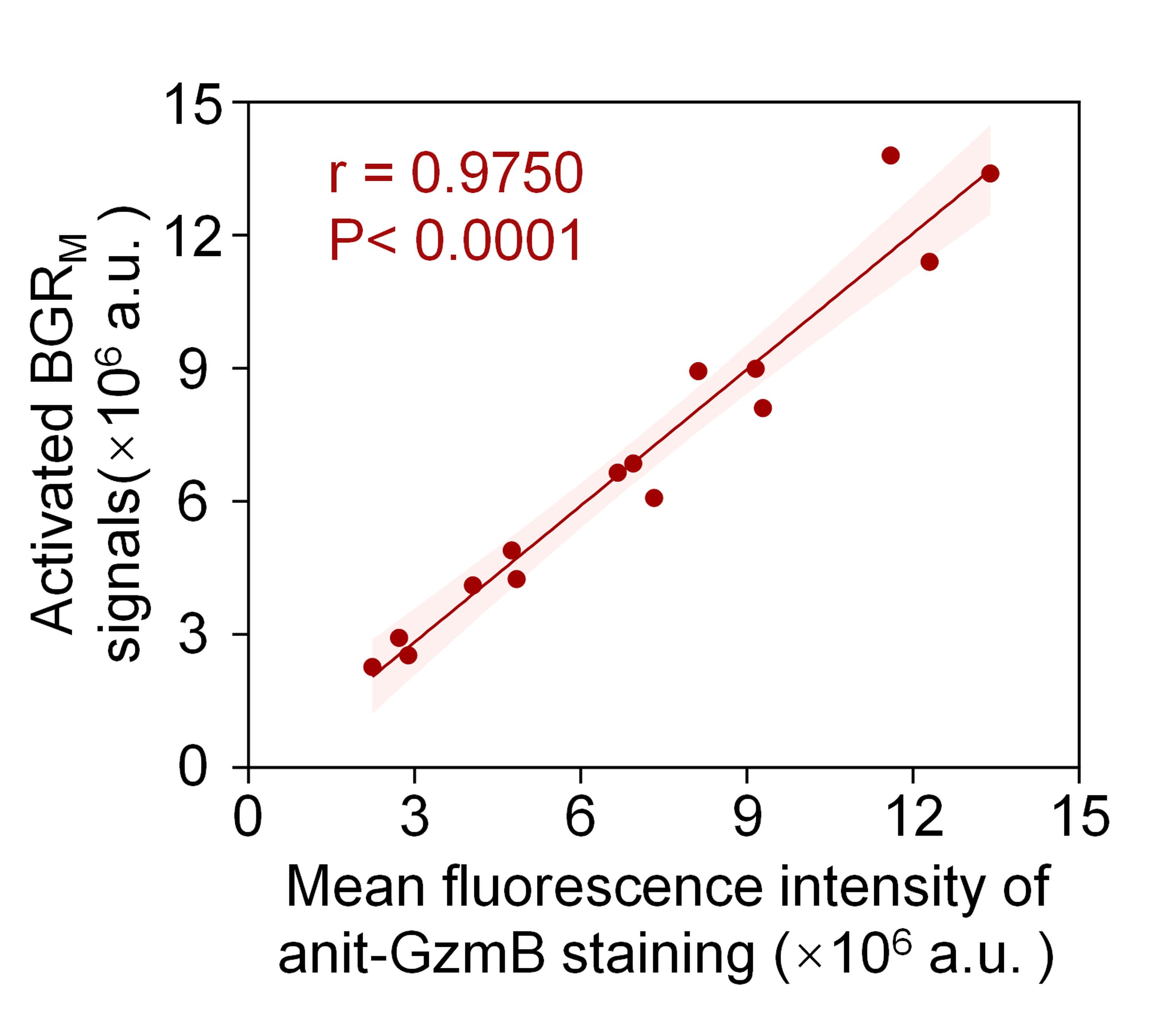


**Figure S13** Correlation between mean fluorescence intensity of anit-GzmB staining and activated BGR_M_ signals in tumor-bearing kidneys via a simple linear regression model. The 95% confidence intervals were obtained by two-tailed Student’s t-test analysis.


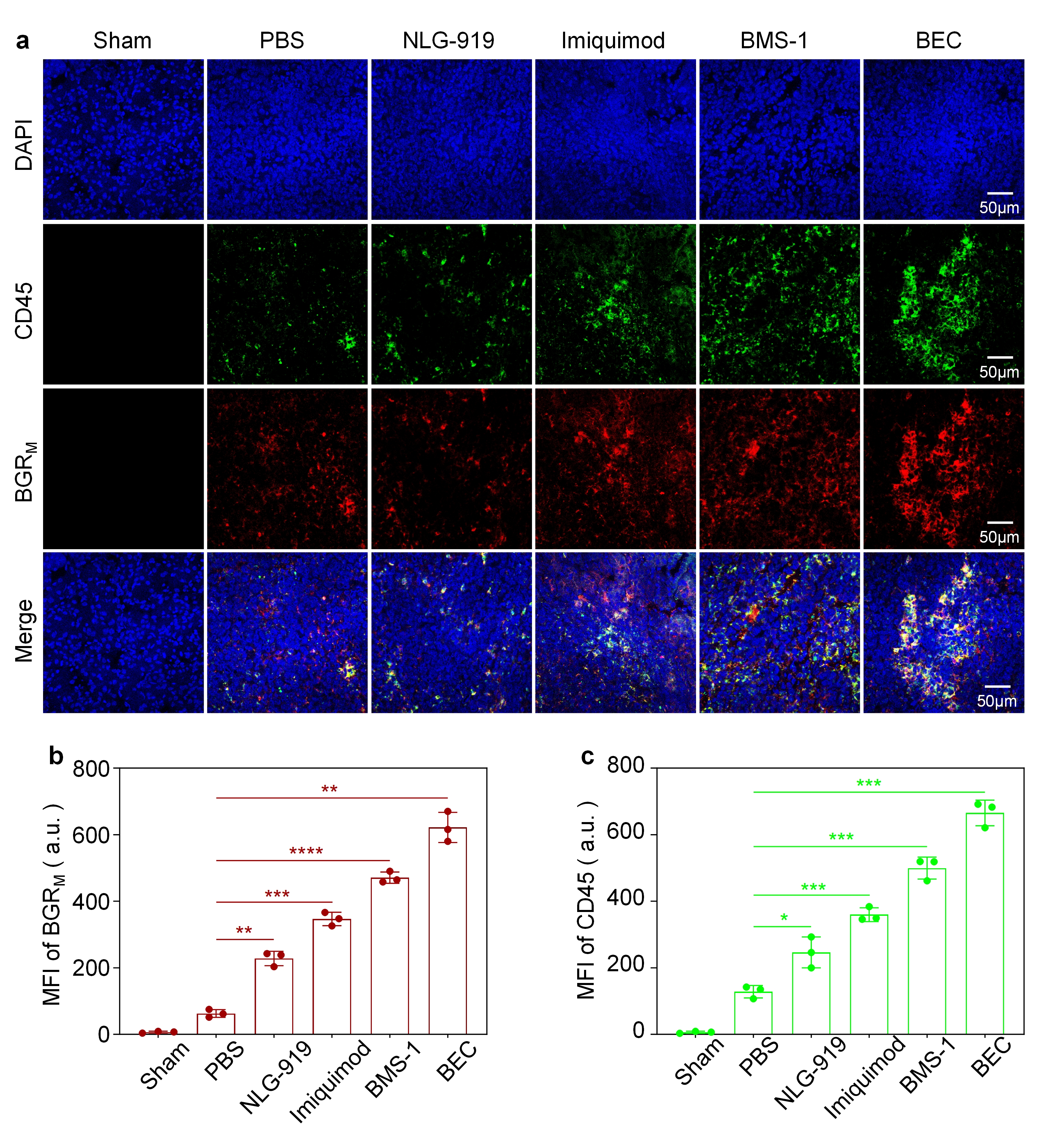


**Figure S14** (a) Representative confocal fluorescence images of regional tumor slices from immunotherapeutics-treated mice. The blue signal is from 4′,6-diamidino-2-phenylindole (DAPI); the green signal is from CD45 antibody; the red signal is from activated BGRM. (b,-c) Mean fluorescence intensity (MFI) of tumor sections from mice treated with different immunotherapeutics (n=3, mean ± s.d.).


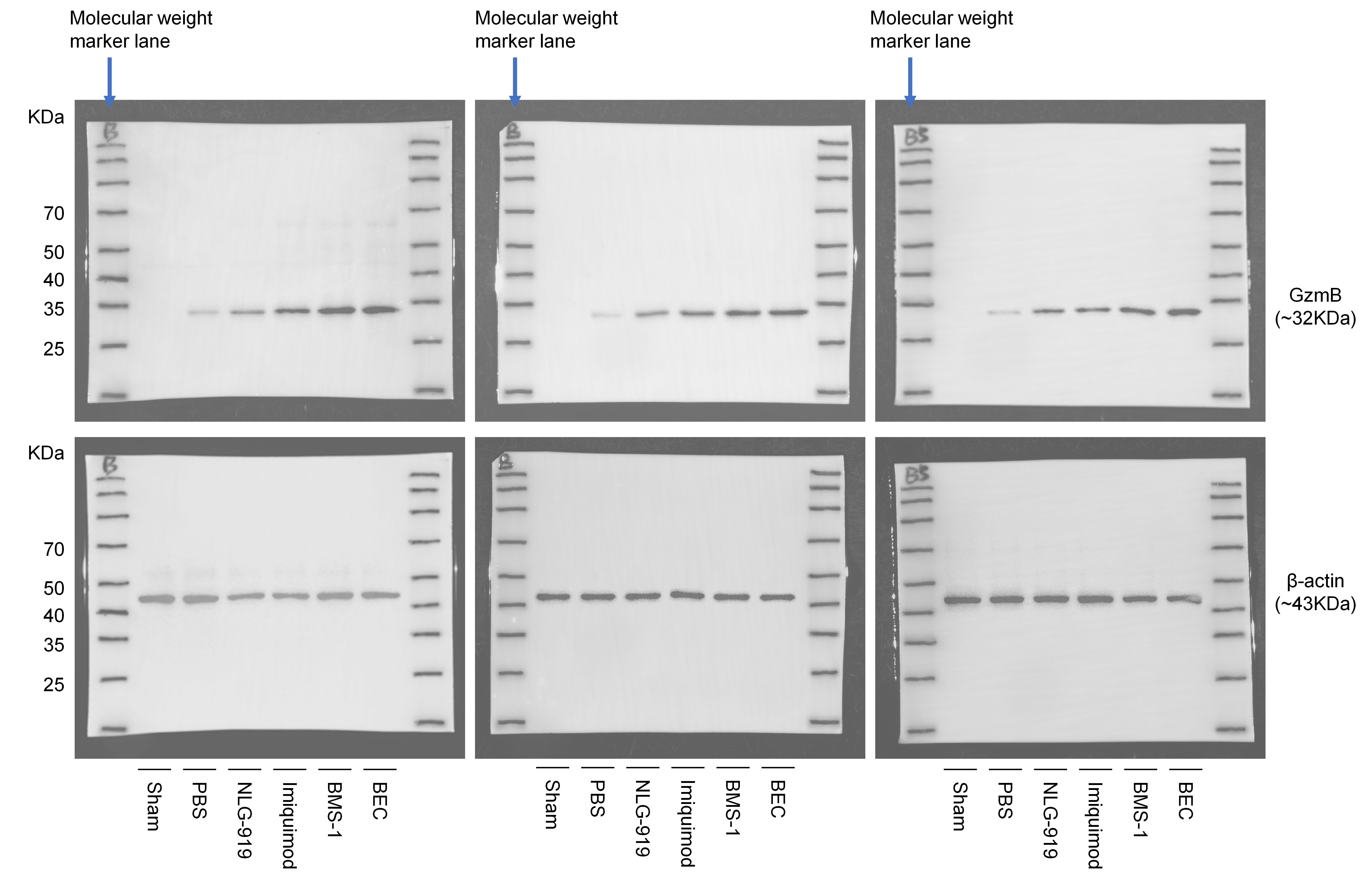


**Figure S15** Uncropped images of western blots showing in Figure 6b.


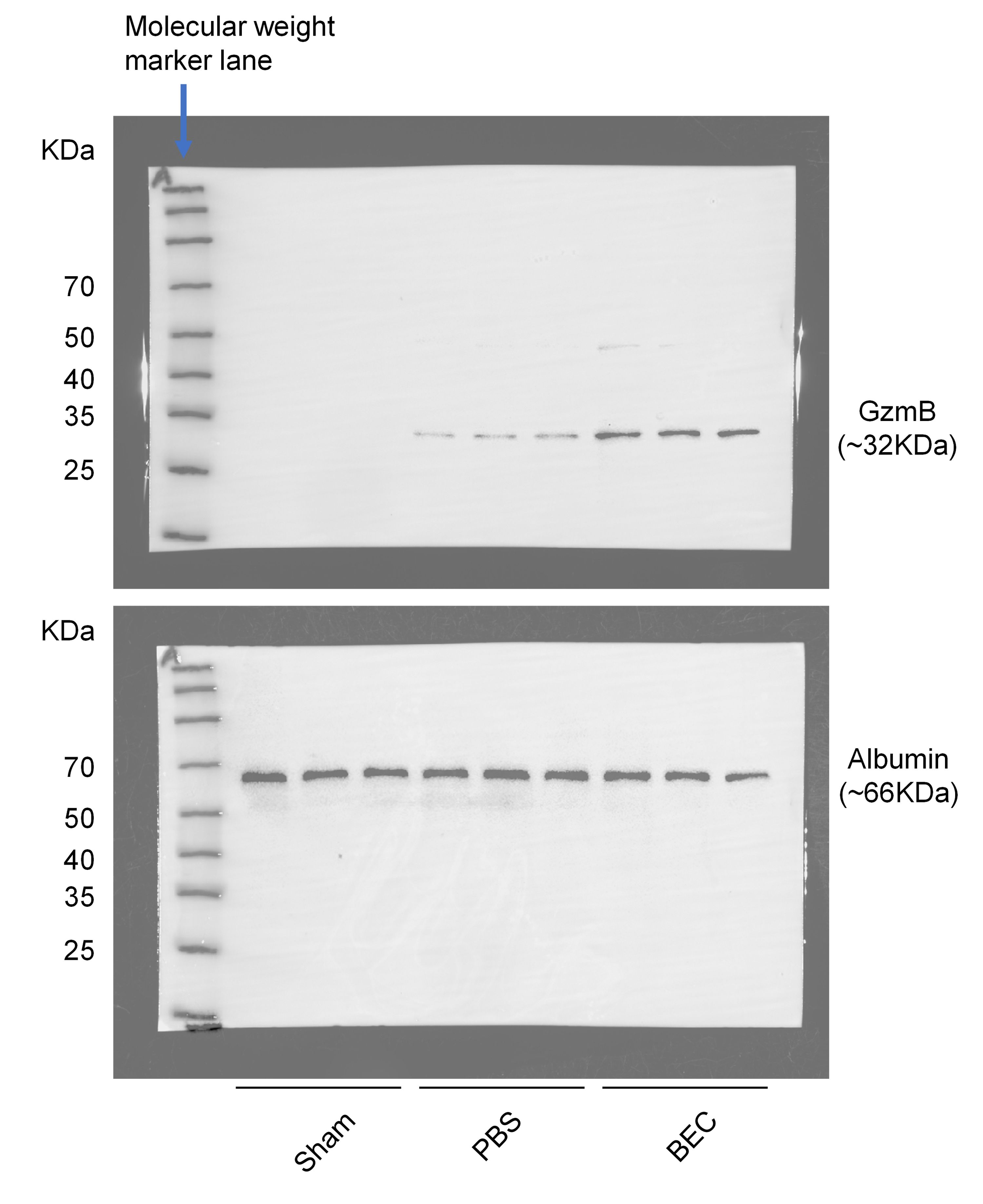


**Figure S16** Uncropped images of western blots showing in Figure 6c.


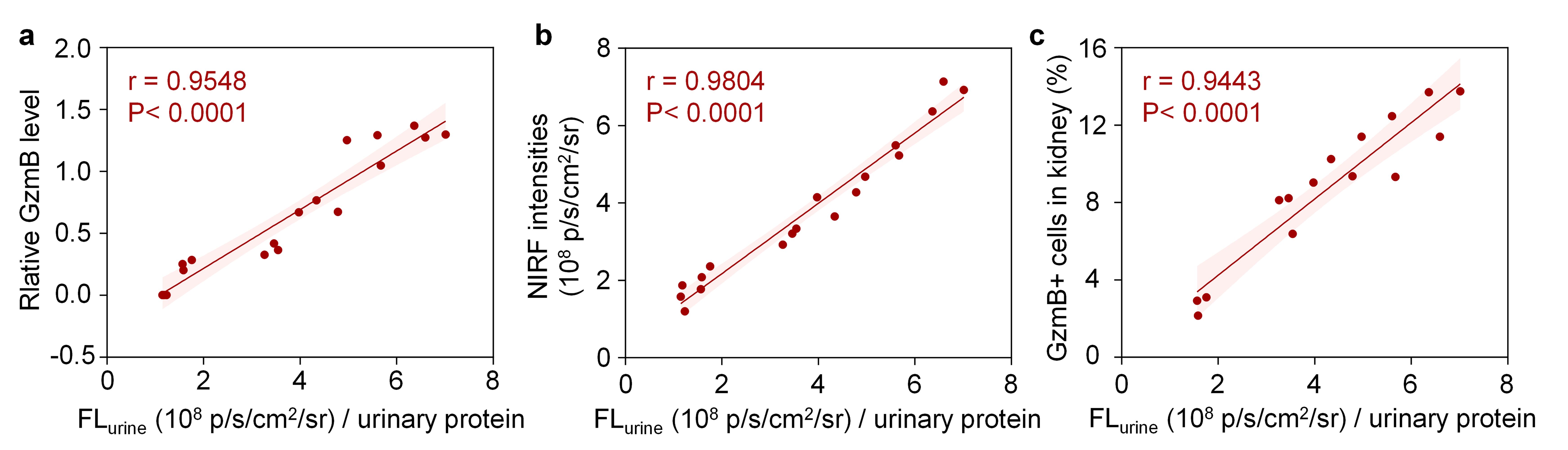


**Figure S17** Correlation between urinalysis and immunoblotting analyses (a), in vivo NlRF imaging (b), and ex vivo flow cytometry assay (c). The 95% confidence intervals were obtained by two-tailed Student’s t-test analysis.


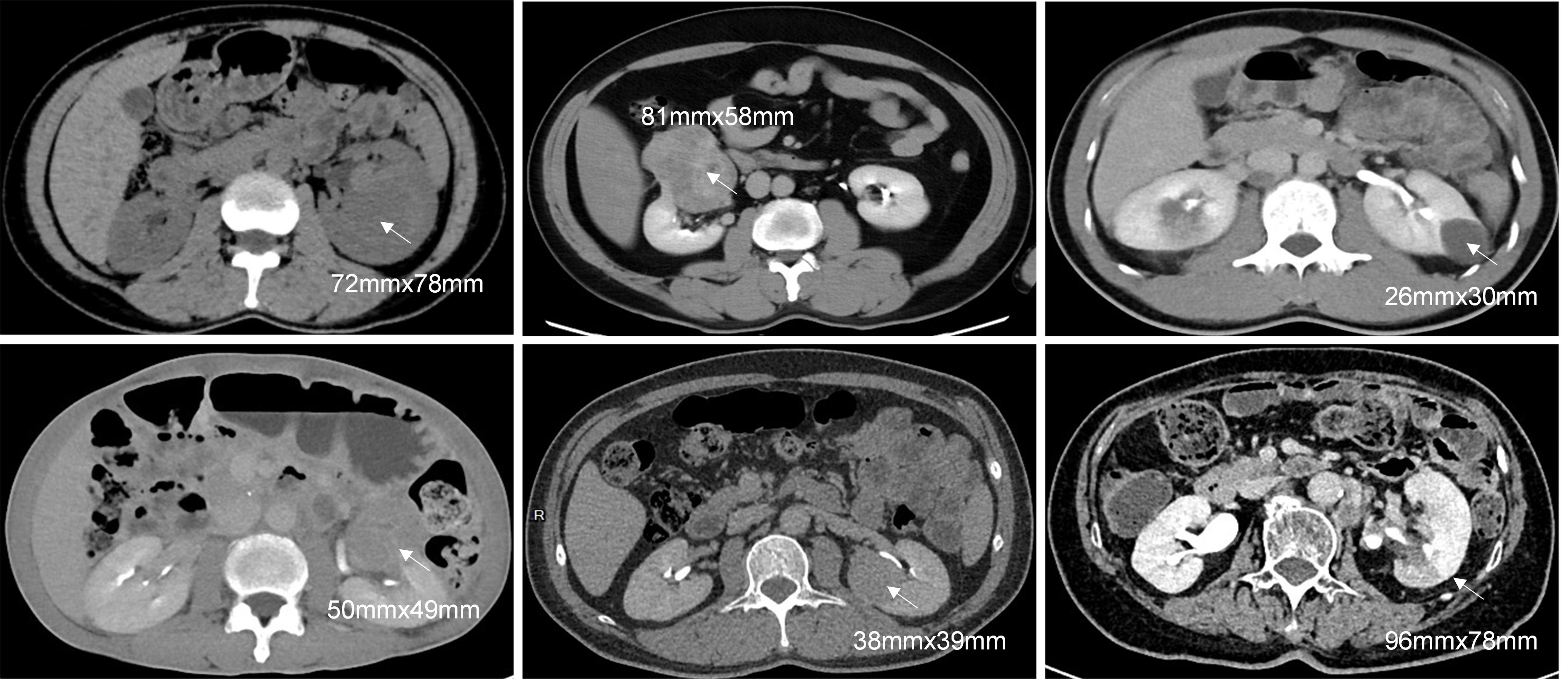


**Figure S18** Representative CT images of RCC patients. White arrows indicate the location of kidney tumor and their sizes.


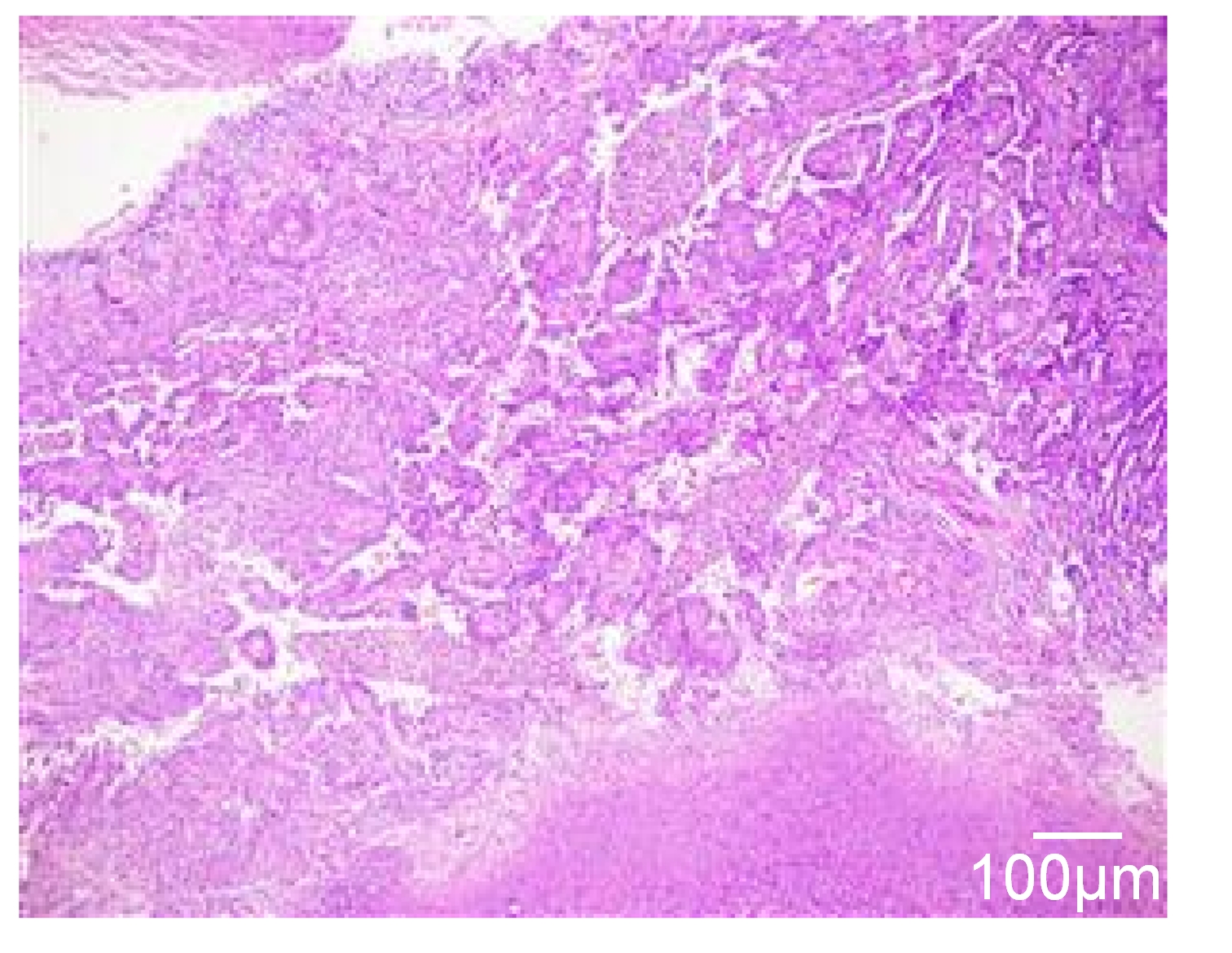


**Figure S19** Representative HE image of kidney tumor from RCC patients.


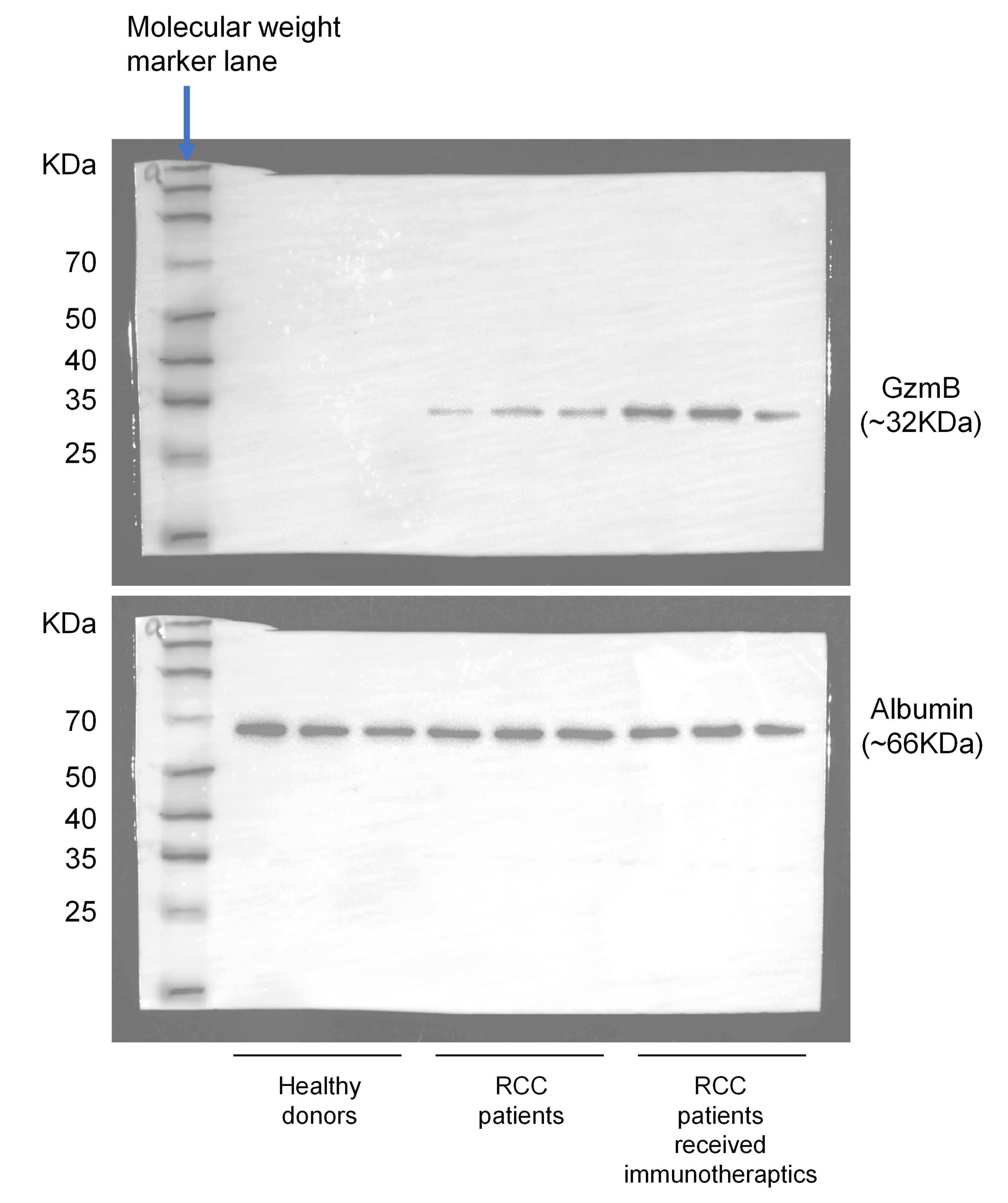


**Figure S20** Uncropped images of western blots showing in Figure 7b.


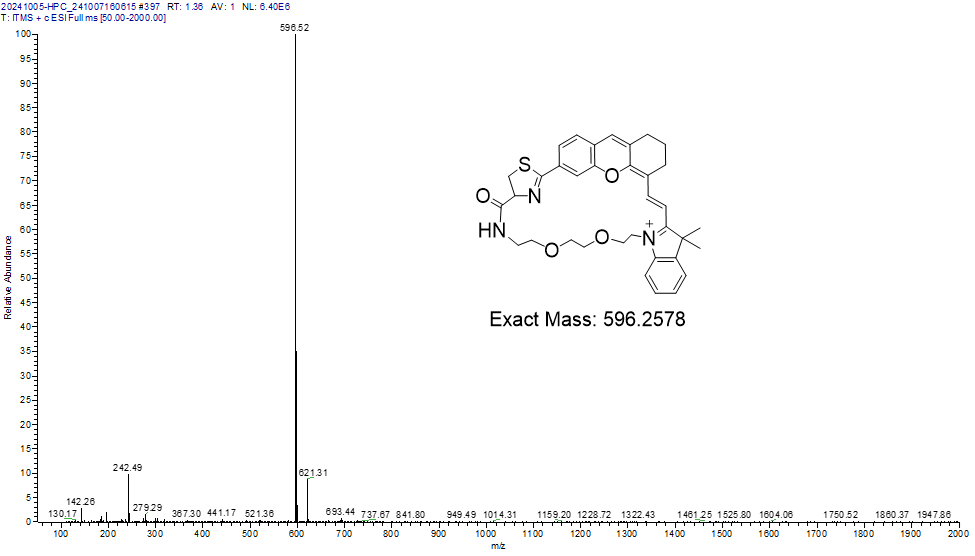


**Figure S21** The ESI-MS spectrum of urine sample incubated with BGR_H_. The mass spectra are identical to that of CyNA_p_-C, confirming the activation of BGR_H_ by urinary GzmB.


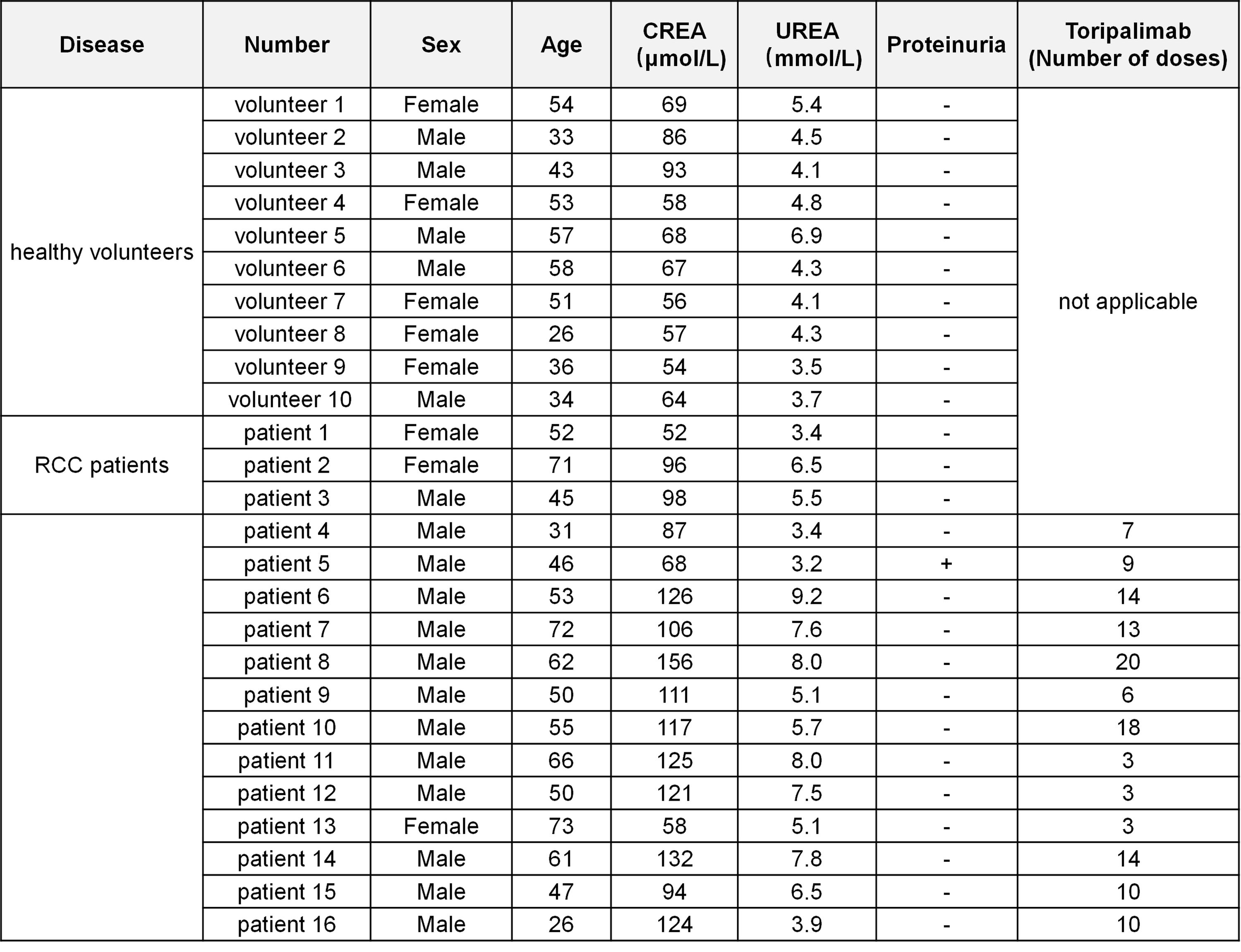


**Table S1** Information sheet for healthy volunteers, RCC patients and RCC patients with immunotherapy. Proteinuria (“-” means negative; “+” means positive). Toripalimab was administered as an intravenous injection once every 4 weeks, whereas axitinib was given orally twice daily on a schedule of three weeks on treatment followed by one week off. Treatment was discontinued immediately upon the development of resistance.


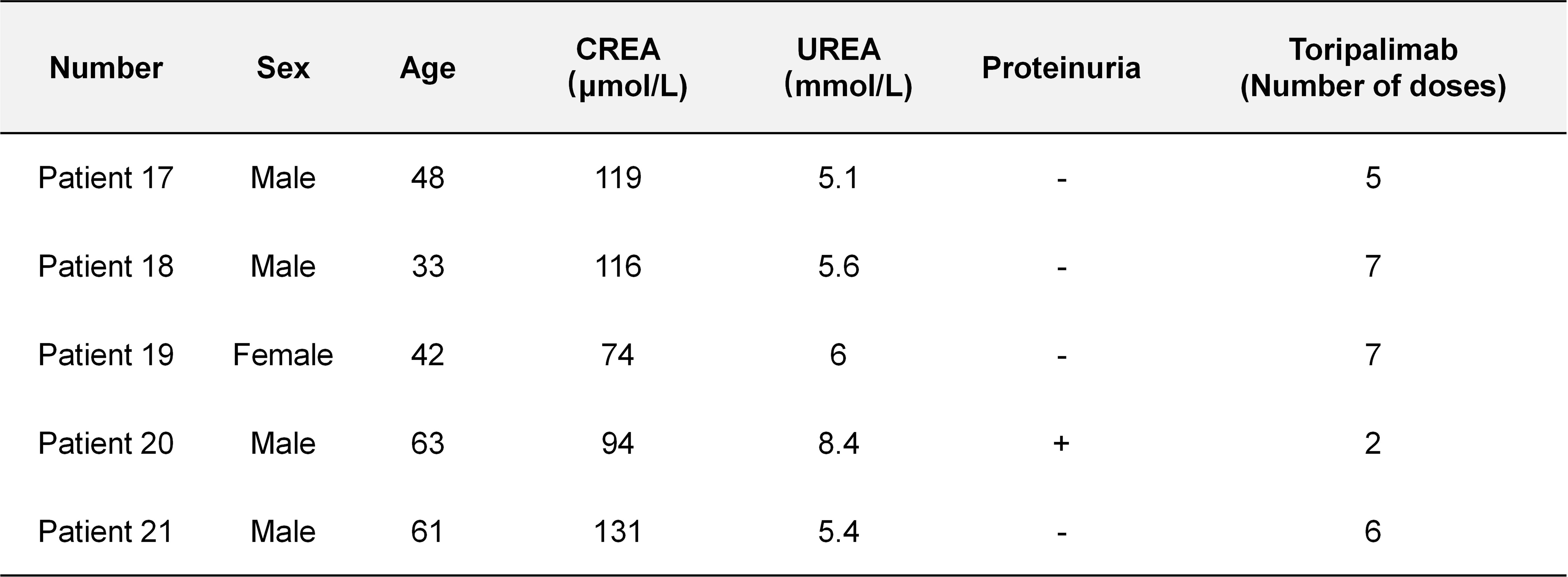


**Table S2** Information sheet for RCC patients before and after immunotherapy.

**3. NMR and MS spectroscopy**


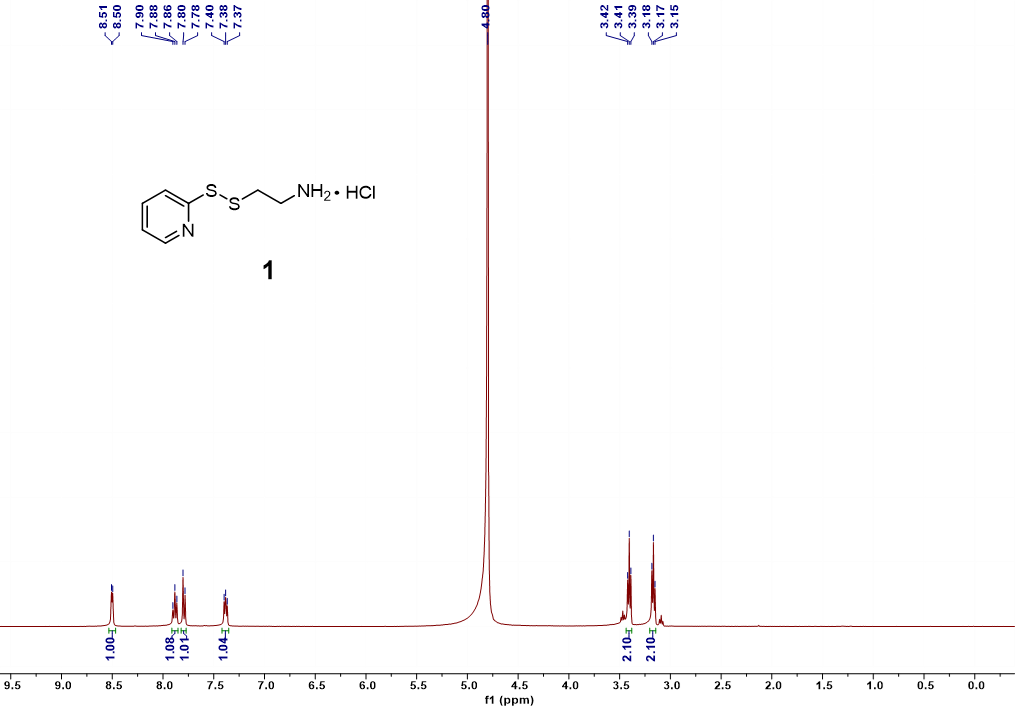


The ^1^H NMR spectrum (400 MHz) of compound **1** in D_2_O.


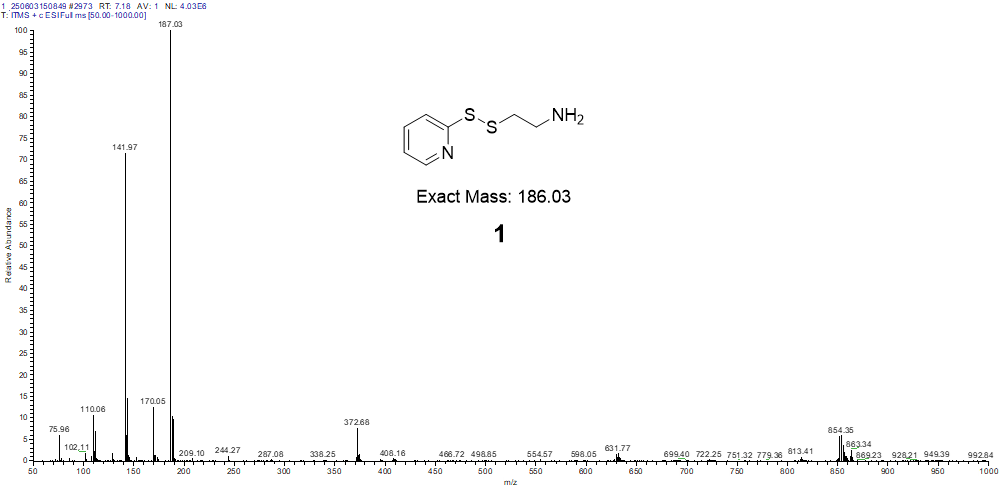


The ESI-MS spectrum of compound **1**.


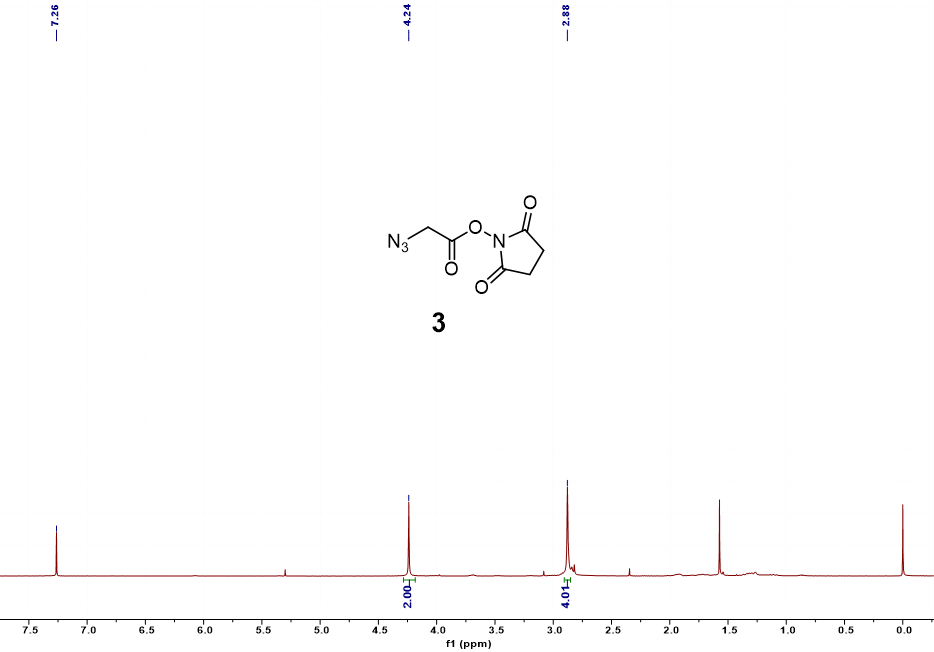


The ^1^H NMR spectrum (400 MHz) of compound **3** in CDCl_3_.


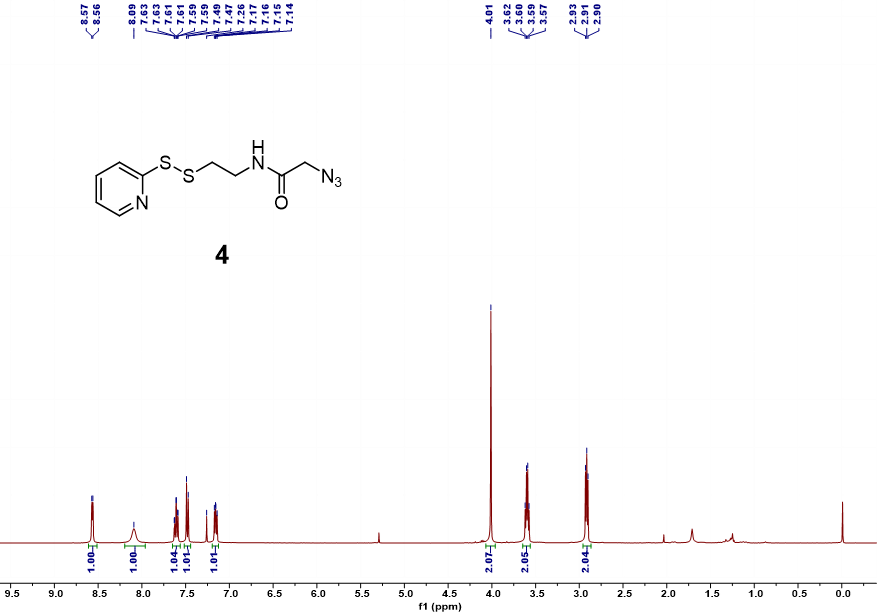


The ^1^H NMR spectrum (400 MHz) of compound **4** in CDCl_3_.


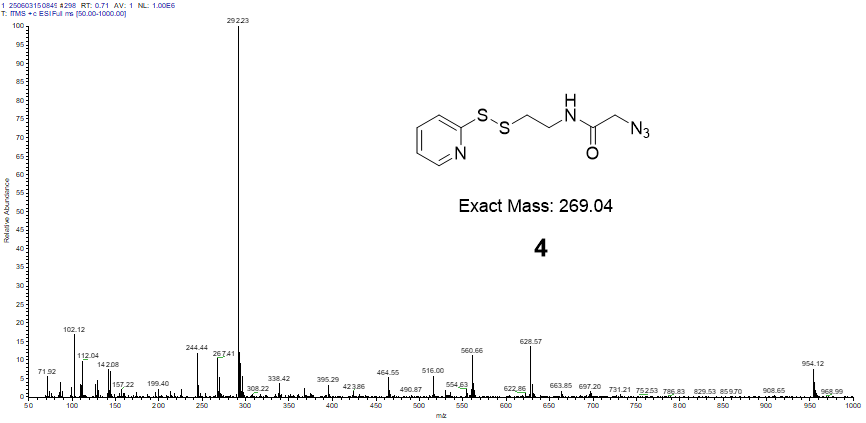


The ESI-MS spectrum of compound **4**.


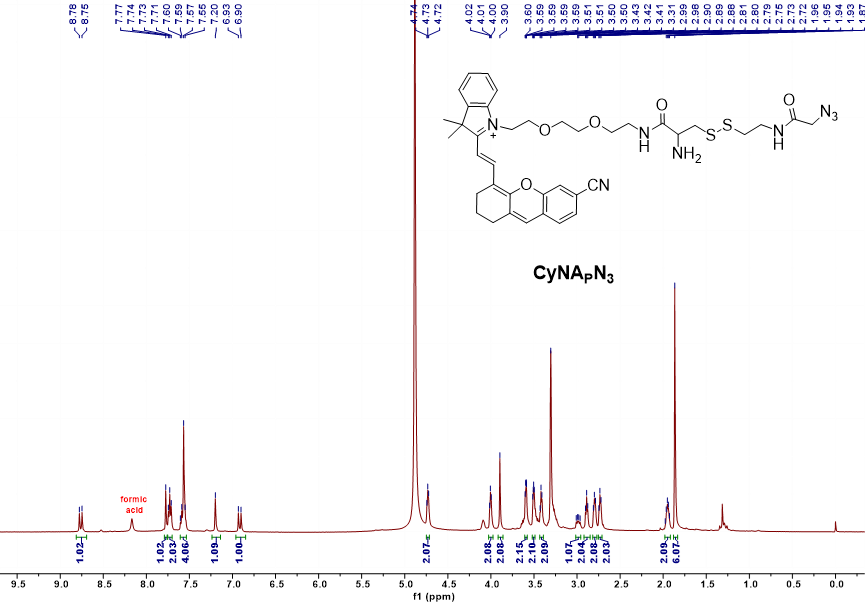


The ^1^H NMR spectrum (500 MHz) of compound **CyNA_P_N_3_** in Methanol-*d*_4_.


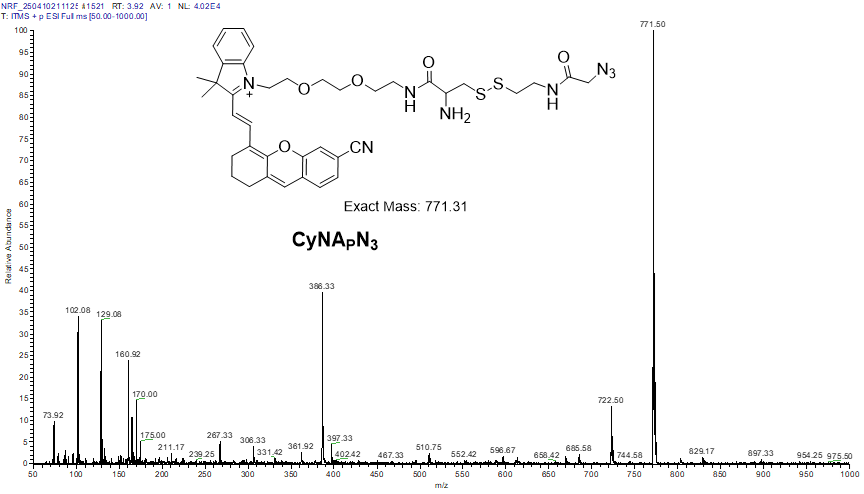


The ESI-MS spectrum of compound **CyNA_P_N_3_**.


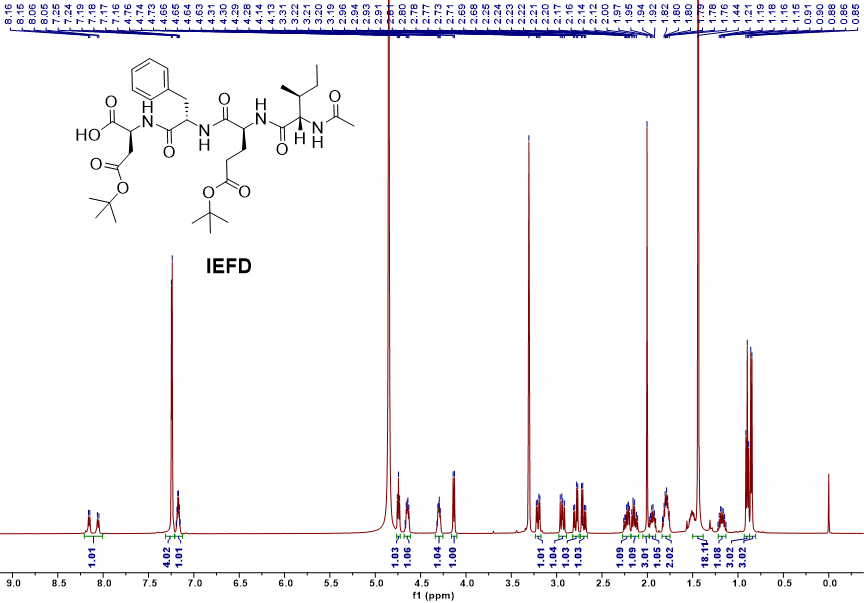


The ^1^H NMR spectrum (500 MHz) of compound **IEFD** in Methanol-*d*_4_.


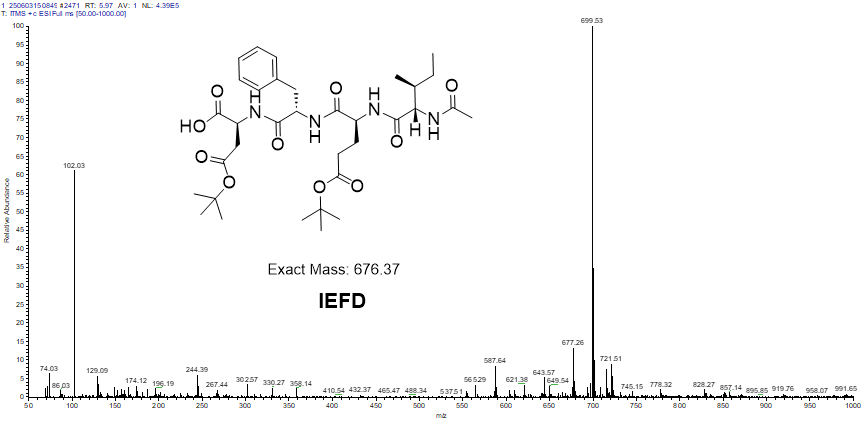


The ESI-MS spectrum of compound **IEFD**.


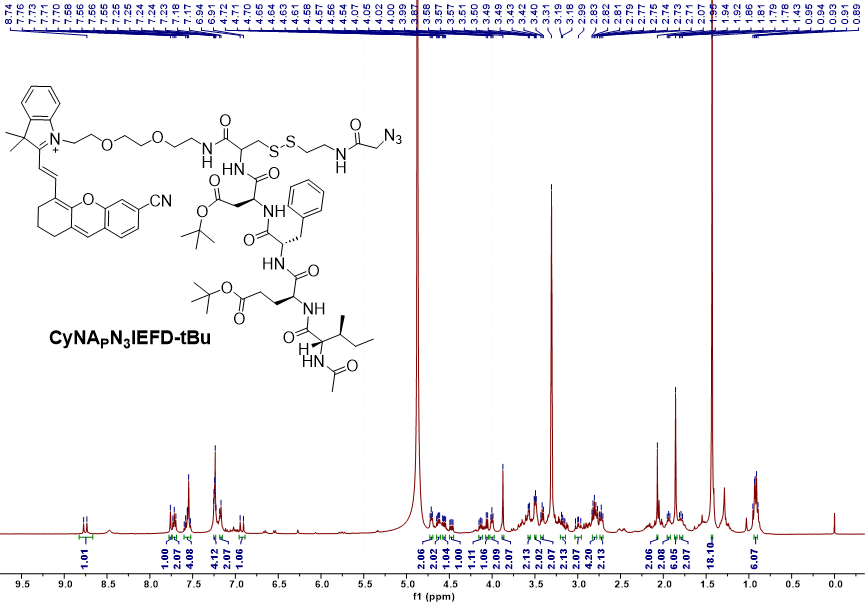


The ^1^H NMR spectrum (400 MHz) of compound **CyNA_P_N_3_IEFD-tBu** in Methanol-*d*_4_.


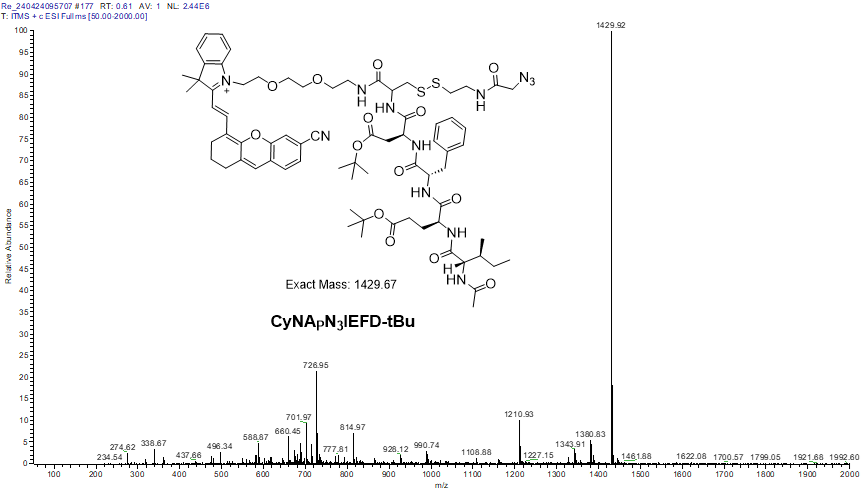


The ESI-MS spectrum of compound **CyNA_P_N_3_IEFD-tBu**.


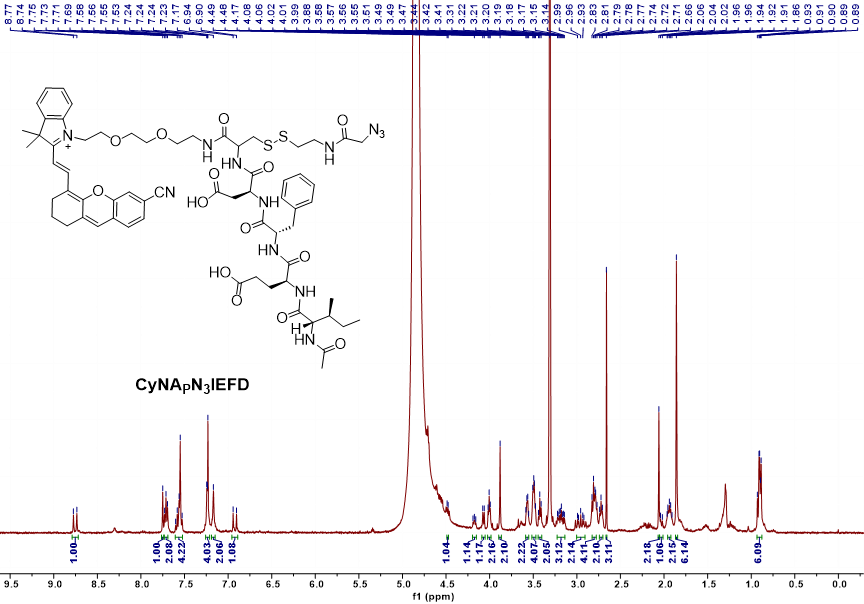


The ^1^H NMR spectrum (400 MHz) of compound **CyNA_P_N_3_IEFD** in Methanol-*d*_4_.


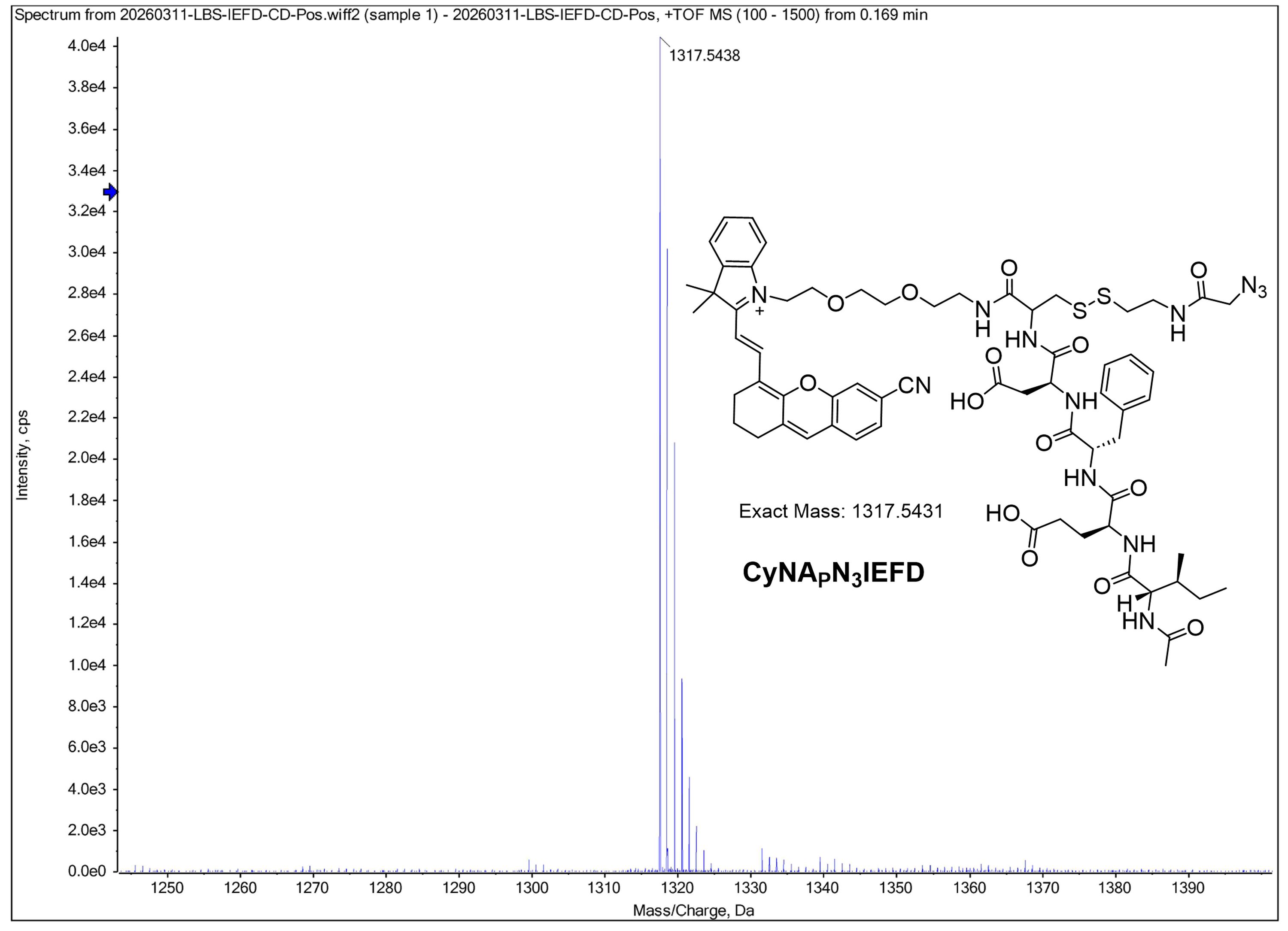


The HRMS spectrum of compound **CyNA_P_N_3_IEFD**.


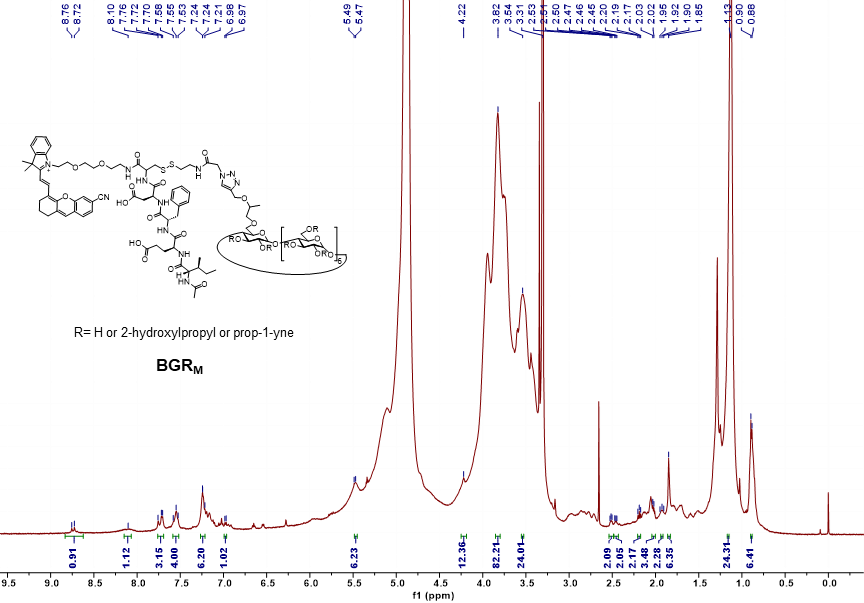


The ^1^H NMR spectrum (500 MHz) of probe **BGR_M_** in Methanol-*d*_4_.


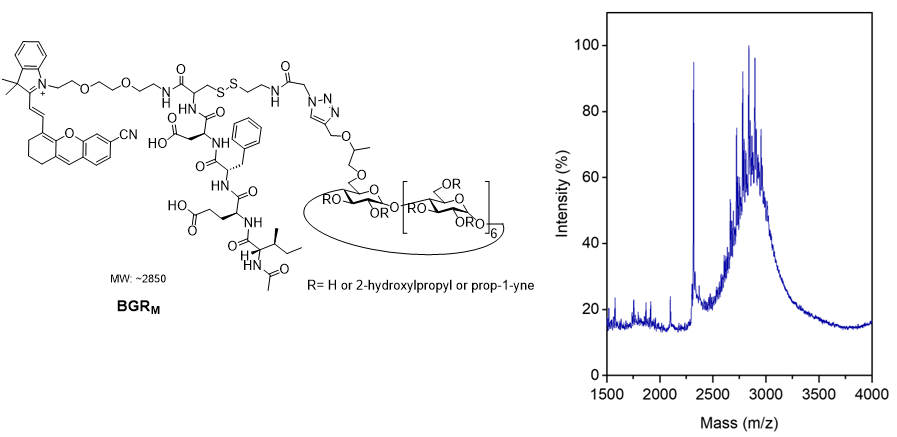


The MALDI-TOF spectrum of probe **BGR_M_**.


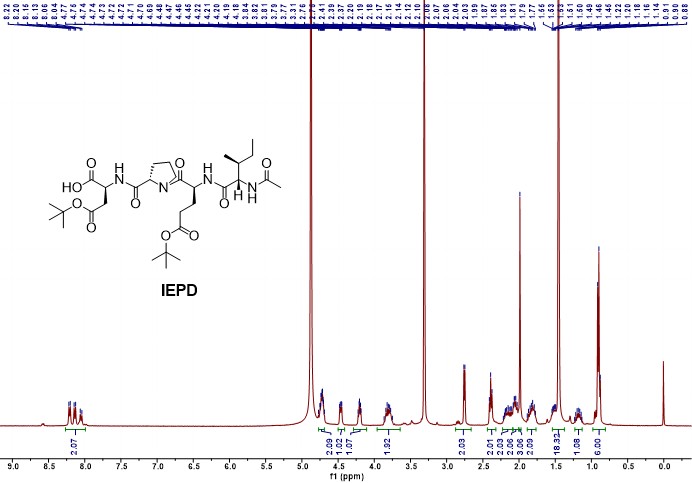


The ^1^H NMR spectrum (400 MHz) of compound **IEPD** in Methanol-*d*_4_.


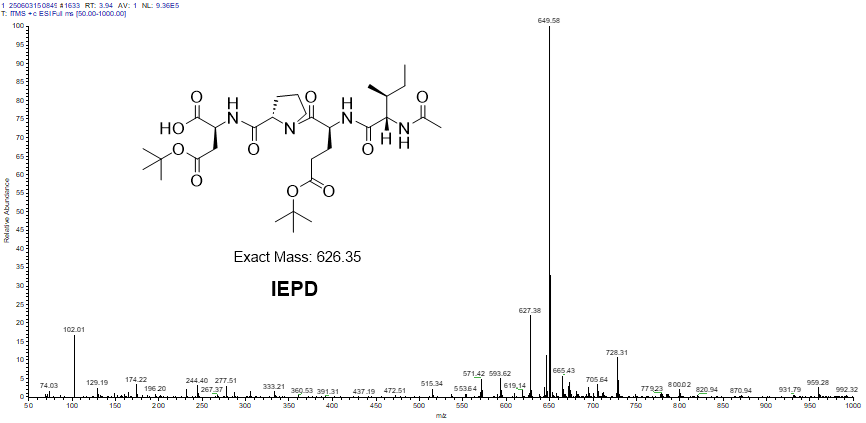


The ESI-MS spectrum of compound **IEPD**.


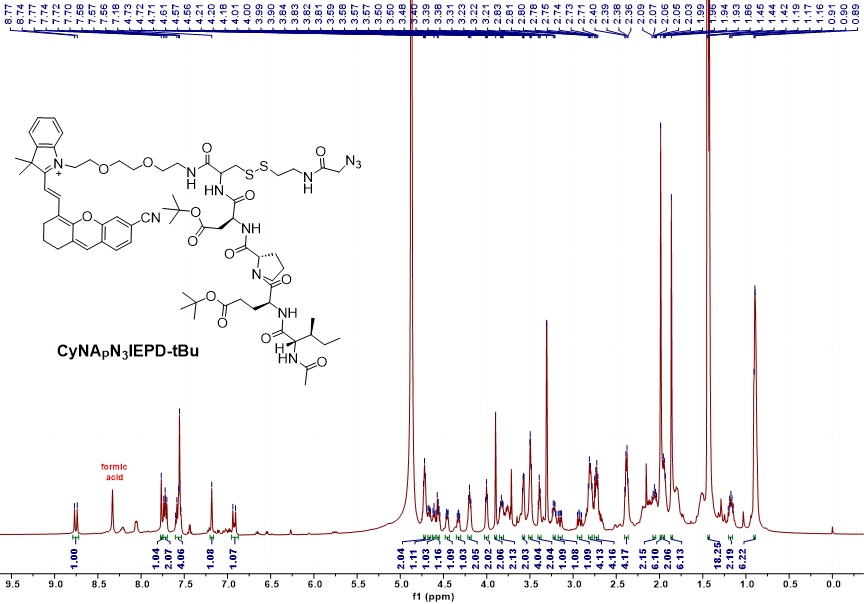


The ^1^H NMR spectrum (500 MHz) of compound **CyNA_P_N_3_IEPD-tBu** in Methanol-*d*_4_.


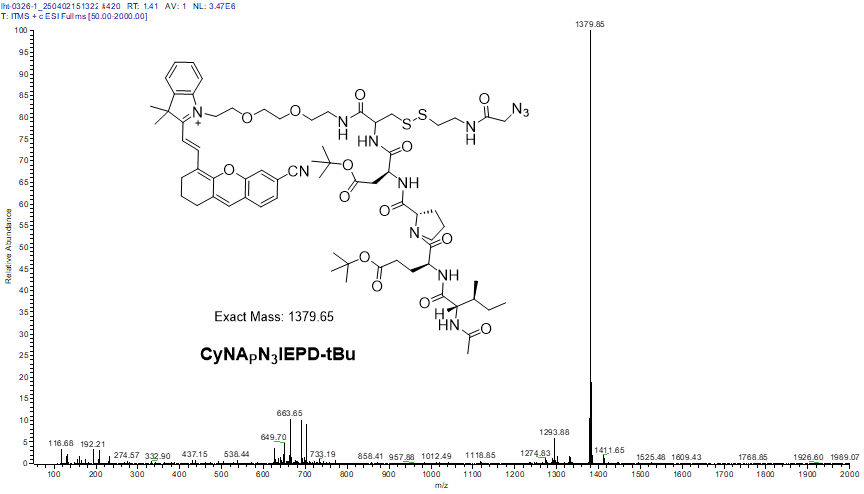


The ESI-MS spectrum of compound **CyNA_P_N_3_IEPD-tBu**.


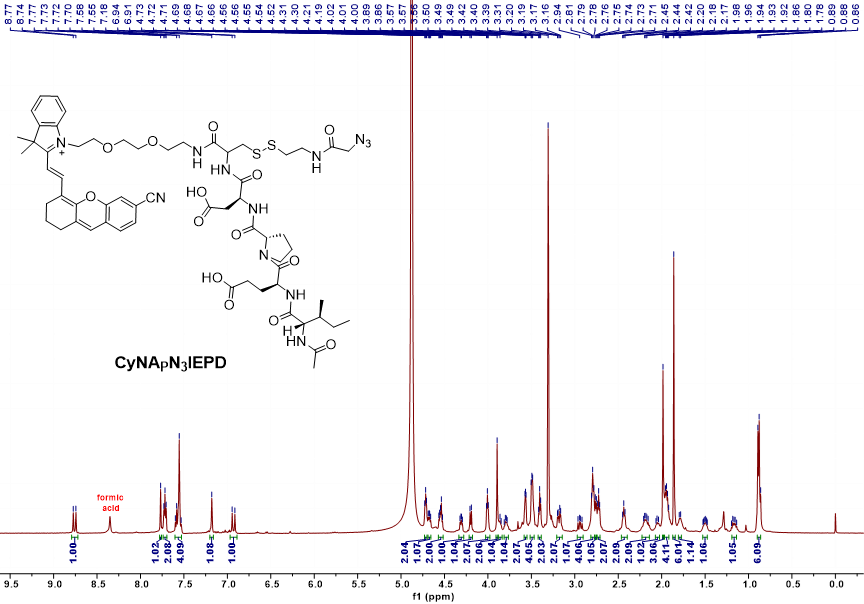


The ^1^H NMR spectrum (500 MHz) of compound **CyNA_P_N_3_IEPD** in Methanol-*d*_4_.


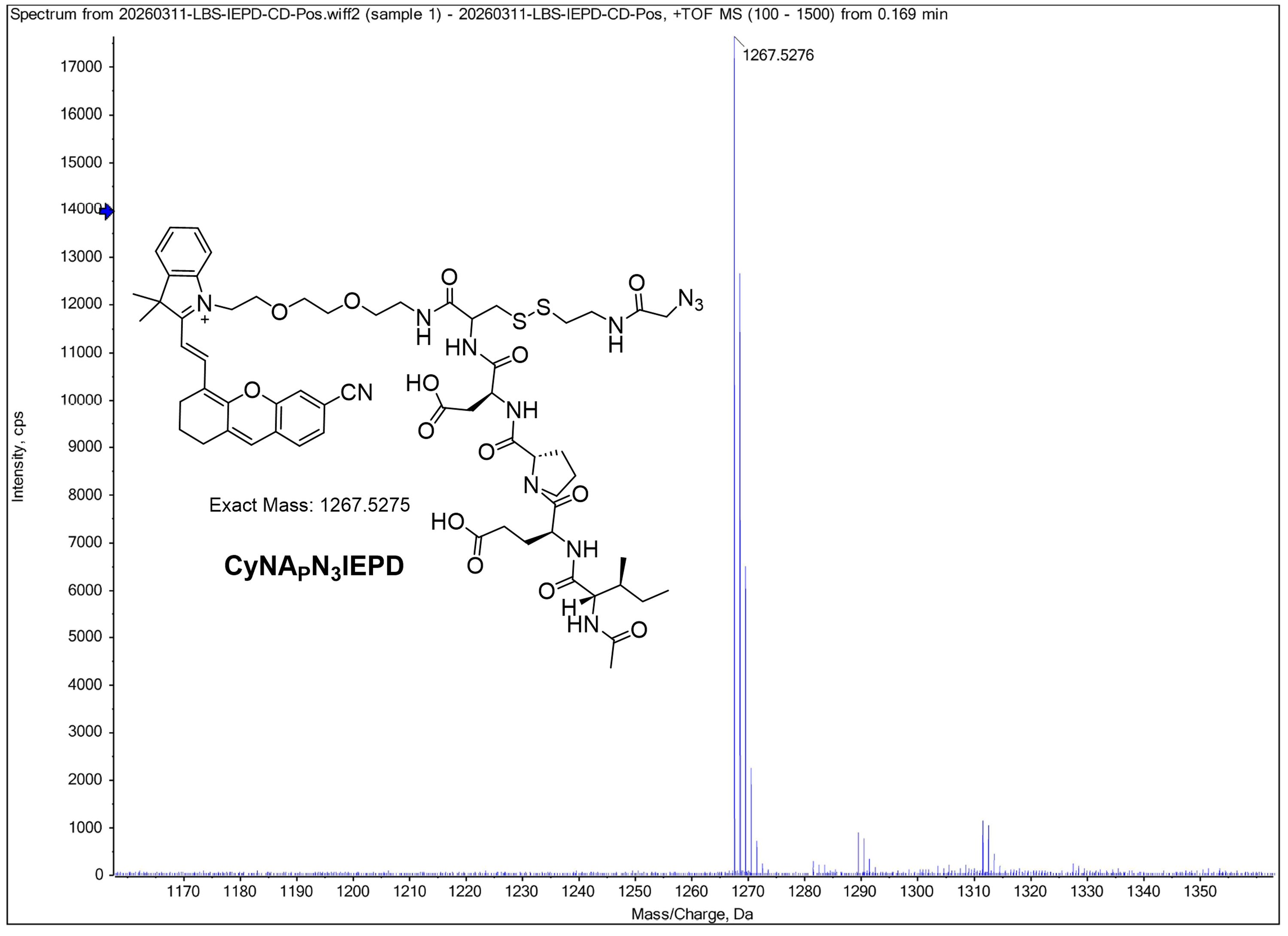


The HRMS spectrum of compound **CyNA_P_N_3_IEPD**.


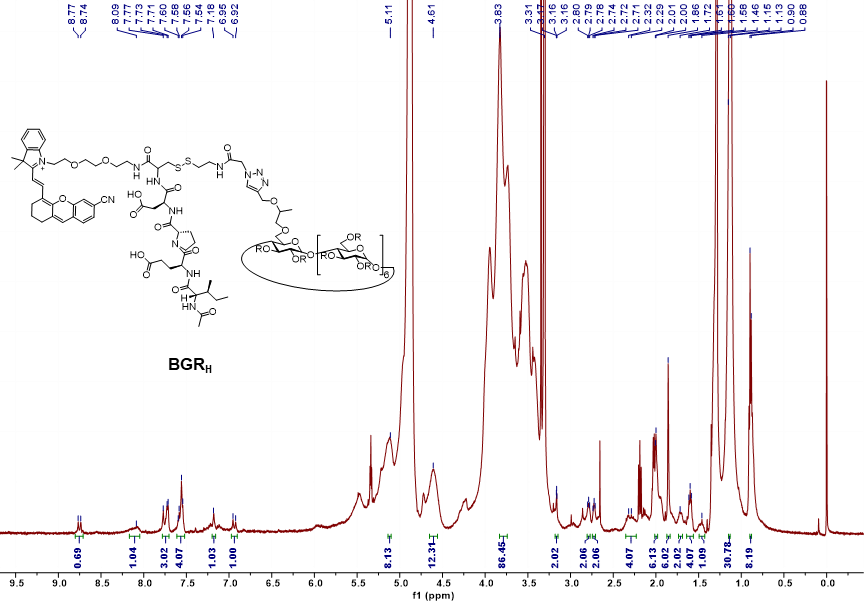


The ^1^H NMR spectrum (500 MHz) of probe **BGR_H_** in Methanol-*d*_4_.


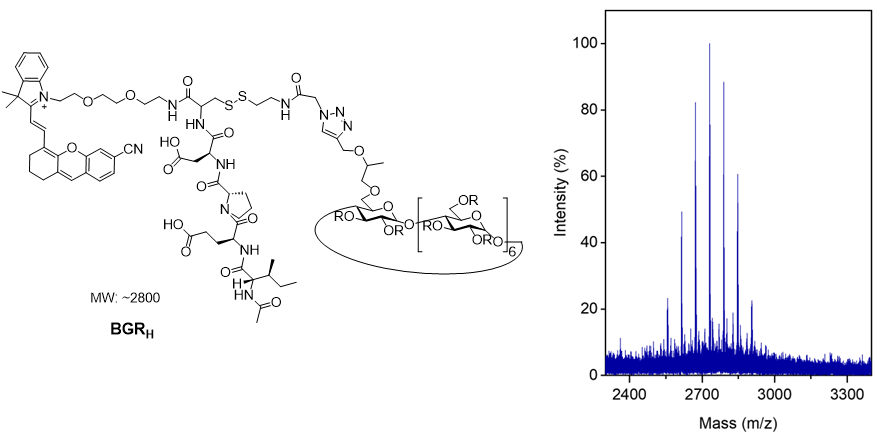


The MALDI-TOF spectrum of probe **BGR_H_**.

**Figure S22** **NMR and MS spectroscopy.**

**4.** **References**

[1] W. Xu, S. Yi, J. Liu, Y. Jiang, and J. Huang, “Nitrile-aminothiol Bioorthogonal Near-Infrared Fluorogenic Probes for Ultrasensitive in Vivo Imaging,” *Nature Communications* 16 (2025): 8.

[2] J. Huang, J. Li, Y. Lyu, Q. Miao, and K. Pu, “Molecular Optical Imaging Probes for Early Diagnosis of Drug-Induced Acute Kidney Injury,” *Nature Materials* 18 (2019): 1133--1143.
